# Modifying Parallel Excitations into One Framework for C(*sp* ^3^)─H Bond Activation with Energy Combined More Than Two Photons

**DOI:** 10.1002/advs.202404293

**Published:** 2024-07-25

**Authors:** Qingbo Shen, Jiali Chen, Xu Jing, Chunying Duan

**Affiliations:** ^1^ School of Chemistry Dalian University of Technology Dalian 116024 China; ^2^ State Key Laboratory of Coordination Chemistry Nanjing University Nanjing 210093 China

**Keywords:** C─H bond functionalization, hydrogen atom transfer, metal–organic frameworks, multiphoton excitation, synergistic catalysis

## Abstract

Natural photosynthesis enzymes utilize energies of several photons for challenging oxidation of water, whereas artificial photo‐catalysis typically involves only single‐photon excitation. Herein, a multiphoton excitation strategy is reported that combines parallel photo‐excitations with a photoinduced electron transfer process for the activation of C(*sp* ^3^)─H bonds, including methane. The metal–organic framework **Fe_3_‐MOF** is designed to consolidate 4,4′,4″‐nitrilotrisbenzoic units for the photoactivation of dioxygen and trinuclear iron clusters as the HAT precursor for photoactivating alkanes. Under visible light irradiation, the dyes and iron clusters absorbed parallel photons simultaneously to reach their excited states, respectively, generating ^1^O_2_ via energy transfer and chlorine radical via ligand‐to‐metal charge transfer. The further excitation of organic dyes leads to the reduction of ^1^O_2_ into O_2_^•−^ through a photoinduced electron transfer, guaranteeing an extra multiphoton oxygen activation manner. The chlorine radical abstracts a hydrogen atom from alkanes, generating the carbon radical for further oxidation transformation. Accordingly, the total oxidation conversion of alkane utilizing three photoexcitation processes combines the energies of more than two photons. This new platform synergistically combines a consecutive excited photoredox organic dye and a HAT catalyst to combine the energies of more than two photons, providing a promising multiphoton catalysis strategy under energy saving, and high efficiency.

## Graphical abstract

Through assembling the Fe_3_ cluster and the organic dye 4,4′,4″‐ Nitrilotrisbenzoic acid in a metal–organic framework material, **Fe_3_‐MOF**. After parallel photoexcitation of **Fe_3_‐MOF**, Cl•, and O_2_^•−^ are produced for functionalizing C(*sp* ^3^)─H bonds. It represents the first example of coordination polymer to accelerate C(*sp* ^3^)─H bonds functionalization combining the energies of more than two photons for parallel excitation.

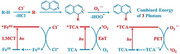

## 1 Introduction

**Scheme 1:**
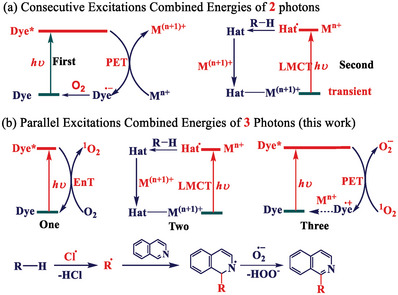
Perspective view of the consecutive excitations a) and the parallel excitations for C─H bonds oxidative conversion b) that combined energies of more than two photons without the excitation of in situ formed transient species.

Photoredox catalysis is an old concept that undergoes single electron transfer from its excited states to generate open‐shell intermediates.^[^ ^1^, ^2^ ^]^ Photocatalysts participate in distinct activation modes that are complementary to the transition‐metal thermo‐catalyzed reactions, guaranteeing a paradigm shift in organic synthesis that incorporates substrate activation and provides access to heretofore elusive reaction pathways.^[^ ^3^ ^]^ To purchase thermodynamically increasing challenging reactions with well‐established photocatalysts, the multiphoton excitation manifold has been exploited to activate inert bonds by combining the energy of two photons per catalytic turnover.^[^ ^4^, ^5^, ^6^, ^7^ ^]^ Nevertheless, the intrinsic instability and shorter lifetime of the transient state (**Scheme** **1**) and the corresponding excited state impede the efficiency of the second excitation, hindering the efficiency of photo‐induced electron transfer under a typical diffusion‐limited paradigm. Consequently, high‐intensity irradiation light is always required to reach the desirable turnover frequency.^[^ ^8^ ^]^ We wonder if the two parallel excitations with separated photon absorption at both active sites would avoid the photoexcitation of in situ formed transient state in consecutive multiphoton excitation and two‐photon excitation absorption, combining the energies of two (or more) photons per catalytic turnover for thermodynamically demanding reactions.

One of the important, yet still sluggish, chemical conversions is the activation and functionalization of C(*sp* ^3^)─H bonds including methane and other light alkanes, to produce high value‐added products due to the inherent inertness of C(*sp* ^3^)─H bonds.^[^ ^9^, ^10^, ^11^ ^]^ Of the reported oxidization strategies, metal–organic frameworks with ordered dye‐loaded bridging ligands and functionalized metal nodes enable the fixation of different functional groups to echo multiple synergistic catalytic processes in tandem.^[^ ^12^, ^13^, ^14^ ^]^ Our group and others have reported on two consecutive excitations for the activation of inert C(*sp* ^3^)─H bonds by integrating photo‐induced electron transfer (PET), ligand‐to‐metal charge transfer (LMCT), and hydrogen atom transfer (HAT) processes.^[^ ^15^, ^16^ ^]^ We envisioned that the combination of light‐driven energy transfer (EnT) to activate oxygen into the active singlet oxygen (^1^O_2_) and photo‐mediated HAT to activate C(sp^3^)─H bonds would modify and disperse highly active sites, controlling the reactivity of C(sp^3^)─H bonds and the selectivity of oxygenation products. In this case, an organic dye could interact with ^1^O_2_ through its excited state (Scheme 1),^[^ ^17^, ^18^ ^]^ we assumed that this multi‐photon excitation strategy would guarantee reactions that combining the energy of more than two photons per catalytic turnover.

Herein we report a powerful crystal engineering manifold to incorporate abundant iron ions and a commercially used organic dye, 4,4′,4″‐Nitrilotrisbenzoic acid (H_3_TCA) into one network for synergistic activation and functionalization of C(*sp* ^3^)─H bonds.

The multiphoton excitation comprised parallel photons that drive an EnT to generate ^1^O_2_ and an LMCT to create chlorine radicals. The former possibly gave the superoxide radical O_2_^•−^ through consecutive excitation of the organic dyes, followed by a PET, the latter abstracts a hydrogen atom from the C(*sp* ^3^)─H bonds to generate a carbon radical intermediate via the HAT process. The oxidation state of the dye (TCA^+^) oxidizes the Fe^II^ species to regenerate the TCA and Fe^III^ species for next cycle, wherein, the O_2_^•−^ further interacted with the intermediates for further oxidation functionalization (Scheme 1b). The total functionalization of alkanes utilizes three photoexcitation processes, combining the energies of more two photons. To the best of our knowledge, this represents the first example of an artificial photocatalyst that can accelerate C(*sp* ^3^)─H bonds functionalization using energies more than two photons that combined with two parallel excitation and a consecutive excitation of the dyes.

## 2 Results and Discussion

**Figure 1:**
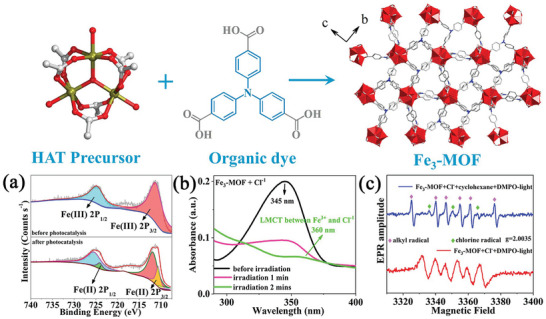
Molecular structure of the iron‐based metal–organic framework **Fe_3_‐MOF** with its assembly. a) High‐resolution XPS spectra of the Fe(2p) for **Fe_3_‐MOF** before and after photocatalysis with pyridine hydrochloride. b) UV–vis spectra of **Fe_3_‐MOF** were added to acetonitrile solution containing 0.02 mg mL^−1^ pyridine hydrochloride under irradiation for different times. c) EPR spectra of **Fe_3_‐MOF** in a methanol solution containing tempo (10 µL) irradiated with 395 nm LED and in the presence (blue) and absence (red) of cyclohexane.

**Fe_3_‐MOF** was prepared using H_3_TCA (Figure S3, Supporting Information) and [Fe_3_(*µ_3_*‐O)(CH_3_COO)_6_(H_2_O)_3_] as the raw materials by a solvothermal method, with a yield of 35%. Single crystal X‐ray diffraction analysis reveals that **Fe_3_‐MOF** crystallizes in an orthorhombic space group *Pca*2_1_ (Table S1, Supporting Information). Like those reported structures,^[^ ^19^, ^20^ ^]^ the Fe_3_ cluster consists of three Fe^3+^ ions, one µ_3_‐O^2−^, six OAc^−1^ group, and three coordination water molecules (Figure S4, Supporting Information). Each TCA ligand links to three Fe_3_ clusters and each Fe_3_ cluster links to six TCA ligands to consolidate the three‐dimensional network (Figures S5–S9, Supporting Information). Elemental‐mapping images show the uniform distributions of Fe, C, N, and O elements in **Fe_3_‐MOF** (Figure S11, Supporting Information). **Fe_3_‐MOF** exhibits large pores with 68% free volume, allowing the ingress and egress of substrates with suitable molecular size (Figures S53 and S54, Supporting Information). ^1^H NMR and infrared spectra of **Fe_3_‐MOF** soaked in the cyclohexane solution show the characteristic peaks of the substrates, supporting that the substrates can freely diffuse into the pores of **Fe_3_‐MOF** (Figures S16–S22, Supporting Information).^[^ ^21^ ^]^ High‐resolution X‐ray photoelectron spectroscopy spectra of **Fe_3_‐MOF** exhibit two peaks at 724.7–711.2 eV with a satellite peak at 718.2 eV attributed to Fe^III^ ions (**Figure** **1**a).^[^ ^22^, ^23^ ^]^

Solid‐state UV–vis spectra of **Fe_3_‐MOF** exhibit a red shift compared to the ligand, reflecting the good absorption capacity of **Fe_3_‐MOF** (Figure S14, Supporting Information).^[^ ^24^ ^]^ Soaking **Fe_3_‐MOF** in different organic solvents for 24 h did not change its powder x‐ray diffraction (PXRD) pattern (Figure S12, Supporting Information). **Fe_3_‐MOF** maintains its thermal stability up to 350°C in thermogravimetry analyses (Figure S13, Supporting Information). **Fe_3_‐MOF** suspensions in acetonitrile exhibited an absorption band at 345 nm, adding pyridine hydrochloride with increased concentration led to the appearance of a new band centered at 360 nm (Figure 1b; Figure S23, Supporting Information). We assigned this band to the LMCT absorption from chloride anion to the Fe^3+^ nodes (Figures S24–S27, Supporting Information).^[^ ^25^ ^]^ The irradiation light on the solution caused a direct intensity diminish of initial absorption band, it is likely that this excitation provided the reduced iron ion Fe^2+^ and chloride radicals for the activation of C(*sp* ^3^)─H bonds via the HAT process.^[^ ^26^ ^]^ High‐resolution X‐ray photoelectron spectroscopy (XPS) spectra of the **Fe_3_‐MOF** after irradiation show the appearance of two peaks at 724.1 and 710.6 eV, indicating the formation of Fe^II^ species during the excitation process (Figure 1a).^[^ ^27^ ^]^

With the presence of the free radical trapping agent 2,2,6,6‐tetramethyl‐piperidine‐1‐oxyl (TEMPO), the chlorine radical was detected by MS after illumination of the reaction system consisting of **Fe_3_‐MOF** and pyridine hydrochloride (Figure S28, Supporting Information). The electron paramagnetic resonance spectra of **Fe_3_‐MOF** with chloride and cyclohexane under N_2_ atmosphere exhibit characteristic peaks of chlorine radicals and carbon radicals (Figure 1c).^[^ ^28^ ^]^ The radical trapping experiments with TEMPO also demonstrated the appearance of carbon radicals generated from alkanes with ^1^H NMR and GC‐MS analysis (Figures S29–S34, Supporting Information).^[^ ^29^ ^]^ We infer that the irradiation of the LMCT band in the **Fe_3_‐MOF** can efficiently activate the C(*sp* ^3^)─H bonds in the presence of chloride anion.^[^ ^30^ ^]^

Subsequently, we investigated the catalytic performance of **Fe_3_‐MOF** in activating and functionalizing inert C(*sp* ^3^)**─**H bonds. In the presence of 0.5 moL% **Fe_3_‐MOF**, treatment of cyclohexane and DBAD with 4.5 moL % pyridine hydrochloride as HAT catalyst precursor under N_2_ atmosphere in acetonitrile and irradiation with a 30w 395 nm LED afforded C─N coupling product with a yield of 98% in 2.5 h as shown in (Tables S3, S5 and Figure S1, Supporting Information). In the absence of **Fe_3_‐MOF**, additive or light source, no or less products could be detected by the catalytic systems (entries 2–4, Table S3, Supporting Information). Longer wavelength light leads to a reduced catalytic conversion or very few reactions (entries 5–6, Table S3, Supporting Information). Control experiments demonstrated that the physical mixture of ferric salts, the ligands and pyridine hydrochloride provided a slightly lower yield for **Fe_3_‐MOF** (entry12, Table S3, Supporting Information), the using pyridine hydrobromide to replace pyridine hydrochloride, did not give the target product (Table S4, Supporting Information). Cyclic alkanes were catalyzed with excellent yields (see 1–4, **Table** **1**). C(*sp* ^3^)─H bond activation of chain alkanes is also obtained with satisfactory yields (5, Table 1). Particularly, **Fe_3_‐MOF** endowed the ethane into relative C─N products at 0.5 MPa pressure with a yield of 93% (6, Table 1; Figure S2, Supporting Information).

**Table 1:** **Fe_3_‐MOF** photocatalytic functionalization of inert C(sp^3^)─H bonds.

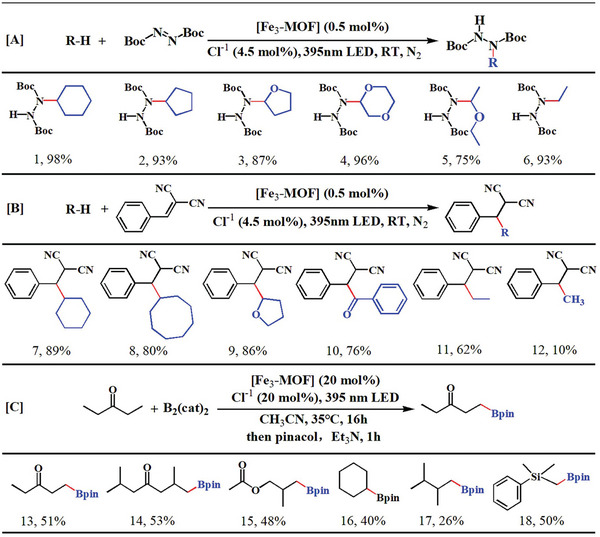

[A] [B] Reaction condition: C(*sp* ^3^)─H bonds substrate (2 mmoL), radical trap (0.2 mmoL), **Fe_3_‐MOF** (0.5 mmoL%), pyridine hydrochloride (4.5 moL%) in 2 mL CH_3_CN. under irradiation with a 30w 395 nm LED under argon atmosphere at room temperature. The reaction times of [A] and [B] were carried out within 3 h and 12 h, respectively. The pressure of the gaseous C(*sp* ^3^)─H bonds substrate is 0.5 to 3 Mpa. [C] Reaction condition: C(*sp* ^3^)─H bonds substrate (2 mmoL), B_2_(cat)_2_ (0.2 mmoL), **Fe_3_‐MOF** (20 mmoL%), pyridine hydrochloride (20 moL%) in 2 mL CH_3_CN under irradiation with a 10w 395 nm LED under argon atmosphere at room temperature. The yields of the products were determined by ^1^H NMR using dibromomethane as an internal standard.

When another radical trapping agent, benzylidene malononitrile, was used, **Fe_3_‐MOF** could activate methane to form a C─C coupling product with a yield of 10% (12, Table 1). Several substrates also obtained good activation yields when trapped under the same manifolds (7–11, Table 1). Comparing to the reported HAT catalysts for the activation of C(*sp* ^3^)─H,^[^ ^29^, ^31^ ^]^ **Fe_3_‐MOF** has good recyclability, a slight decrease in the yield of C─N coupling product was observed after four cycles (Figure S35, Supporting Information). Selective boronation of C(*sp* ^3^)─H bonds has always been an interesting frontier in various scenarios because organo‐boron compounds are crucial building blocks in industry and the academic laboratory.^[^ ^32^ ^]^ We then validated the catalytic activity towards C(*sp* ^3^)‐H using 3‐pentanone and bis(catecholato)diboron (B_2_(cat)_2_) as initial substrates and observed a yield of 51% for the borylated product under optimal conditions (Table S6, Supporting Information). Other ketones obtained satisfactory yields (14 and 15, Table 1), and the commonly used alkanes and silanes can be compatible with the reaction condition to obtain the desired borate products and satisfactory yields (16–18, Table 1). These results all revealed that **Fe_3_‐MOF** was promising for C(*sp* ^3^)─H bond functionalization.

**Figure 2:**
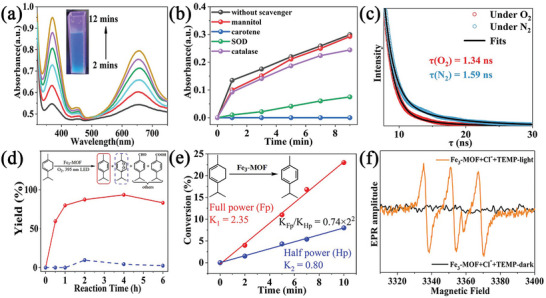
a) UV–vis spectra of **Fe_3_‐MOF** in the presence of 3,3′,5,5′‐tetramethylbenzidine (TMB) and Cl^−1^ in air under 395 nm LED irradiation. b) UV–vis spectra for TMB oxidation product monitored at 650 nm along with reaction time over **Fe_3_‐MOF** in the presence of different scavengers under 395 nm LED irradiation. c) Luminescence decays of **Fe_3_‐MOF** suspensions under N_2_ (blue line) and (red line) O_2_ atmosphere. d) Kinetic profiles of the oxidation of a‐terpinene to 4‐isopropyltoluene (solid red line) and ascaridole (dotted blue line) catalyzed by **Fe_3_‐MOF** under 395 nm LED irradiation. e) Two oxidation conversions of a‐terpinene as a function of the time under the standard conditions with a 395 nm LED irradiation of full power and half power within 10 min, respectively. f) EPR spectra of **Fe_3_‐MOF** (2 mg) in an acetonitrile solution containing TEMP (10 µL) under dark (black color) and irradiation with a 395 nm LED (brown color).

We next investigate **Fe_3_‐MOF** for oxygen activation. When 3,3′,5,5′‐tetramethylbenzidine (TMB), an indicator of the reactive oxygen species (ROS),^[^ ^33^ ^]^ was added to the suspension of **Fe_3_‐MOF**, the absorption band at 650 nm appeared and enhanced with the light irradiation (**Figure** **2**a; Figure S36 and S37, Supporting Information). Introducing mannitol and catalase, the ROS inhibitors for •OH and H_2_O_2_, respectively, to the **Fe_3_‐MOF** suspension did not cause obvious inhibition of the TMB oxidation (Figure 2b). However, the presence of superoxide dismutase and carotene inhibited the oxidation of TMB in the suspension of **Fe_3_‐MOF**.^[^ ^34^ ^]^ Furthermore, the emission lifetime of **Fe_3_‐MOF** gradually diminishes under O_2_ atmosphere (Figure 2c). The photocurrent response in oxygen is significantly higher than that in nitrogen (Figure S38, Supporting Information). Both the ^1^O_2_ and O_2_^•−^ are likely to be major ROS, dominating the photocatalytic performance. Since ɑ‐Terpinene is very sensitive to ROS, photo‐catalytic oxidation of ɑ‐Terpinene was chosen as another model.^[^ ^35^ ^]^ Under optimized conditions, the loading 0.5 mol% **Fe_3_‐MOF** lead to a conversion of 80% of the associated‐O_2_^•−^ product *p*‐cymene after irradiation for 1 h, but few of the associated‐^1^O_2_ product ascaridole could be detected (Figure 2d).

**Figure 3:**
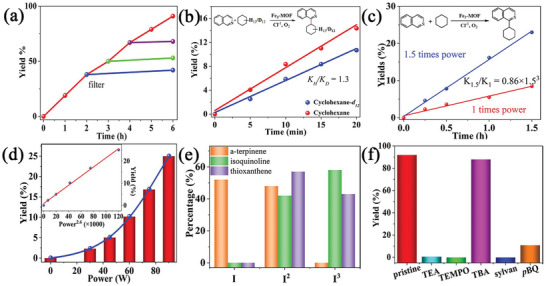
a) Time‐dependent conversion of the photocatalytic arylation of cyclohexane with filter catalysts within 6 h. b) Parallel kinetic isotope effect (KIE) experiment for the arylation of cyclohexane and cyclohexane‐*d_12_* (2 mmoL) within 20 min under standard conditions. c) Two arylation reaction yields of cyclohexane as a function of the time under the standard conditions with a 395 nm LED irradiation of 1.5 times power and 1 time power within 1.5 h, respectively. d) The arylation reaction yields cyclohexane as a function of irradiation powers under the standard conditions with a 395 nm LED irradiation within 30 min. Inset shows the linear relationship between the yields of the photocatalysis and the times n of photon powers. e) The proportion of single photons and two photons excitation in the oxidation kinetics of a‐terpinene, and the proportion of two photons and three photons excitation in the catalytic oxidation kinetics of isoquinoline and thioxanthene. f) Yield of diphenylmethane oxidation using **Fe_3_‐MOF** in the absence (pristine) or presence of different scavengers (1 equiv.).

Controlled experiments exhibited that the physical mixture of FeCl_3_ and ligand also gave the associated‐O_2_^•−^ product *p*‐cymene (Table S7, Supporting Information). It seems that the organic dye in **Fe_3_‐MOF** first absorbed one photon to reach its excited state, giving the ^1^O_2_ species through photoinduced EnT,^[^ ^36^ ^]^ the high activity of the ^1^O_2_ quickly reacted with another reduction active species in the reaction mixture, generating O_2_^•−^ species for oxidation conversion. Photon power dependence experiments revealed an approximate of 3.0 times increase in conversion when the irradiation power was 2.0 times (Figures 2e; Figure S39, Supporting Information). Besides the photo‐induced EnT from the ligand's excited state, it is likely that other excitation processes participated oxygen activation to form O_2_^•−^. The new catalytic process was assumed to the interaction between the excited state of the dye‐based ligand in **Fe_3_‐MOF** and the in situ formed ^1^O_2_ within the confined pores.^[^ ^37^ ^]^ Electron paramagnetic resonance of **Fe_3_‐MOF** with ^1^O_2_ trapping agent TEMP and O_2_^•−^ trapping agent DMPO, respectively, gave the characteristic peaks related to those of ^1^O_2_ and O_2_^•−^ adducts after light illumination (Figures 2f and 1c).^[^ ^38^, ^39^ ^]^ We also recognized that the product yield did not exhibit the exact correlation with the quadratic of the photon power. It is postulated that there is a single photon excitation activation pathway contributes to the generation of O_2_^•−^, besides two photons consecutive excitations to activate dioxygen via the formation of singlet ^1^O_2_ as an intermediate (**Figure** **3**e). As the almost same catalytic results, giving the associated‐O_2_^•−^ product *p*‐cymene, when free ligand was used for dioxygen activation, we infer that a typical photoinduced EnT corresponding to the excited state of ligands in the frameworks contributes to generating of O_2_^•−^, which exhibited a linear relationship between the oxidation product yield and the photon energy. If we defined a fraction *x* (calculated *ca* 48%) to describe the contribution of the two‐photon excitation (Table S12, Supporting Information), a value of 52% was obtained for the monophoton excitation.

Next, we choose the C─C coupling reaction between cyclohexane and isoquinoline as the template reaction for the arylation of alkanes. Under the optimum condition, in the presence of 0.5 moL% **Fe_3_‐MOF**, adding 4.5 moL% pyridine hydrochloride, the yield of C─C coupling product reached 91% in 6 h under air atmosphere with irradiation from a 30 w 395 nm LED (Figure 3a; Tables S8 and S9, Supporting Information). Introducing different substituents to isoquinoline, the products also obtained in satisfactory yields (20 and 21, **Table** **2**). Cyclohexane can also be arylated by some other *N*‐containing heterocyclic compounds with better yields (22 and 23). We have also expanded the range of inert C(*sp* ^3^)─H bonds substrates and obtained a satisfactory yield of arylated products of cyclic ether (24 and 25), especially straight chain ether, the product 26 of ether arylated by isoquinoline is a key scaffold in drug molecules.^[^ ^40^ ^]^

**Table 2:** Substrate scope for the photo‐induced Fe‐catalysed C(*sp* ^3^)─H oxidation.

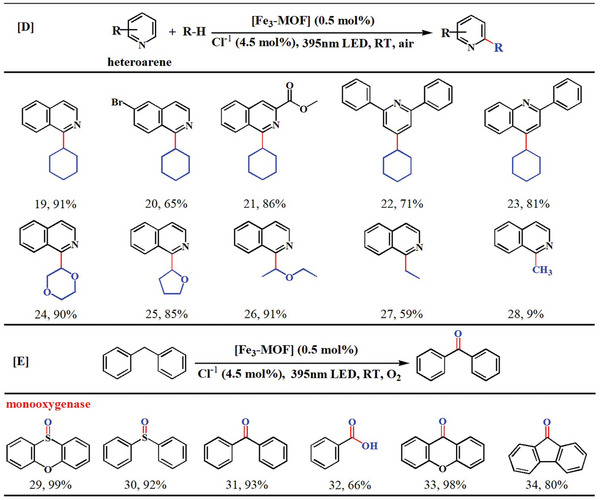

[D] Reaction conditions: C(*sp* ^3^)─H bonds substrate (2 mmoL), heteroarene (0.2 mmoL), **Fe_3_‐MOF** (0.5 mmoL%), pyridine hydrochloride (4.5 moL%) in 2 mL DCM under irradiation with a 30w 395 nm LED under air at room temperature. [E] Reaction conditions: C(*sp* ^3^)─H bonds substrate (0.2 mmoL), **Fe_3_‐MOF** (0.5 mmoL%), pyridine hydrochloride (4.5 moL%) in 2 mL CH_3_CN under irradiation with a 30w 395 nm LED under oxygen atmosphere at room temperature. The yields of the products were determined by ^1^H NMR using dibromomethane as an internal standard.

Furthermore, the inert gaseous alkanes methane and ethane can also be arylated to their corresponding products (27 and 28). A kinetic isotope effect (KIE) experiment gave a *K_H_*/*K_D_* value of 1.3 in the C─C coupling reaction between cyclohexane and iso‐quinoline, indicating that the C─H bond cleavage of cyclohexane is not a rate‐determining step (Figure 3b).^[^ ^41^, ^42^ ^]^ Photon intensity‐dependent experiments of the C─C coupling exhibited 2.89 times increase in conversion with 1.5 times increase in irradiation power (Figure 3c,d). We deduced that with the additional photon excitation involved, the oxidization C─C coupling reaction combined the energy of more than two photons per catalytic turnover, as ideal three photons multi‐excitation should have 1.5^3^ (3.375) rate enhancing. To the best of our knowledge, this is the first example of multiphoton excitations for the activation of inert substrates, guaranteeing the advantage of multi‐photon excitation in chemical conversions. The catalyst exhibits sufficient stability, as the conversion rate does not decrease even after 5 rounds of catalytic reactions. (Figures S10–S46, Supporting Information).

**Scheme 2:**
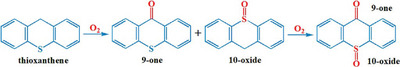
Photooxidation conversion sequence of thioxanthene.

To further explore the mechanism of multiphoton excitation, thioxanthene that has two oxidation sites,^[^ ^43^ ^]^ susceptible to producing thioxanthene‐9‐one (associated‐O_2_^•−^) and thioxanthene‐10‐oxide (associated‐^1^O_2_), was chosen as another model substrate (**Scheme** **2**). As shown in Figure S41 (Supporting Information), the ketone product is first formed within 1 h, and the full oxidization product thioxanthene‐9‐one‐10‐oxide is produced for 3 h (Figure S42, Supporting Information), which indicated that ^1^O_2_ and O_2_^•−^ were both considered as main reactive oxygen species in the reaction process, but O_2_^•−^ was more inferred than that of ^1^O_2_ to integrate with the photomediate HAT for C(sp^3^)─H bonds activation, as no thioxanthene‐10‐oxide was detected during the whole catalytic oxidation. Importantly, photon power‐dependent experiments of the oxidation yield of thioxanthene‐9‐one exhibited 2.75 times (ideal 1.5^3^ is 3.375) increasing, when the irradiation light power increased 1.5 times (Figure S40, Supporting Information), but the yield of thioxanthene‐9‐one‐10‐oxide exhibited a simple linear relationship with that of the photon power (Figure S43, Supporting Information). We thus deduced that more than two parallel excitations were involved in the formation of the ketone product, but only one kind of excitation was found in the conversion of the full oxidation product from the initial ketone product. Using the same fraction definition, a value of 43% was obtained for the contribution of 3 photons excitation (Figure 3e; Table S12, Supporting Information). The value is quite similar to that of the dioxygen activation reaction, we deduced that the first time observed 3 photon excitation reaction originated from the 2 photon excitation dioxygen activation, but plus the parallel monophoton LMCT excitation to drive the C─H bond cleavage.

From a mechanistic point of view, the irradiation of **Fe_3_‐MOF** with chloride ions initially caused two parallel excitations. One drives an EnT from the excited state of organic dye in the polymers to dioxygen, generating ^1^O_2_ species, the other is a LMCT from Cl^−^ to Fe^3+^ species, giving chloride radial. The third excitation involves the in situ formed ^1^O_2_ species to be further activated into O_2_^•−^ through a PET from the excited state of the organic dye. Wherein the chlorine radical abstracts a hydrogen atom from C(*sp* ^3^)‐H bonds to generate a carbon radical, but no in situ formed transient state (Scheme 1) that always exhibited intrinsic instability and shorter lifetime were excited, guaranteeing the efficiency of these parallel excitation. The oxidation state of the organic dye (TCA^+^) oxidized the Fe^II^ species to regenerate TCA and Fe^III^ species for the next cycle (Figure S15, Supporting Information), wherein O_2_^•−^ interacted with the reaction inter‐mediates to complete the oxidation reaction.

We next extend our oxidation mainfold to diphenylmethane by loading **Fe_3_‐MOF** (0.5 moL%), 4.5 moL% pyridine hydrochloride, irradiating with a 395 nm LED for 3 h under an oxygen atmosphere, a 93% yield and 99% selectivity were obtained (Table S10, Supporting Information). The conversion rate barely changed after **Fe_3_‐MOF** was reused 4 times, revealing the high stability of **Fe_3_‐MOF** (Figure S47, Supporting Information). We also recognized the addition of TEMPO completely inhibited the catalytic oxidation of diphenylmethane reaction, Tert‐butanol (TBA, •OH scavenger) does not quench the reaction, but addition 2‐methylfuran (sylvan, ^1^O_2_ scavenger)^[^ ^44^ ^]^ and *p*‐benzoquinone (*p*BQ, O_2_^•−^ scavenger) significantly reduces the conversion yield, further supported that both ^1^O_2_ and O_2_^•−^ are the important reactive oxygen species activated by **Fe_3_‐MOF** (Figure 3f). Other aromatic activated C(sp^3^)─H bonds could be selectively oxidized in ketones (29–34, Table 2).

Many oxygenases have roles in reactions to break C(*sp* ^3^)‐ C(*sp* ^3^) bonds, especially when hydrogen atoms are attached to them.^[^ ^45^ ^]^ These fall into a general mechanistic pattern in the formation of unstable products that break C─C bonds during decomposition. Interestingly, **Fe_3_‐MOF** has a similar metabolic role as Natural oxidase P450. As **Fe_3_‐MOF** has a strong ability to activate oxygen under multiple photoexcitation to the simultaneous formation of ^1^O_2_ and O_2_^•−^, it enabled the breaking of C(*sp* ^3^)‐C(*sp* ^3^) bonds completely, until the C(*sp* ^3^) is terminal attached to an aromatic ring. In the optimized condition, the aliphatic branch of substrates could be metabolized continuously into aromatic acids (Table and Figures S48–S52, Supporting Information), like those of the native oxidases, that is., cyctochrome P450.^[^ ^46^ ^]^

## 3 Conclusion

In summary, we developed a multiphoton excitation catalytic approach for activating and functionalizing inert C(*sp* ^3^)─H bonds including methane by incorporating an organic dye and a HAT precursor in one metal–organic framework. **Fe_3_‐MOF** facilitated parallel EnT and LMCT processes by reaching the excited state of the organic dye and the iron center, respectively, for the generation of ^1^O_2_ and active chlorine radicals. The chlorine radical abstracted an H‐atom via HAT process. The third excitation converted the in situ formed ^1^O_2_ species into the O_2_^•−^ via a typical PET, further oxidizing the intermediates to complete the oxidation transformation, combining the energy of more than two photons per catalytic turnover. And no in situ formed transient state (Scheme 1) that always exhibited intrinsic instability and shorter lifetime was excited, guaranteeing the efficiency of these parallel excitations. We believe that the promising approach will bring inspiration to the modification of multiphoton excitation catalytic platforms for efficient and thermodynamics‐demanding reactions.

## Conflict of Interest

The authors declare no conflict of interest.

## Supporting information

Supporting Information

## Data Availability

The data that support the findings of this study are available in the supplementary material of this article.
