# Supplementary material for: Modifying Parallel Excitations into One Framework for C(sp 3)─H Bond Activation with Energy Combined More Than Two Photons
Source: Adv Sci (Weinh). 2024 Jul 25;11(36):2404293. doi: 10.1002/advs.202404293 (PMC11423249; doi:10.1002/advs.202404293)
Supplement: Supplementary file 1 — Supporting Information [file ADVS-11-2404293-s001.pdf]

ADVANCED SCIENCE
Open Access

Supporting Information

for *Adv. Sci.*, DOI 10.1002/advs.202404293

Modifying Parallel Excitations into One Framework for C(*sp*^{3})—H Bond Activation with Energy Combined More Than Two Photons

Qingbo Shen, Jiali Chen, Xu Jing and Chunying Duan**

Supporting Information
©Wiley-VCH 2021
69451 Weinheim, Germany

Modifying Parallel Excitations into One Framework for C(sp^3)-H Bond Activation with Energies Combined More Than 2 Photons

Qingbo Shen, Jiali Chen, Xu Jing,* Chunying Duan*

Table of contents

1. Materials and Methods.

2. Preparation of **Fe_3-MOF**.

3. Single Crystal X-ray Crystallography.

4. Characterization of **Fe_3-MOF**.

5. Characterization of LMCT Process between Cl^{-1} Ions and **Fe_3-MOF**.

6. Substrate Encapsulation Experiments of **Fe_3-MOF**.

7. Data Relative to Photocatalytic Activation of Inert C(sp^3)-H Bonds.

8. Data Relative to Photocatalytic Borylation of Inert C(sp^3)-H Bonds.

9. Activated Oxygen Performance.

10. Data Relative to Photocatalytic Arylation of Inert C(sp^3)-H Bonds.

11. Data Relative to Photocatalytic Oxidation of Inert C(sp^3)-H Bonds.

12. NMR and GC-MS spectra for oxidized products.

13. References.

1. Materials and Methods

1.1 Materials

All solvents and chemical materials for syntheses were purchased from commercial sources and used as received without further purification.

1.2 Instrumentation.

The powder XRD diffractograms were obtained on a Bruker AXS D8 Advance diffractometer instrument with Cu Kα radiation (λ = 1.54056 Å) in the angular range 2θ = 5-50° at 293 K. The Fourier transform infrared (FT-IR) spectrum was recorded with KBr pellets in the range 4000-400 cm^{-1} with a Nicolet AVATAR FT-IR 360 spectrometer. Scanning electron microscopy (SEM) measurements were carried out on a NOVA Nano SEM 450 field emission SEM under an accelerating voltage of 500 V. Fe element valence was analyzed on an X-ray photoelectron spectroscopy (XPS) spectrum. The thermogravimetric analysis (TGA) was carried out at a ramp rate of 10 °C/min in a nitrogen flow with a Mettler-Toledo TGA/ SDTA851 instrument. Liquid UV-vis spectra were performed on a TU-1900 spectrophotometer. Solid UV-vis spectra were recorded on Hitachi U-4100 UV-vis-NIR spectrophotometer. Fluorescent spectra were recorded on Edinburgh FLS 920 stable/transient fluorescence spectrometer. ^{1}H NMR spectra were recorded on a Bruker Avance II 400 type spectrometer with (tetramethylsilane) TMS as internal standard. A CHI660 electrochemical workstation was used for the cyclic voltammetry measurements (CV). The generated product were characterized on an Agilent 8860 GC systems using a 5% phenyl-95% methyl polysiloxane column (30 m × 0.32 mm × 0.5µm) and a flame ionization detector (FID), and nitrogen was used as a carrier gas. The EPR spectra were performed on BRUKER E500. The light source is a 395 nm lamp.

1.3 Substrate Encapsulation Experiments of Fe_3-MOF.

Firstly, **Fe_3-MOF** was cleaned three times ultrasonic with ethanol and water. The **Fe_3-MOF** was soaked in ethanol for three days and the ethanol was changed every 12 hours. Then **Fe_3-MOF** was dried in a vacuum oven at 100 ℃ for 12 hours. **Fe_3-MOF** was then immersed in acetonitrile solution containing the substrate for 24h. The soaked **Fe_3-MOF** was washed three times with fresh acetonitrile solution to remove the substrate from the surface. The substrate-loaded crystals were directly used for IR, and digested with DMSO-d_6/DCl for ^{1}H NMR.

1.4 General Procedure for Photocatalytic C-N Bond Formation (HAT).

In a standard reaction, ditertbutyl azodicarboxylate (DBAD) (0.2 mmol), C(*sp*^{3})-H bonds substrate (2mmol), pyridine hydrochloride (4.5 mol%) and **Fe_3-MOF** (0.5 mol %) were mixed in acetonitrile (2 mL) in a 16 mL quartz tube. The resulting mixture was stirred and irradiated with a 30w 395 nm LED under argon atmosphere at room temperature for 2-5 hours. After the reaction, the mixture was centrifuged at 8000 rpm for 3 min, and the supernatant was concentrated under vacuum distillation. The residues were separated by a silica gel column (EtOAc/petroleum ether) to obtain the isolated yields.

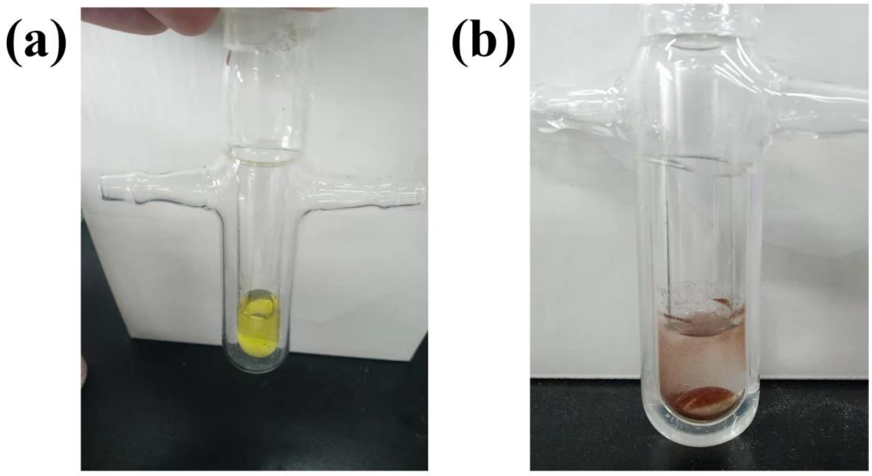

Figure S1. The detailed pictures of the color before (a) and after (b) the reaction.

1.5 General Procedure for Photocatalytic C-C Bond Formation (HAT).

In a standard reaction, benzylidenemalononitrile (0.2 mmol), C(sp^3)-H bonds substrate (2 mmol), pyridine hydrochloride (4.5 mol%) and **Fe_3-MOF** (0.5 mol %) were mixed in acetonitrile (2 mL) in a 16 mL quartz tube. The resulting mixture was stirred and irradiated with a 30w 395 nm LED under argon atmosphere at room temperature for 3-12 hours. After the reaction, the mixture was centrifuged at 8000 rpm for 3 min, and the supernatant was concentrated under vacuum distillation. The residues were separated by a silica gel column (EtOAc/petroleum ether) to obtain the isolated yields.

1.6 General Procedure for Photocatalytic C-B Bond Formation (HAT).

In a standard reaction, a 20 mL oven-dried round-bottom Schlenk bottle equipped with a magnetic stir bar, $B_2(cat)_2$ (0.2 mmol, 1 equiv.), C(sp^3)-H bonds substrate (2 mmol, 10 equiv.), **Fe_3-MOF** (20 mmol %) and pyridine hydrochloride (20 mmol %) were added CH_3CN (2 mL) were then added under argon atmosphere. The resulting mixture was sealed and then subjected to freeze-pump-thaw for three times. After that, the resulting mixture was stirred in argon atmosphere under a 10 W 395nm LED and irradiated for 16 hours. After the reaction, pinacol (0.2 mmol) and triethylamine (0.3 ml) in dichloromethane (1ml) were added, the solvent was continued stirring for 1 h. Finally, the mixture was removed under reduced pressure and the residue was purified by flash column chromatography (EtOAc/petroleum ether) on silica gel to obtain the isolated yields.

1.7 General Procedure for Photocatalytic C-C Bond Formation (oxidation).

In a standard reaction, heteroarene (0.2 mmol), C(sp^3)-H bonds substrate (2 mmol, 10 equiv.), **Fe_3-MOF** (0.5 mmol %) and pyridine hydrochloride (4.5 mmol %) were mixed in dry DCM (2 mL) in a 16 mL quartz tube. The resulting mixture was stirred and irradiated with a 30w 395 nm LED under O_2 atmosphere for 6-12 hours. After the reaction, the solvent was removed under vacuum. The residue was purified by column chromatography on silica gel using EtOAc/petroleum ether as eluent to give products.

1.8 General Procedure for Photocatalytic Functionalization of Gaseous Alkanes.

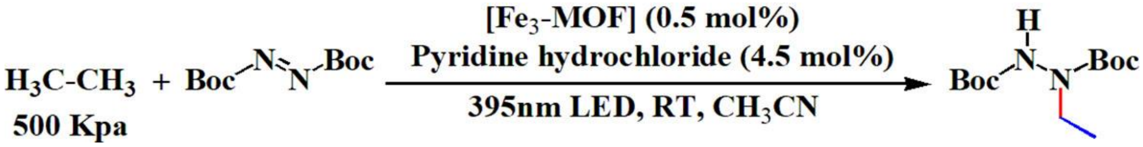

Reaction: DBAD (0.2 mmol), **Fe$_3$-MOF** (0.5 mol%), pyridine hydrochloride (4.5 mol%) were mixed in acetonitrile (4 mL) in the high pressure resistant reactor. The reactor was pressurized to 500 kPa of ethane under irradiation with a 30w 395 nm LED within 2 h. After the reaction, the mixture was centrifuged at 8000 rpm for 3 min, and the supernatant was concentrated under vacuum distillation. The residues were separated by a silica gel column (EtOAc/petroleum ether) to obtain the isolated yields.

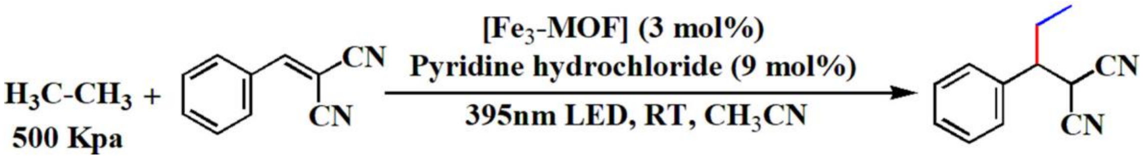

Reaction condition: benzylidenemalononitrile (0.2 mmol), **Fe$_3$-MOF** (3 mol%), pyridine hydrochloride (9 mol%) were mixed in acetonitrile (4 mL) in the high pressure resistant reactor. The reactor was pressurized to 500 kPa of ethane under irradiation with a 395 nm LED within 10 h. After the reaction, the mixture was centrifuged at 8000 rpm for 3 min, and the supernatant was concentrated under vacuum distillation. The residues were separated by a silica gel column (EtOAc/petroleum ether) to obtain the isolated yields.

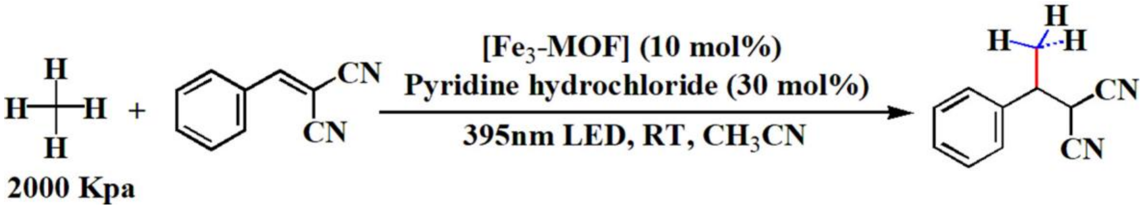

Reaction condition: benzylidenemalononitrile (0.2 mmol), **Fe$_3$-MOF** (10 mol%), pyridine hydrochloride (30 mol%) were mixed in acetonitrile (4 mL) in the high pressure resistant reactor. The reactor was pressurized to 2000 kPa of methane under irradiation with a 395 nm LED within 16 h. After the reaction, the mixture was centrifuged at 8000 rpm for 3 min, and the supernatant was concentrated under vacuum distillation. The residues were separated by a silica gel column (EtOAc/petroleum ether) to obtain the isolated yields.

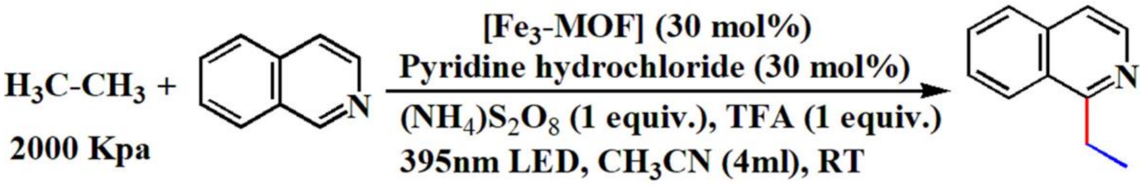

Reaction condition: isoquinoline (0.2 mmol), **Fe$_3$-MOF** (30 mol%), pyridine hydrochloride (30 mol%), $(NH_4)S_2O_8$ (0.2 mmol) and trifluoroacetic acid (TFA) (0.2 mmol) were mixed in acetonitrile (4 mL) in the high pressure resistant reactor. The reactor was pressurized to 2000 kPa of ethane under irradiation with a 395 nm LED within 24 h. After the reaction, the mixture was centrifuged at 8000 rpm for 3 min, and the supernatant was

concentrated under vacuum distillation. The residues were separated by a silica gel column (EtOAc/petroleum ether and 1 v% TEA) to obtain the isolated yields.

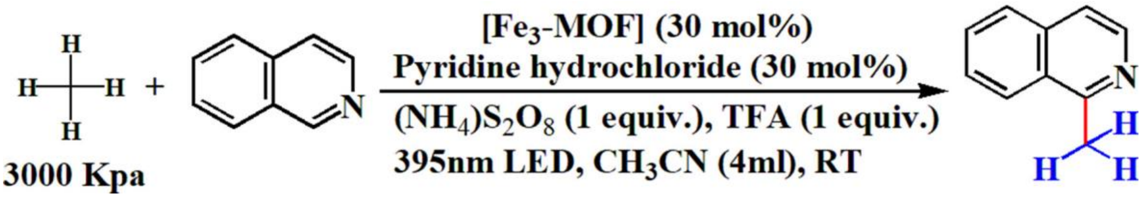

Reaction condition: isoquinoline (0.2 mmol), **Fe_3-MOF** (30 mol%), pyridine hydrochloride (30 mol%), $(NH_4)S_2O_8$ (0.2 mmol) and trifluoroacetic acid (TFA) (0.2 mmol) were mixed in acetonitrile (4 mL) in the high pressure resistant reactor. The reactor was pressurized to 3000 kPa of methane under irradiation with a 395 nm LED within 72 h. After the reaction, the mixture was centrifuged at 8000 rpm for 3 min, and the supernatant was concentrated under vacuum distillation. The residues were separated by a silica gel column (EtOAc/petroleum ether and 1 v% TEA) to obtain the isolated yields.

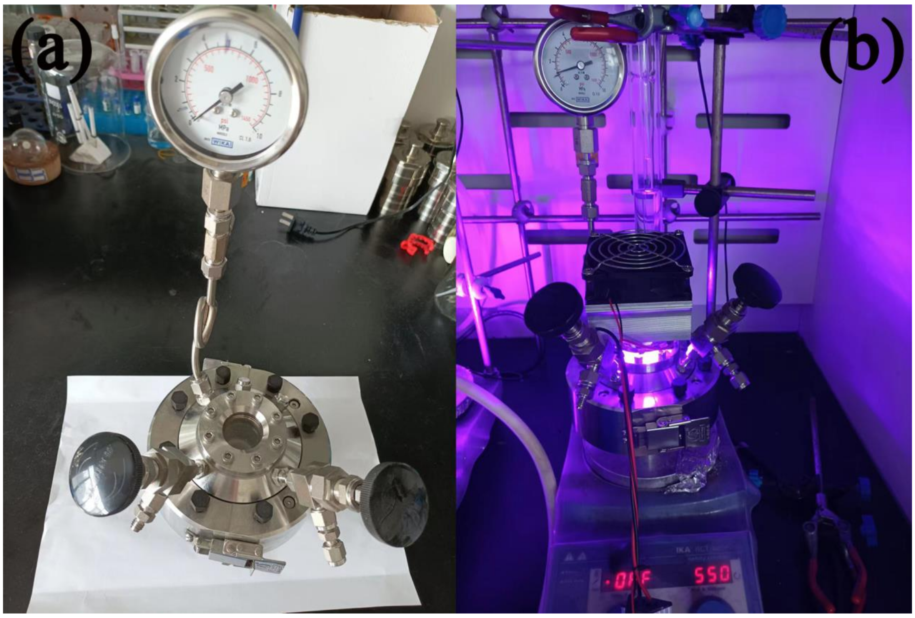

Figure S2. The detailed picture of the functionalization of gasous alkane in high pressure resistant reactor under 395 nm LED.

1.9 General Procedure for Photocatalytic C=O Bond Formation (oxidation).

In a standard reaction, C(*sp*^{3})-H bonds substrate (0.2 mmol), pyridine hydrochloride (4.5 mol%) and **Fe_3-MOF** (0.5 mol %) were mixed in acetonitrile (2 mL) in a 16 mL quartz tube. The resulting mixture was stirred and irradiated with a 395 nm LED under O_2 atmosphere at room temperature for 3 to 7 hours. After the reaction, the mixture was centrifuged at 8000 rpm for 3 min, and the supernatant was concentrated under vacuum distillation. The residues were separated by a silica gel column (EtOAc/petroleum ether) to obtain the isolated yields.

1.10 General procedure for oxidation of thioxanthene by Fe_3-MOF.

In a standard reaction, thioxanthene (0.05 mmol), catalyst (0.5 mol %) and pyridine hydrochloride (4.5 mol%) were mixed in acetonitrile (2 mL) in a 16 mL quartz tube. The resulting mixture was stirred and irradiated with a 395 nm LED under O_2 atmosphere at room temperature for 1 to 5 h. After the reaction, the reaction liquid was analyzed by ^1H NMR spectra.

2. Preparation of Fe_3-MOF.

2.1 4,4′,4″-Tricarboxytriphenylamine (H_3TCA) was prepared according to the literature methods.[1]

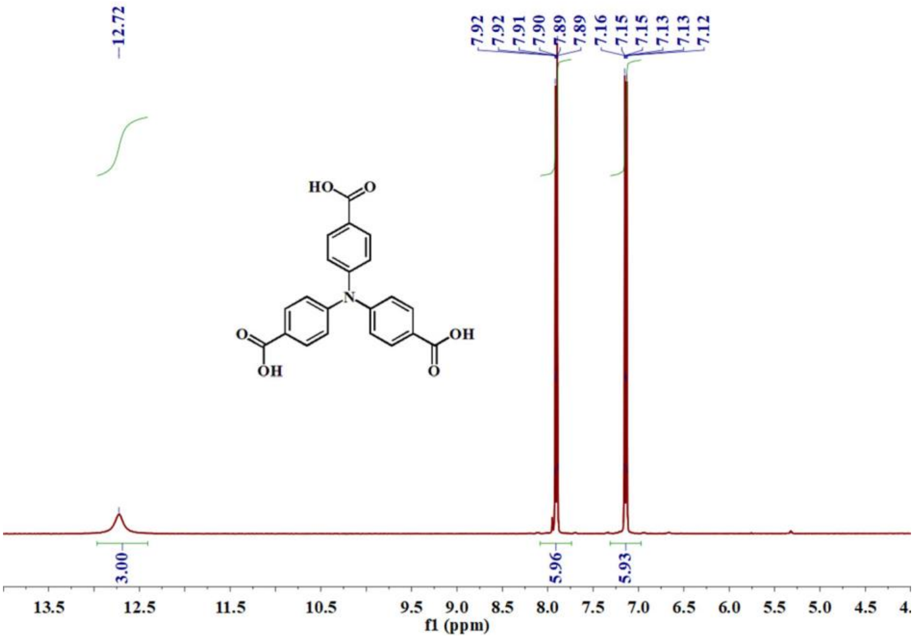

Figure S3. ^{1}H NMR spectra of the 4,4',4''-Tricarboxytriphenylamine (TCA).

^{1}H NMR (400 MHz, DMSO) δ 12.72 (s, 3H), 8.08 - 7.74 (m, 6H), 7.31 - 6.97 (m, 6H).

2.2 Synthesis of preformed Fe_3 cluster. $Fe_3(\mu_3\text{-}O)(CH_3COO)_6(H_2O)_3$ is prepared according to reported procedure.[2] In detail, an aqueous solution (35 ml) of sodium acetate trihydrate (42 g, 0.31 mol) is added to the stirred iron nitrate nonahydrate (8 g, 0.02 mol) aqueous solution (35 ml). The resulting red precipitate is filtered, washed with ethanol, and dried in air.

2.3 Synthesis of Fe_3-MOF ($Fe^{III}_6[TCA^{3-}]_4$•$6CH_3COO^-$). A mixture of Fe_3 cluster (15 mg), ligand TCA (15 mg), acetic acid (0.2 ml) and 3 ml N-Methylformamide (NMF). The resulting solution was transferred to a Teflon lined autoclave and kept under autogenous pressure at 140 ºC for 48 h. After slow cooling to room temperature at a rate of 10 °C/h, red black crystals were collected by centrifugation and fully washed with NMF and ethanol. The crystals were soaked in 20 ml of ethanol for 2 days replacing the solvent, then soaked in 20 ml water for 2 days replacing the solvent. Finally, filtered and dried under vacuum. Yield: 35%.

3. Single Crystal X-ray Crystallography.

Single crystal X-ray diffraction data collection for compound **Fe_3-MOF** was performed using a Bruker Smart Apex CCD diffractometer with Mo-K*a* (λ = 0.71073 Å) radiation at 173 K. The data were acquired using the SMART and SAINT programs. The structure was solved by direct methods and refined by full matrix least-squares methods by the program SHELXL-2018.

In the structural refinement of **Fe_3-MOF**, anisotropic displacement parameters were used to refine all non-hydrogen atoms. The hydrogen atoms within the ligand backbones were fixed geometrically at calculated distances and allowed to ride on the parent non-hydrogen atoms. Due to some free molecules are highly disordered, it is impossible to accurately determine the positions of all the atoms contained in these free molecules. So the SQUEEZE subroutine in PLATON was used. In order to balance the charge of **Fe_3-MOF**, six acetate ions should be added.

Table S1. Crystal parameters and structure refinement dates for **Fe_3-MOF**.

Compound	**Fe_3-MOF**
Formula	$C_{84}H_{60}Fe_6N_4O_{32}$
M	1972.46
Crystal system	orthorhombic
Space group	P c a 21
a/Å	30.652(5)
b/Å	15.895(3)
c/Å	40.287(8)
α/deg	90
β/deg	90
γ/deg	90
V/Å^3	19628(6)
Z	4
D_{calcd}/g cm^{-3}	0.667
T/K	173(2)
Refl.Unique	28036
R_{int}	0.0860
GoF on F^2	0.873
$R1$ / $wR2$ [I >2 $\sigma(I)$]$^{a,\,b}$	0.0559 / 0.1227
CCDC Number	2303072

$^aR_1 = \sum\|F_o|-|F_c\|/\sum|F_o|$, $^bwR_2 = \{\sum[w(F_o^2-F_c^2)^2]/\sum[w(F_o^2)^2]\}^{1/2}$

Table S2. The values of bond valence sum (BVS) for Fe centers of **Fe_3-MOF**.

Compound	atoms	BVS	Oxidation states
**Fe_3-MOF**	Fe1	3.26	III
	Fe2	3.18	III
	Fe3	3.48	III
	Fe4	3.21	III
	Fe5	3.03	III
	Fe6	3.17	III

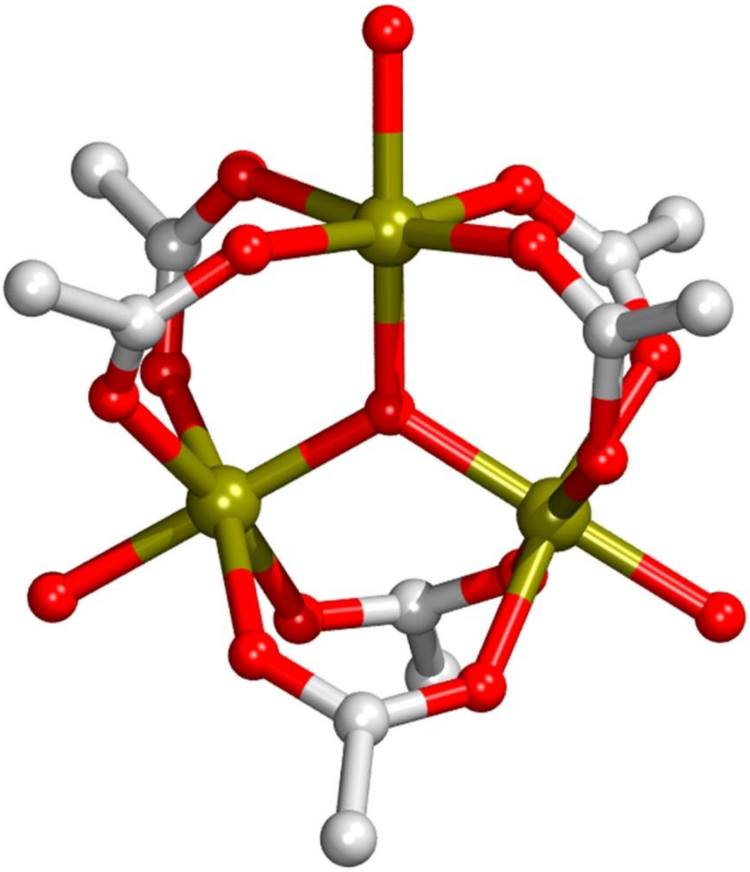

Figure S4. Structure of the Fe_3 cluster in **Fe_3-MOF**.

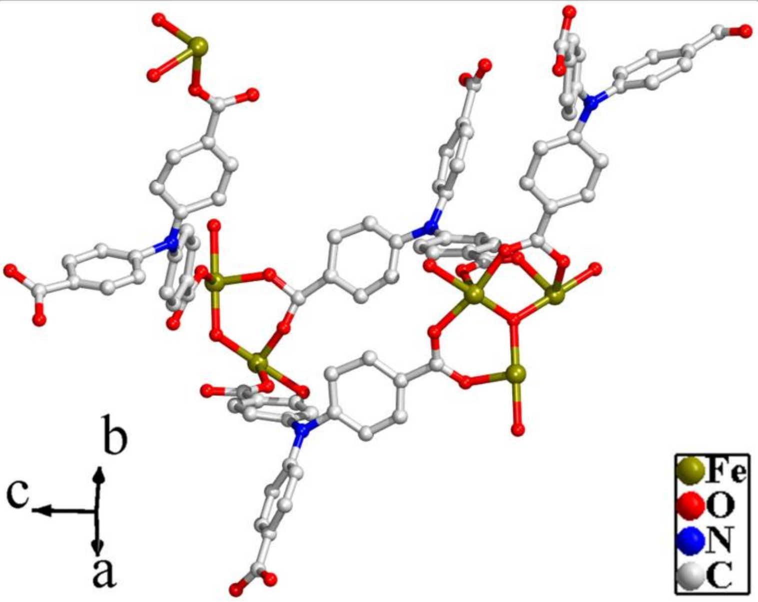

Figure S5. View of the asymmetric unit of **Fe_3-MOF**. All hydrogen atoms and free water molecules are omitted for clarity.

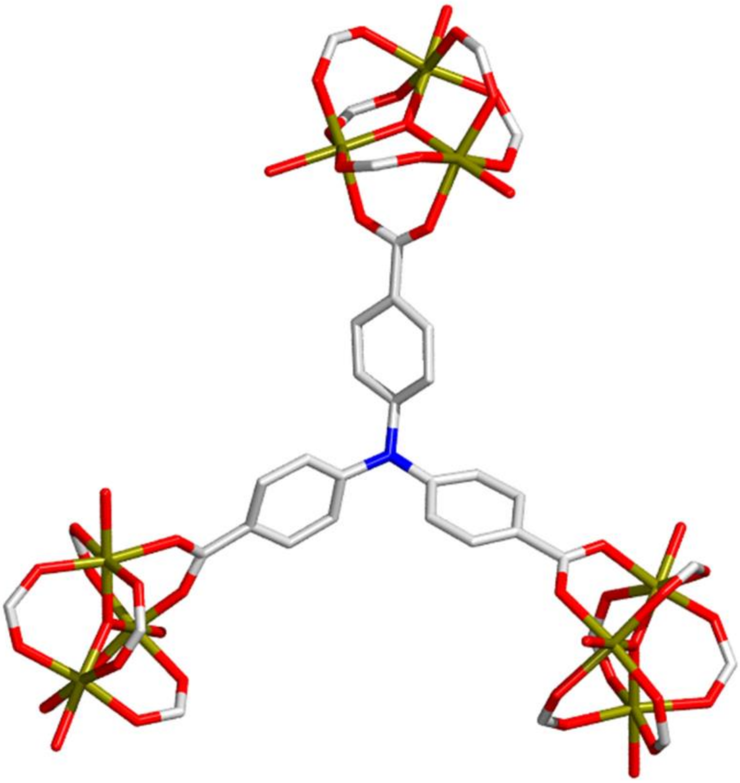

Figure S6. TCA coordinated by three Fe_3 clusters in **Fe_3-MOF**.

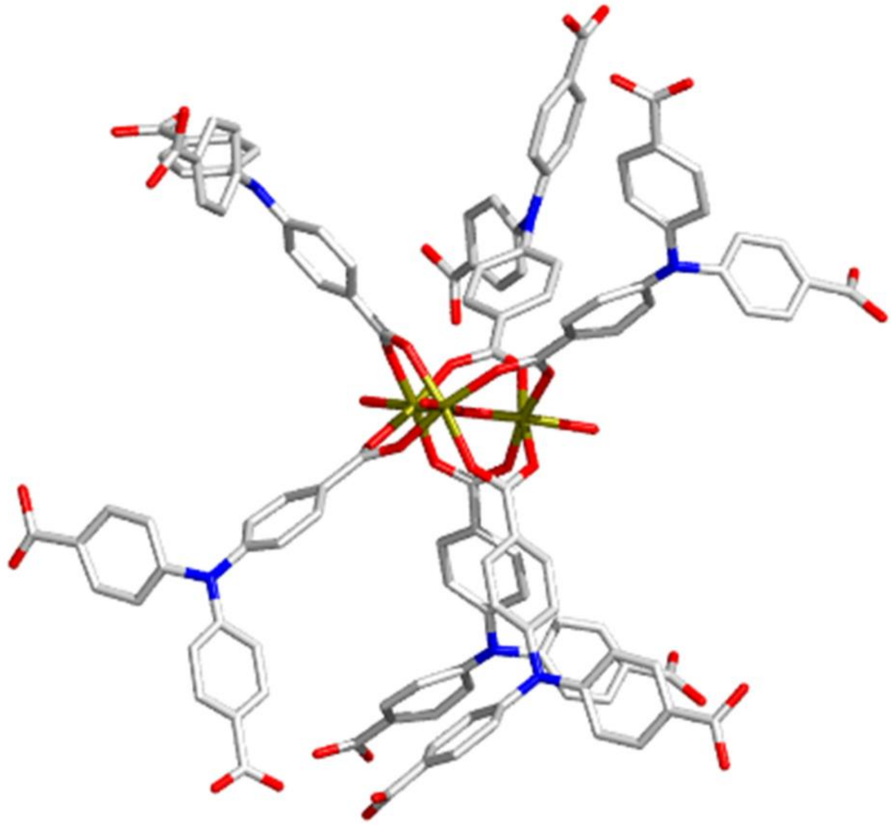

Figure S7. Fe_3 clusters coordinated by six TCA ligands in **Fe_3-MOF**.

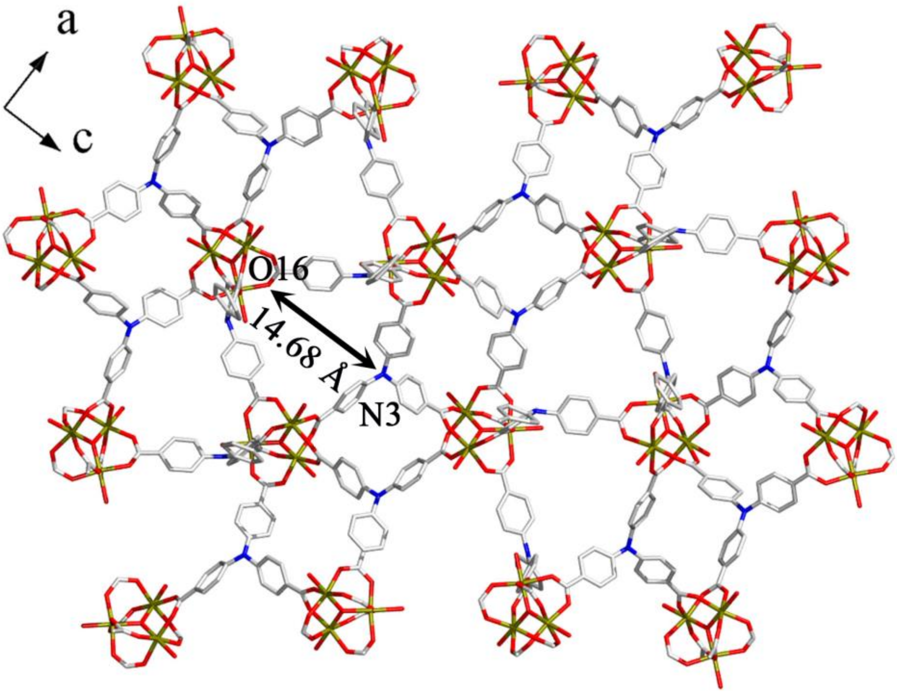

Figure S8. The 3D framework of **Fe_3-MOF** in the b-axis direction.

The maximum window size in **Fe_3-MOF** is 14.68 Å.

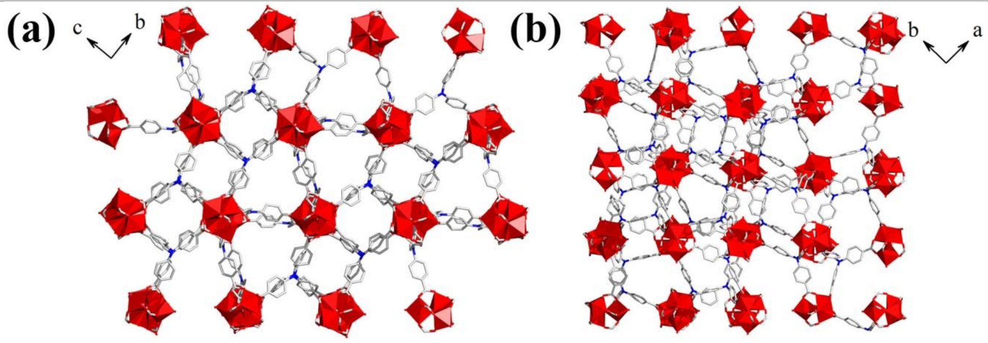

Figure S9. (a) 3D framework of **Fe_3-MOF** in the a-axis direction. (b) 3D framework of **Fe_3-MOF** in the c-axis direction.

4. Characterization of Fe_3-MOF.

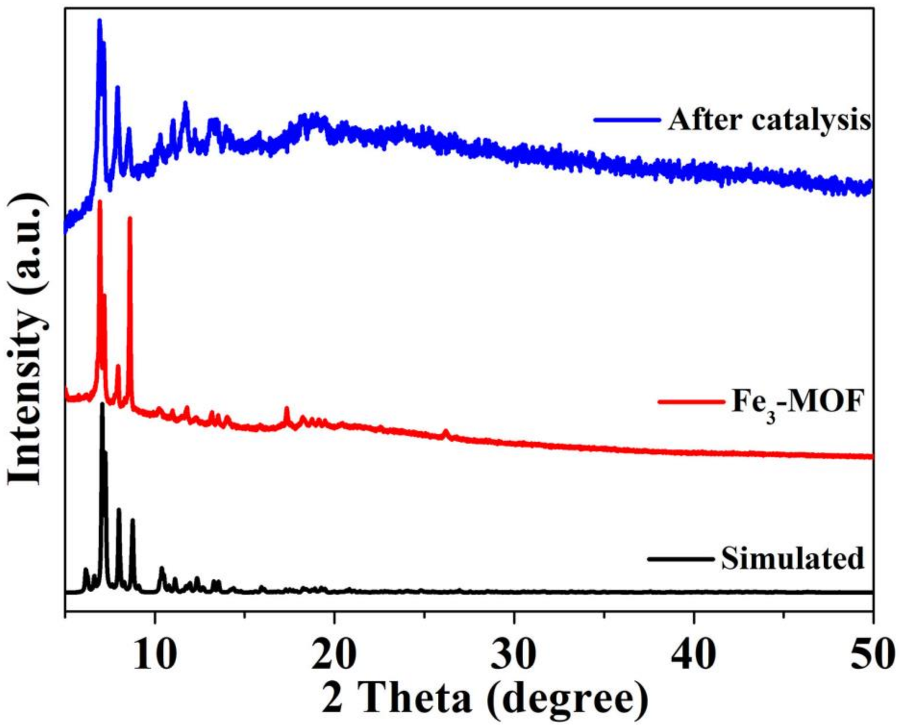

Figure S10. PXRD patterns of the single-crystal simulated **Fe_3-MOF** (black), the fresh as-synthesized **Fe_3-MOF** (red) and the recycled **Fe_3-MOF** after catalysis (blue).

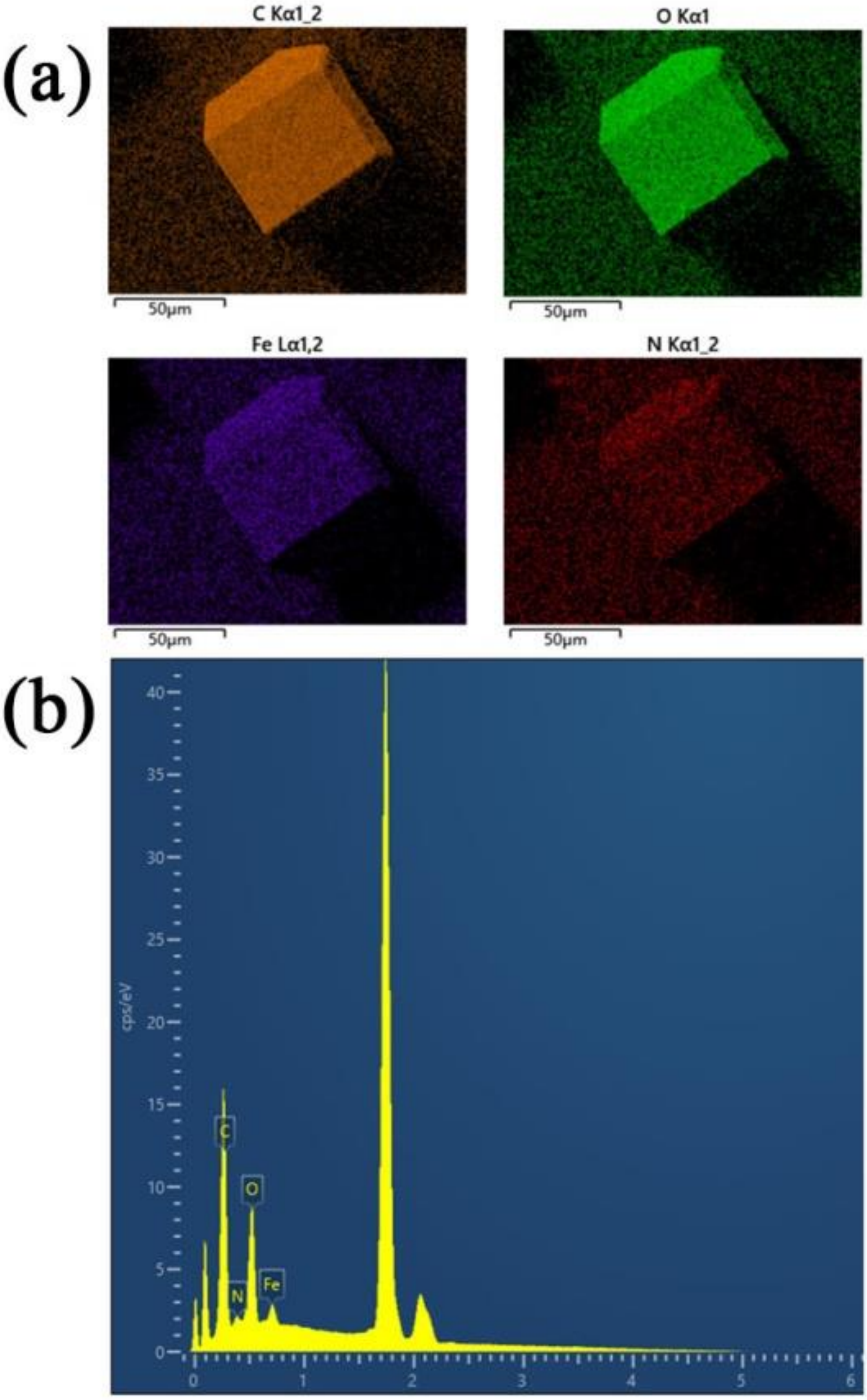

Figure S11. EDS elemental mapping analyses of **Fe_3-MOF**.

Elemental-mapping images show the uniform distributions of Fe, C, N, and O elements in **Fe_3-MOF**.

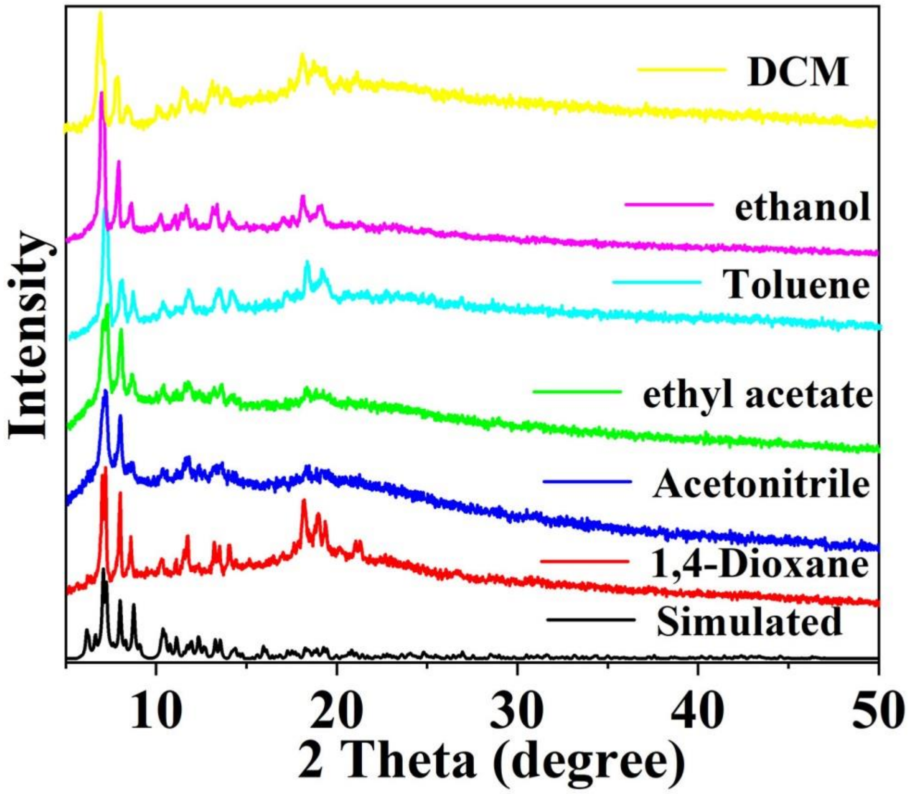

Figure S12. PXRD patterns of **Fe_3-MOF** after being soaked in several common organic solvents.

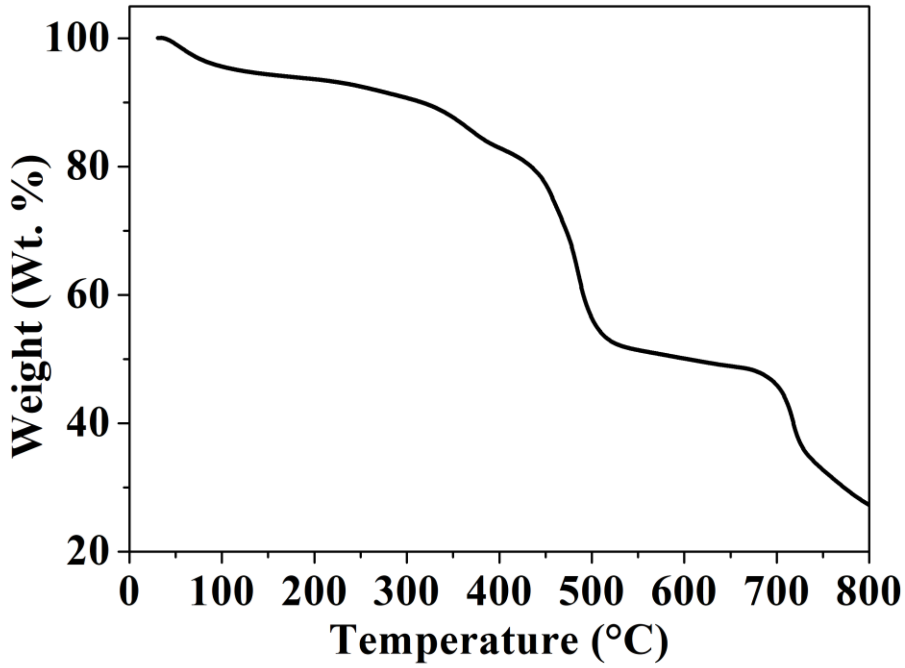

Figure S13. Thermogravimetric analysis of **Fe_3-MOF**.

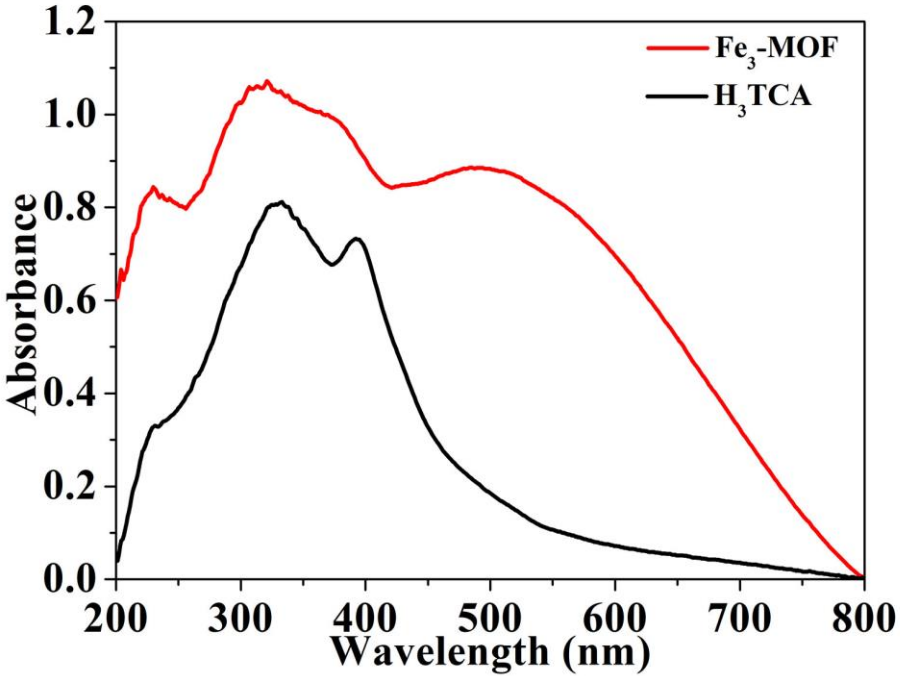

Figure S14. Solid UV-Vis spectrum of H_3TCA (black line) and **Fe_3MOF** (red line).

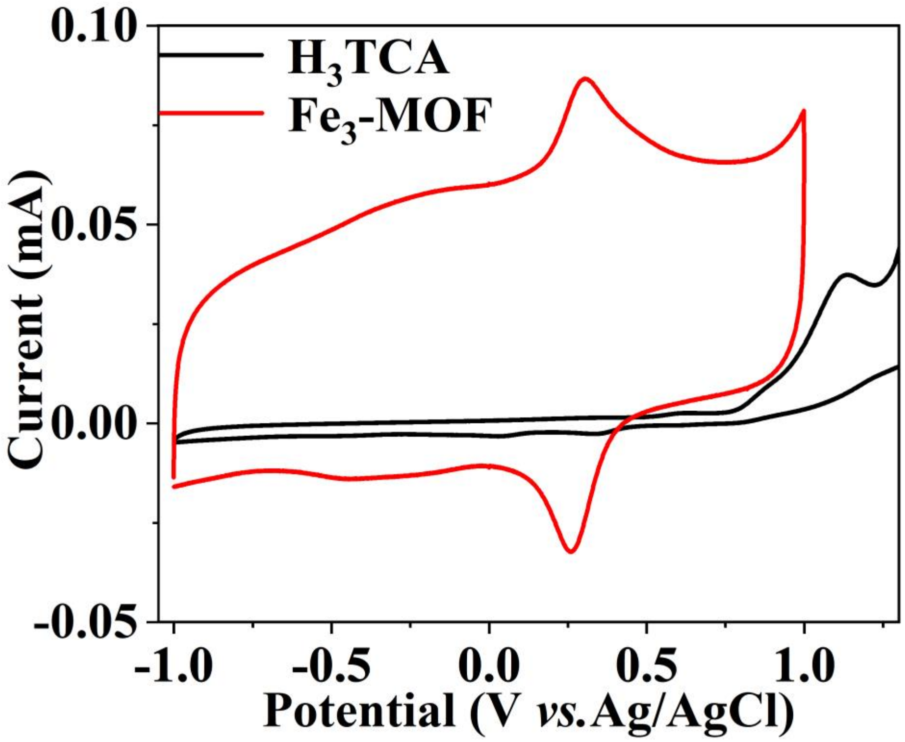

Figure S15. Solid-state cyclic voltammogram of **Fe_3-MOF** and ligand H_3TCA with a scan rate of 0.1 V s^{-1}(*vs.* Ag/AgCl). working electrode: glassy carbon electrode; reference electrode: Ag/AgCl electrode; counter electrode: a platinum-wire; electrolyte: 0.1 M Bu_4NPF_6 (the solvent is DMSO).

The oxidation potential of H_3TCA and **Fe_3-MOF** were 1.131 v and 0.304 v, respectively. Therefore, the oxidation potential of H_3TCA in **Fe_3-MOF** is more positive than that of Fe^{II} species and can oxidize Fe^{II} species to Fe^{III} species during photocatalysis.

5. Substrate Encapsulation Experiments of **Fe_3-MOF**.

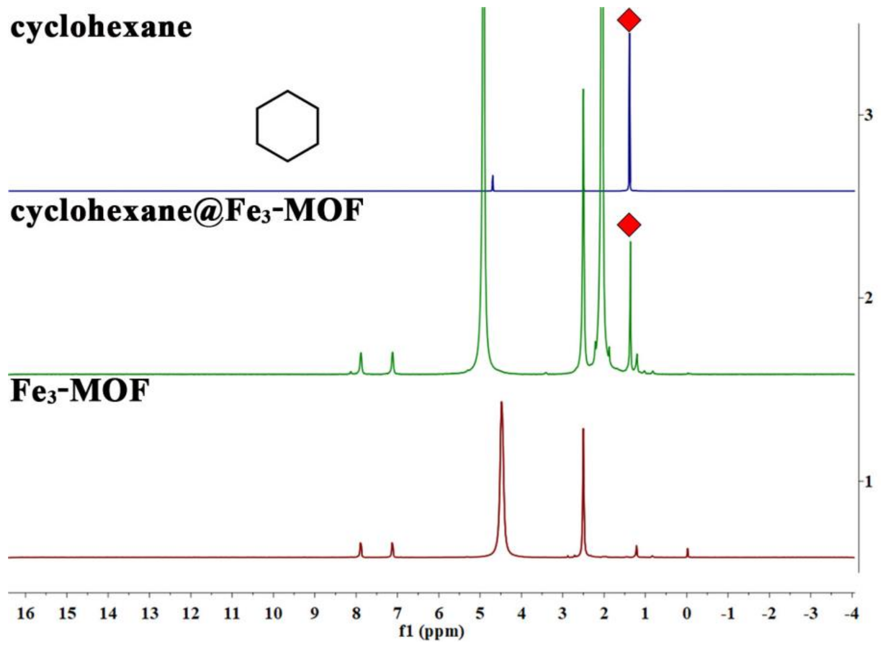

Figure S16. ^{1}H NMR in DMSO-d_6/DCl of cyclohexane, **Fe_3-MOF** impregnated with cyclohexane and **Fe_3-MOF**.

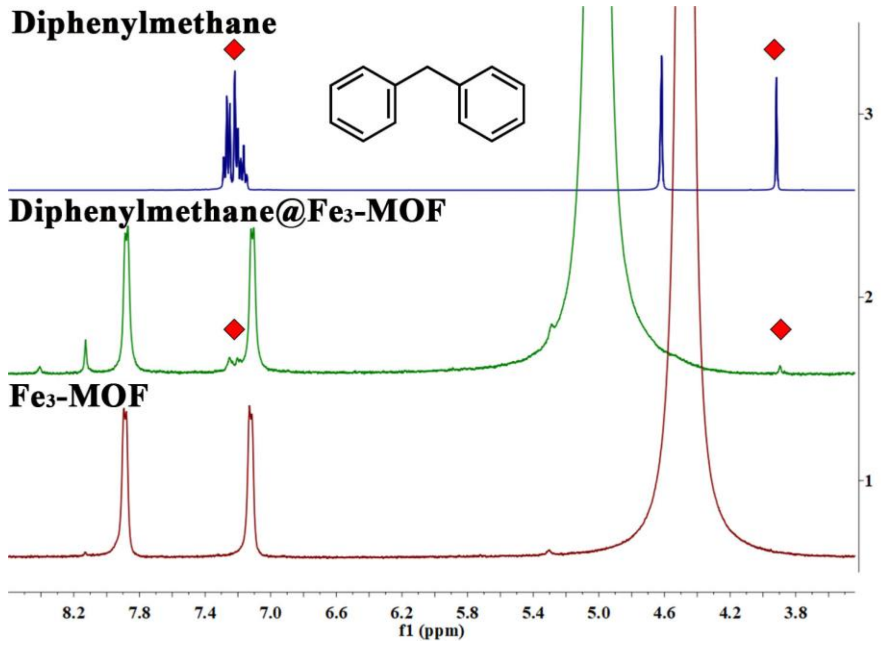

Figure S17. ^{1}H NMR in DMSO-d_6/DCl of diphenylmethane, **Fe_3-MOF** impregnated with diphenylmethane and **Fe_3-MOF**.

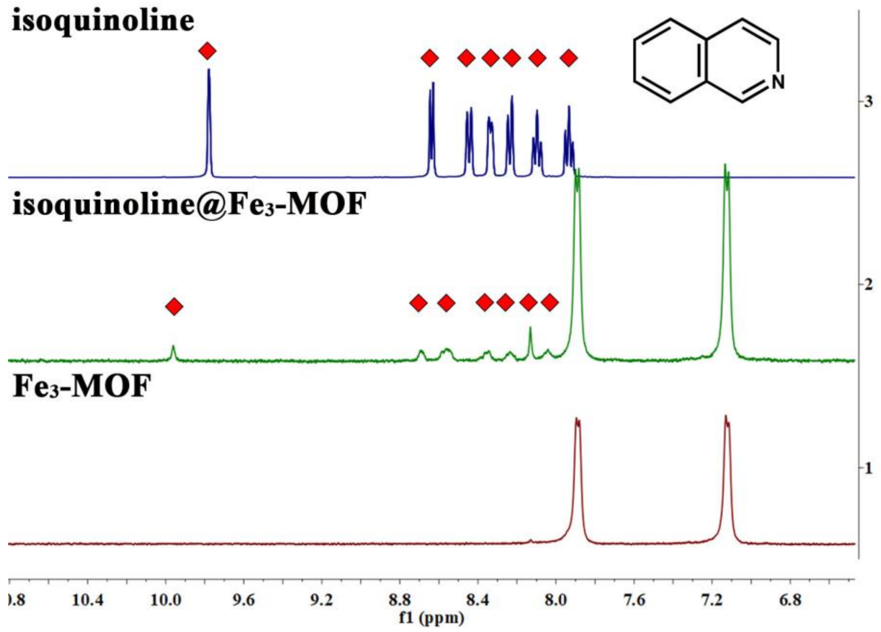

Figure S18. ^{1}H NMR in DMSO-d_6/DCl of isoquinoline, **Fe_3-MOF** impregnated with isoquinoline and **Fe_3-MOF**.

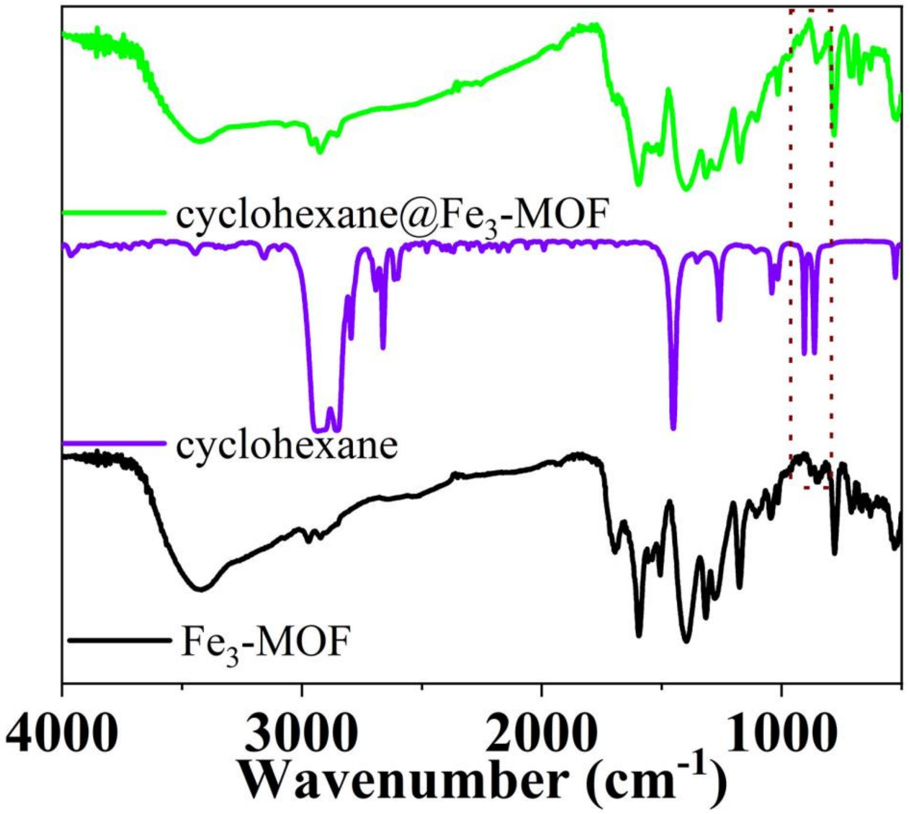

Figure S19. IR spectra of **Fe_3-MOF** impregnated with cyclohexane, cyclohexane, and **Fe_3-MOF**.

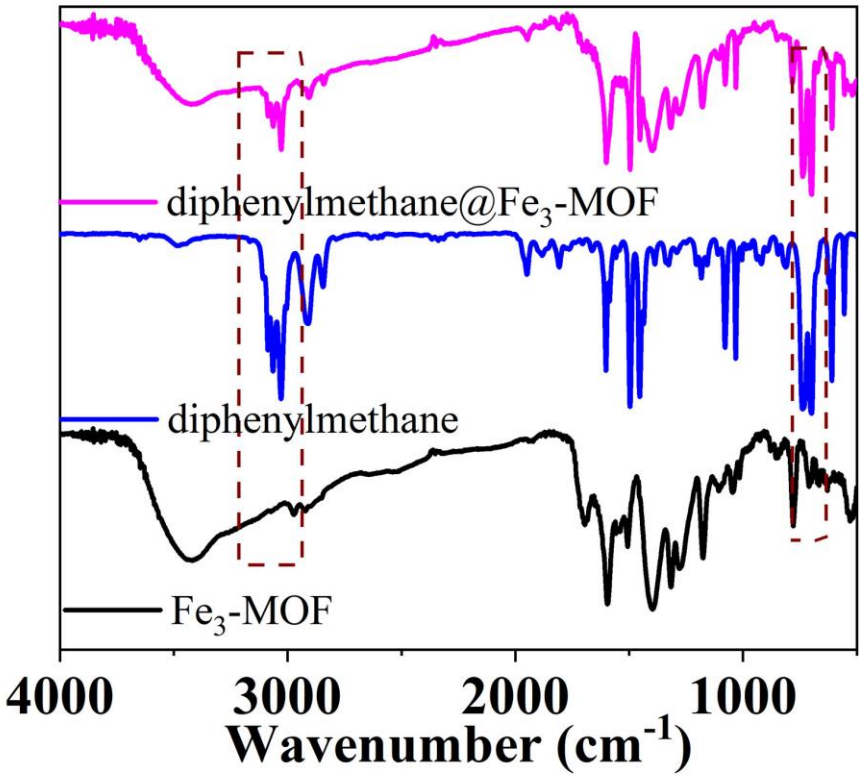

Figure S20. IR spectra of **Fe3-MOF** impregnated with diphenylmethane, diphenylmethane, and **Fe3-MOF**.

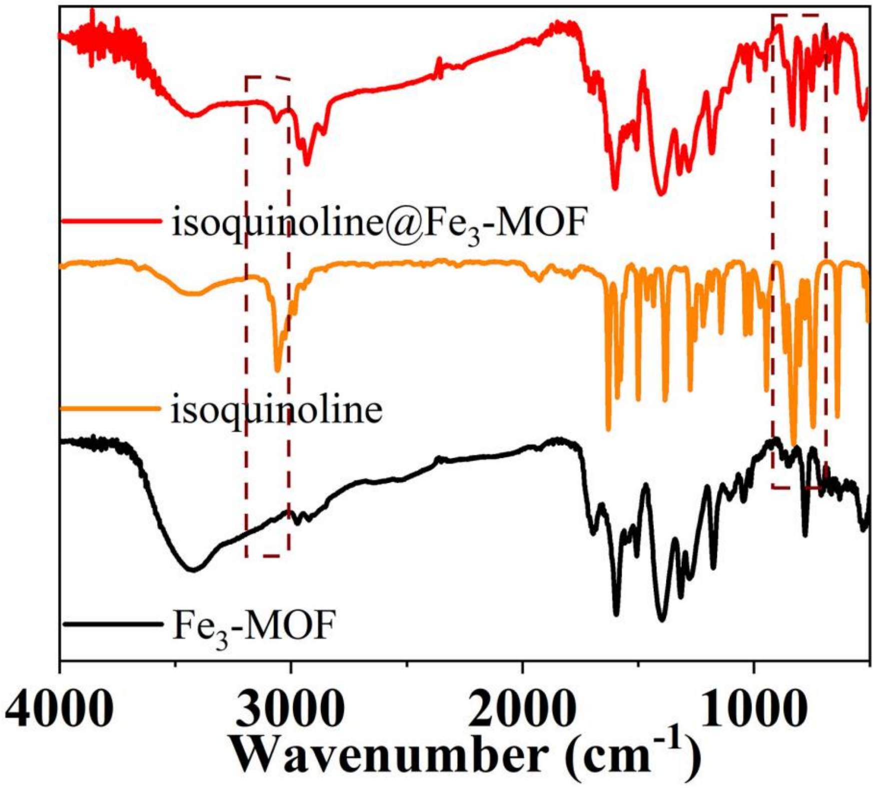

Figure S21. IR spectra of **Fe3-MOF** impregnated with isoquinoline, isoquinoline, and **Fe3-MOF**.

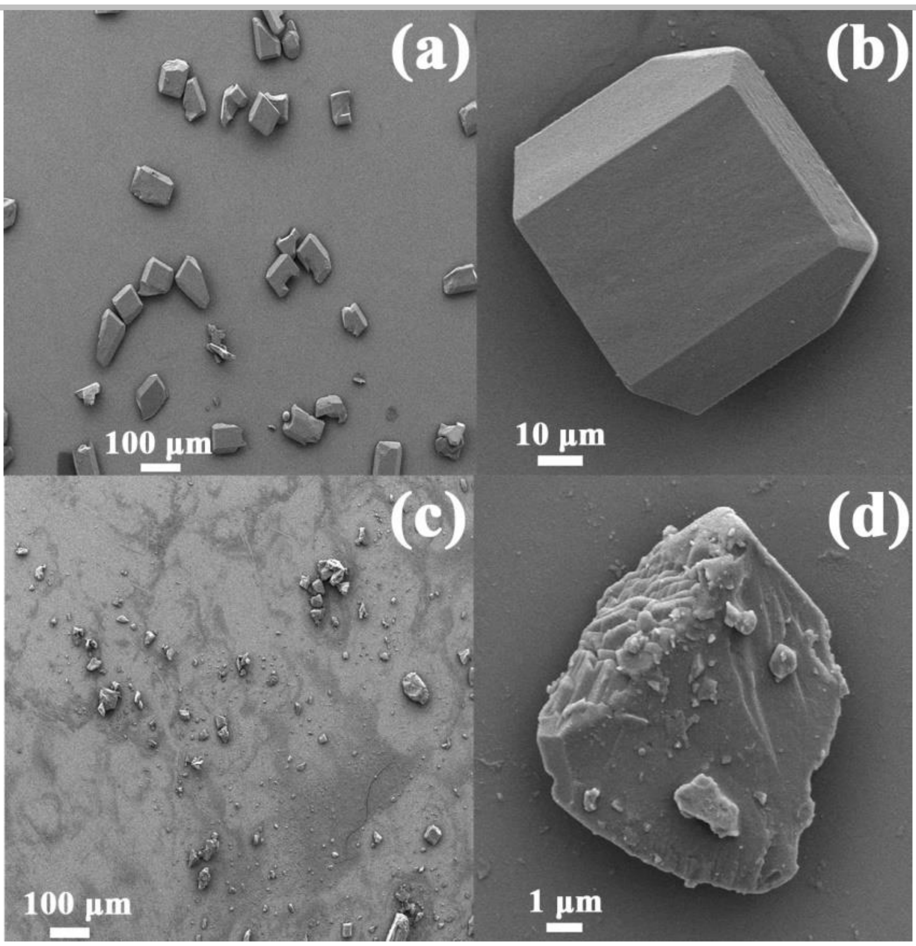

Figure S22. SEM images of the initial crystals (the original synthetic crystal without any treatment) **Fe_3-MOF** (a and b). SEM images of **Fe_3-MOF** after grinding (c and d).

The scanning electron microscope (SEM) showed that the size of **Fe_3-MOF** before and after grinding was 100 µm and 1 µm, respectively, and the yield of C-N coupling (HAT) mode reaction was independent of the catalyst surface area.

6. Characterization of LMCT process between Cl^{-1} ions and **Fe_3-MOF**.

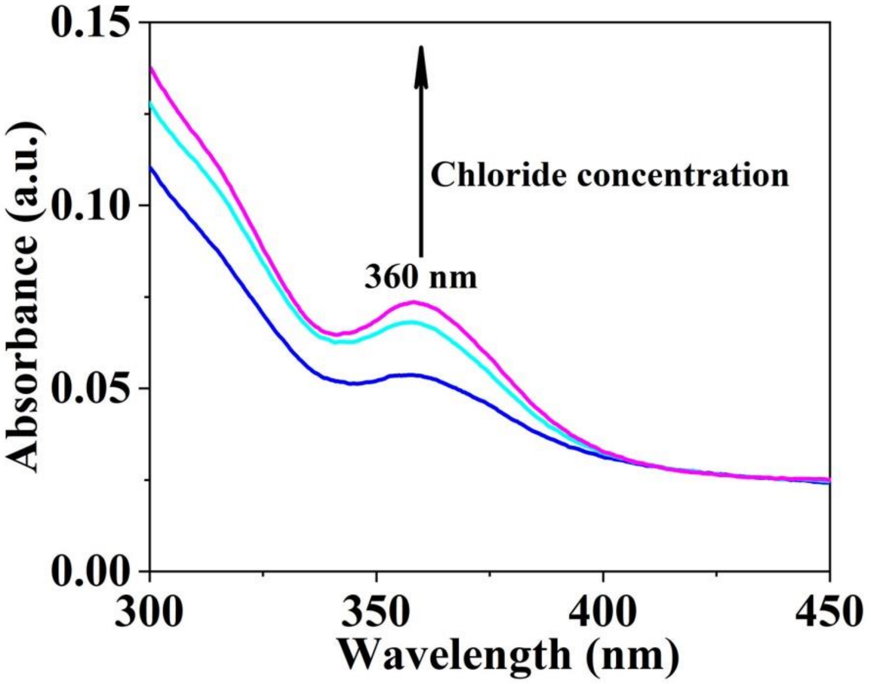

Figure S23. UV-Vis spectra of **Fe_3-MOF** added to acetonitrile solution containing different concentrations of pyridine hydrochloride under light for 3 minutes.

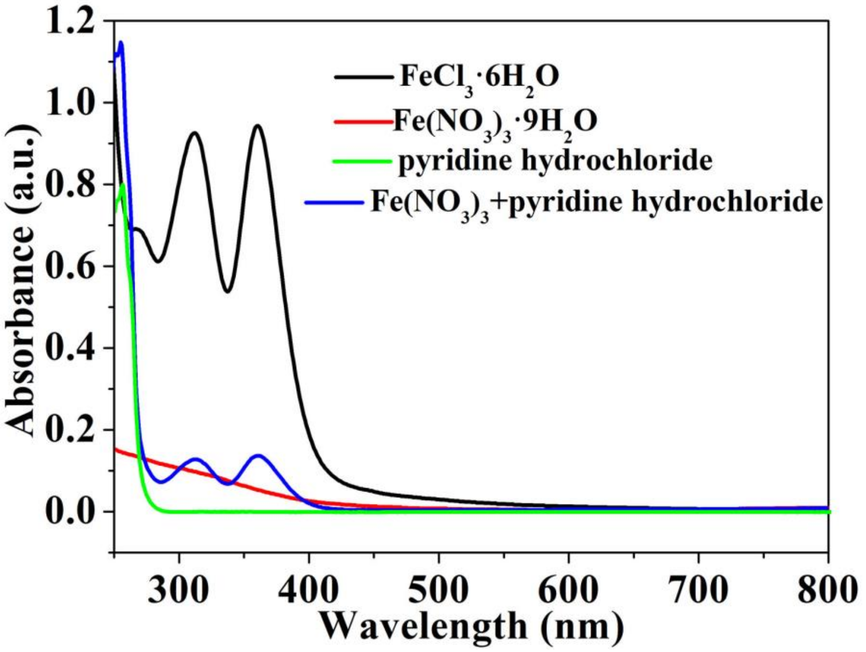

Figure S24. UV-Vis absorption spectra of $FeCl_3·6H_2O$ (1.0×10^{-4} M), $Fe(NO_3)_3·9H_2O$ (0.5×10^{-4} M), pyridine hydrochloride (1×10^{-4} M) and $Fe(NO_3)_3·9H_2O$ + pyridine hydrochloride in CH_3CN under a 395nm LED illumination.

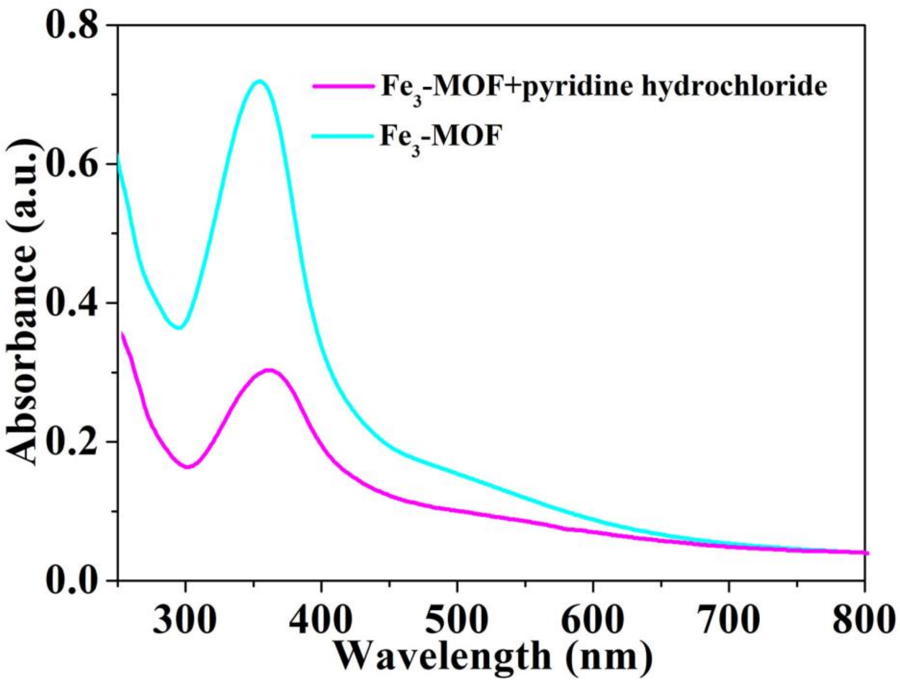

Figure S25. UV-Vis absorption spectra of **Fe3-MOF** (2mg **Fe3-MOF** was immersed in 3mL acetonitrile solution) and **Fe3-MOF** and pyridine hydrochloride without irradiation in CH_3CN.

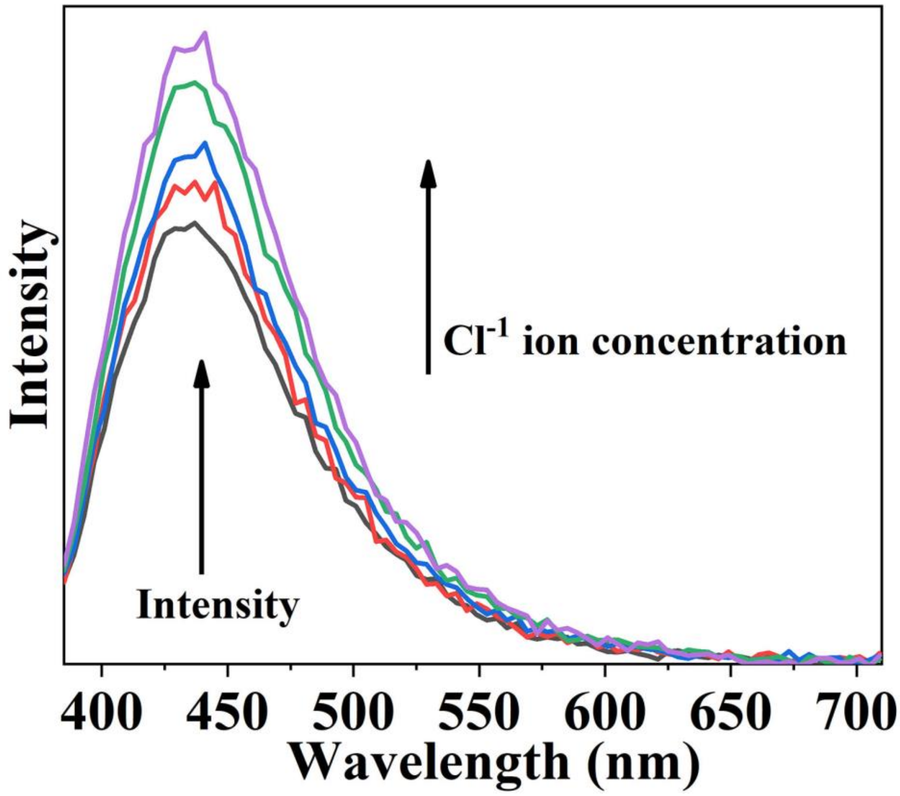

Figure S26. Fluorescence spectra of **Fe3-MOF** with different concentrations of pyridine hydrochloride under N_2 atmosphere.

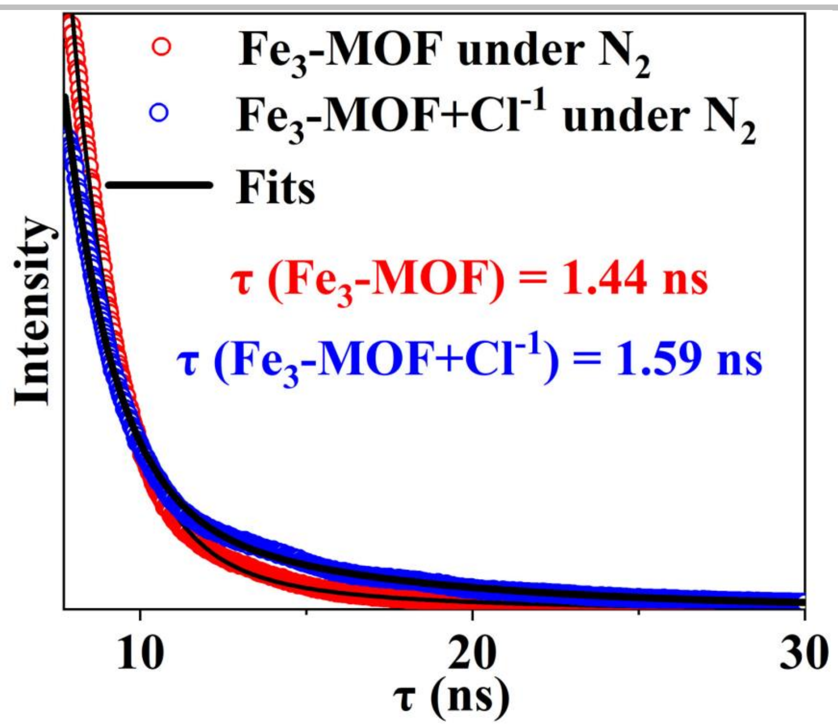

Figure S27. Luminescence decays of **Fe_3-MOF** and Cl^{-1} ions suspensions under N_2 atmosphere.

Radical trapping experiments with TEMPO.

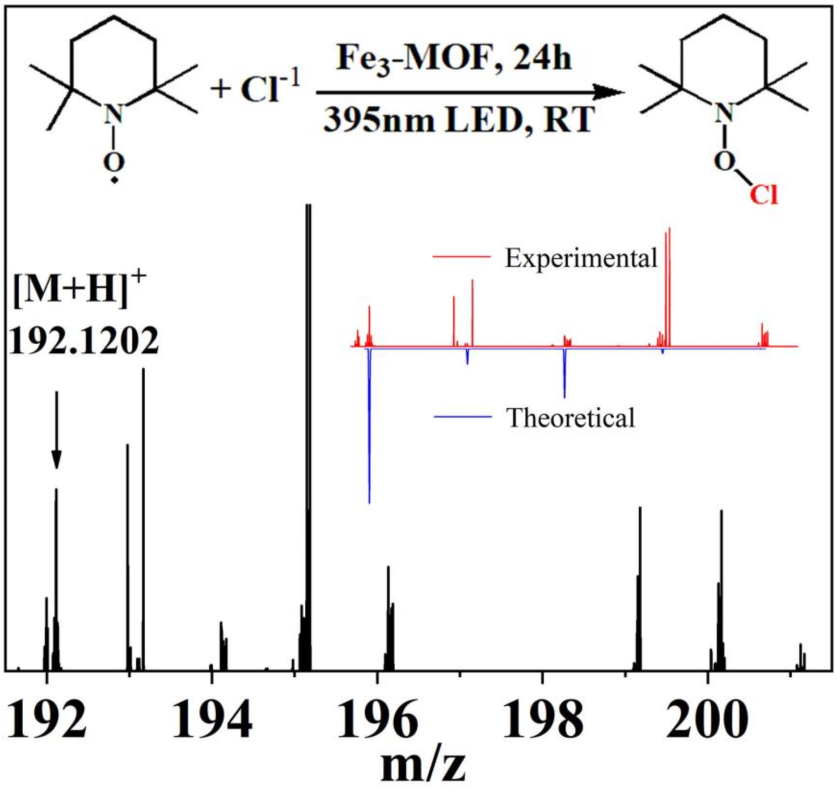

Figure S28. MS for chlorine radical trapping experiment.

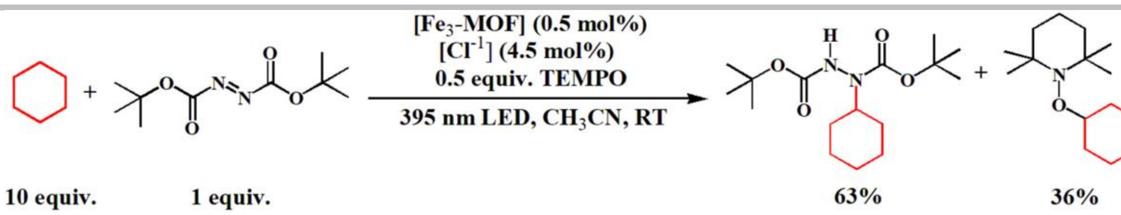

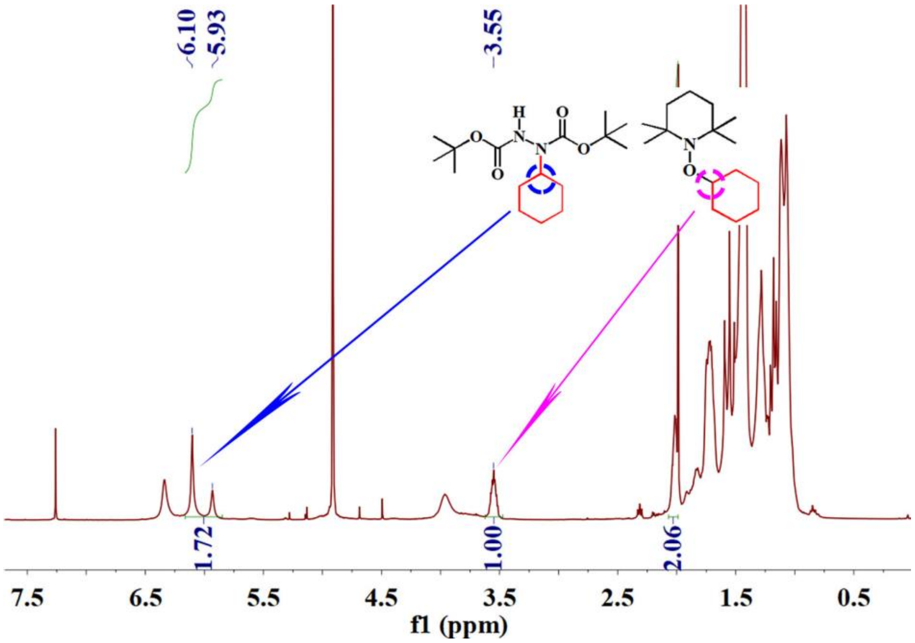

Figure S29. ^{1}H-NMR of crude product of TEMPO traps cyclohexane radical reaction (containing DBAD and TEMPO).

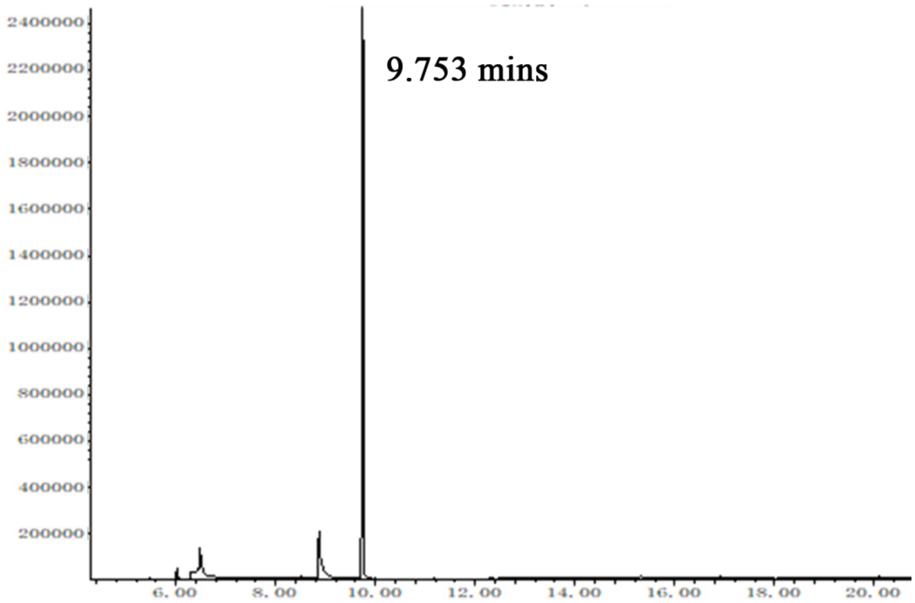

Figure S30. GC spectra of crude product of TEMPO traps cyclohexane radical reaction (containing DBAD and TEMPO).

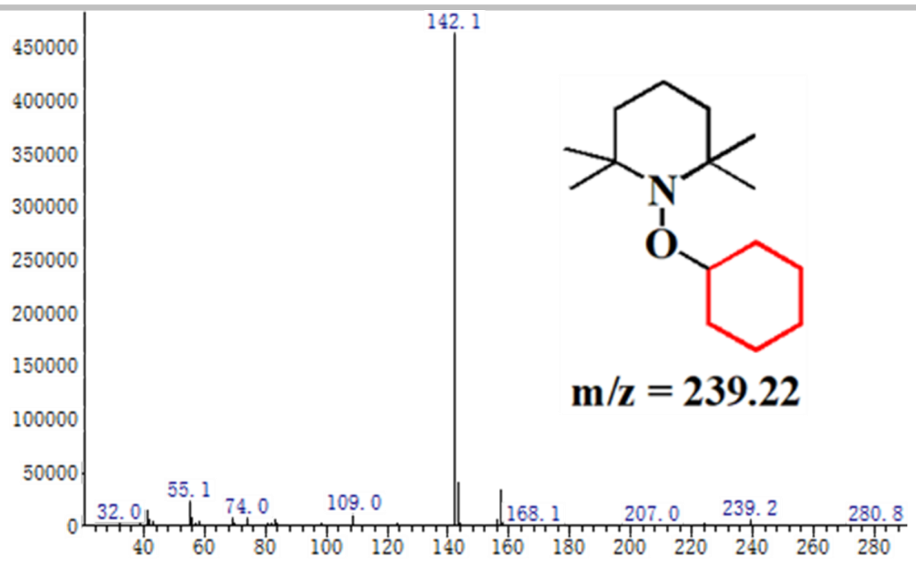

Figure S31. GC-MS spectra of the product with retention time of 9.753 minutes.

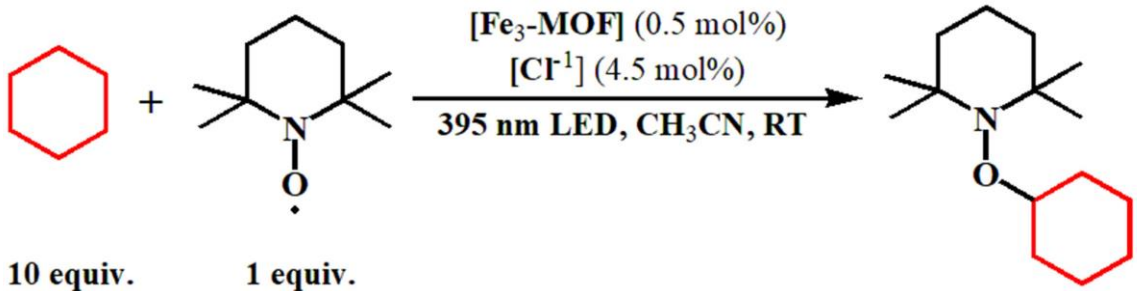

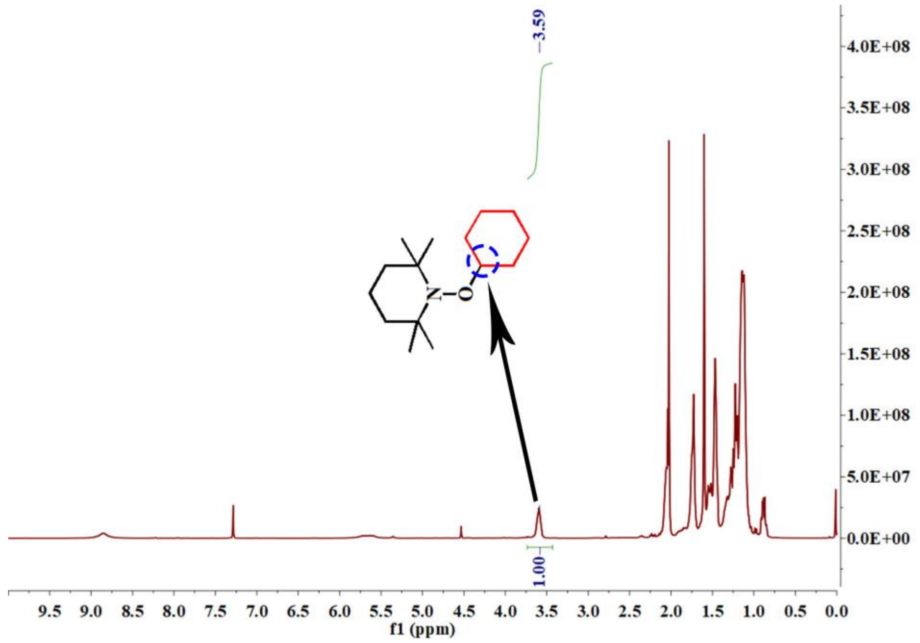

Figure S32. ^{1}H NMR spectra of crude product of TEMPO traps cyclohexane radical reaction.

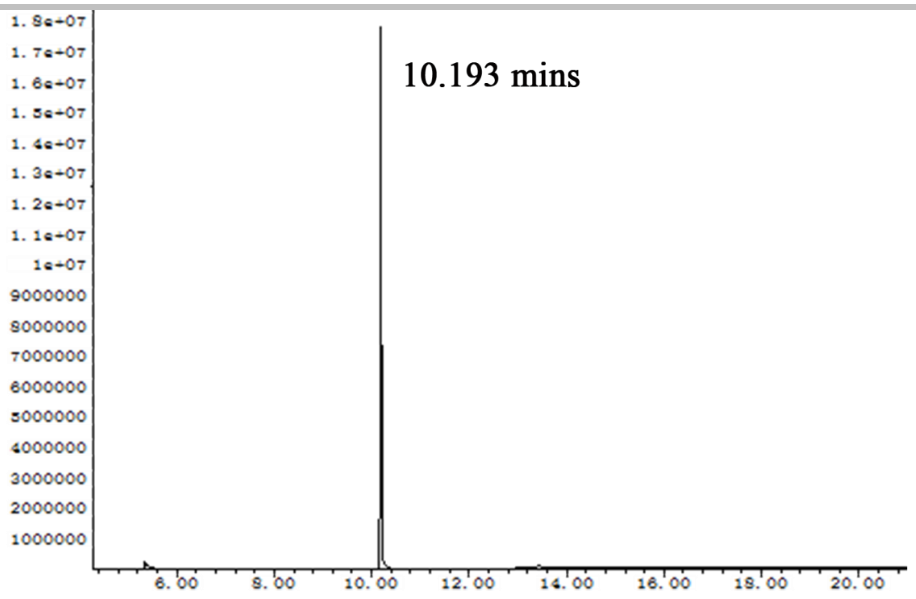

Figure S33. GC spectra of crude product of reaction (containing TEMPO).

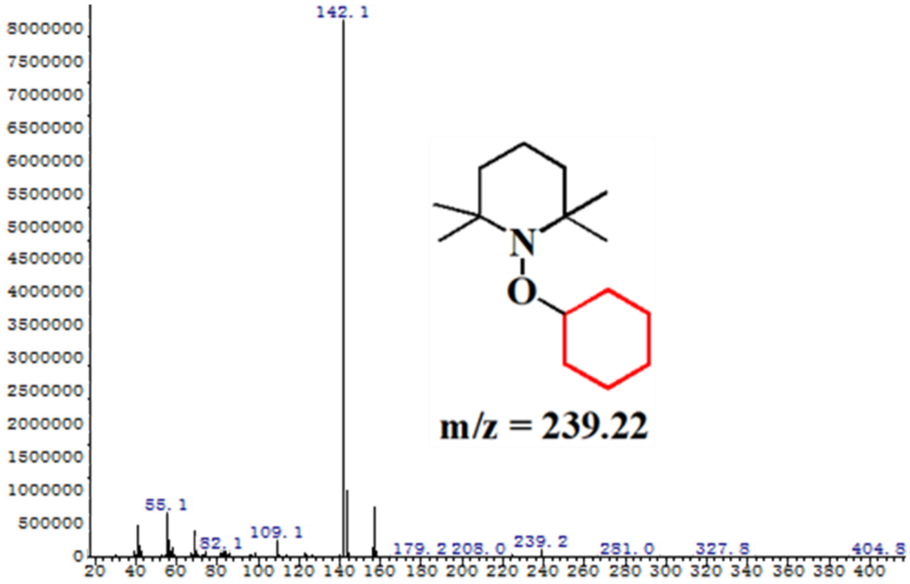

Figure S34. GC-MS spectra of the product with retention time of 10.193 minutes.

To further investigate the carbon radicals generated during the photocatalytic system, 0.5 or 1 equiv. radical trap 2,2,6,6-tetramethylpiperidine-1-oxyl (TEMPO) was added in the model reaction under standard conditions. Both ^{1}H-NMR and GC-MS exhibited the decrease or disappearance of C-N bond formation product and the appearance of coupling product between TEMPO and cyclohexane.[3] These results demonstrated the generation of alkyl radicals was attributed to the LMCT process between **Fe_3-MOF** and Cl^{-1} ions under irradiation.

7. Data Relative to Photocatalytic Activation of Inert C(sp^3)-H Bonds (HAT).

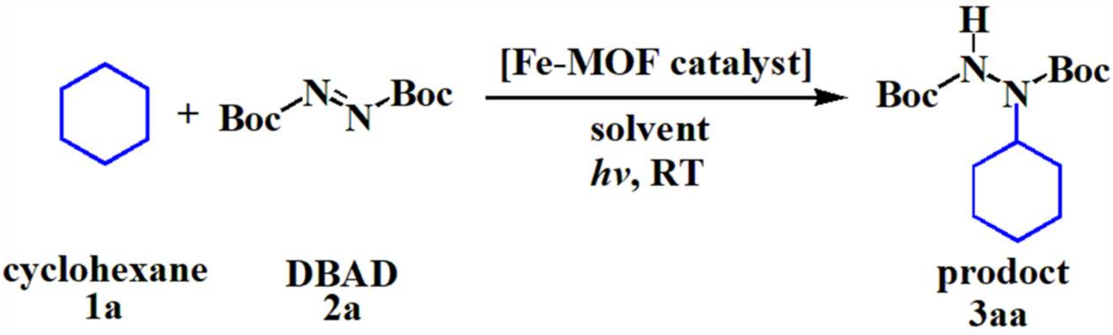

Table S3. Optimisation of conditions for photo-induced **Fe_3-MOF** catalysed C-N bond formation between cyclohexane (1a) and DBAD (2a)[a]

Entry	Catalyst	Solvent	Time (h)	Yield (%)
1	**Fe_3-MOF**	CH_3CN	2.5	98
2[b]	----	CH_3CN	2.5	12
3[c]	**Fe_3-MOF**	CH_3CN	2.5	0
4[d]	**Fe_3-MOF**	CH_3CN	2.5	0
5[e]	**Fe_3-MOF**	CH_3CN	2.5	0
6[f]	**Fe_3-MOF**	CH_3CN	2.5	47
7	**Fe_3-MOF**	CH_3CN	1	80
8	**$Fe(NO_3)_3$**	CH_3CN	1	0
9	**$Fe_2(SO_4)_3$**	CH_3CN	1	0
10	**Fe_3 cluster**	CH_3CN	1	0
11[g]	**Fe_3 cluster + Cl^{-1}**	CH_3CN	1	64
12	**Fe_3 cluster + Cl^{-1}+ H_3NTB**	CH_3CN	1	73
13	**Fe_3-MOF**	CH_2Cl_2	1	11
14	**Fe_3-MOF**	DMSO	1	0

[a]Reaction conditions: N_2 atmosphere and irradiation with 30 W 395 nm LED, cyclohexane (1a) (2 mmol, 10 equiv.), DBAD (2a) (0.2 mmol, 1 equiv.), Fe catalyst (0.625 μmol, 0.005 equiv.), additive (pyridine hydrochloride, 0.5625 mmol, 0.045 equiv.), CH_3CN (2 mL), temperature (rt, 25 °C), in a 16 mL quartz tube. Yields of 3aa were determined by ^{1}H NMR using dibromomethane as the internal standard. [b]No catalyst. [c]No additive. [d]No light. [e]Irradiation with 30 W 455 nm LED. [f]Irradiation with 30 W 405 nm LED. [g]Cl^{-1} = pyridine hydrochloride. DBAD = ditertbutyl azodicarboxylate.

Table S4. Control experiments on chlorine salt.

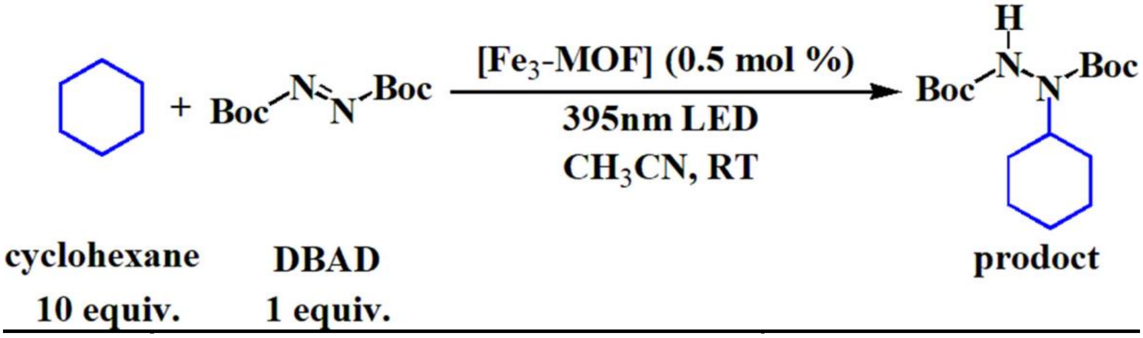

Entry	Chlorine salt	Yield (2.5 h)
1	0.045 equiv. Tetraethylammonium	25%
2	0.045 equiv. Ammonium chlorine	77%
3	0.045 equiv. pyridine hydrochloride	98%
4	0.045 equiv. Pyridine hydrobromide	0%
5	0.045 equiv. Hydrochloric acid	63%

Table S5. Control experiments on amount of chlorine salt

Entry	Amount of chlorine salt	Yield (2.5h)
1	0.010 equiv. pyridine hydrochloride	55%
2	0.025 equiv. pyridine hydrochloride	83%
3	0.045 equiv. pyridine hydrochloride	98%
4	0.1 equiv. pyridine hydrochloride	98%

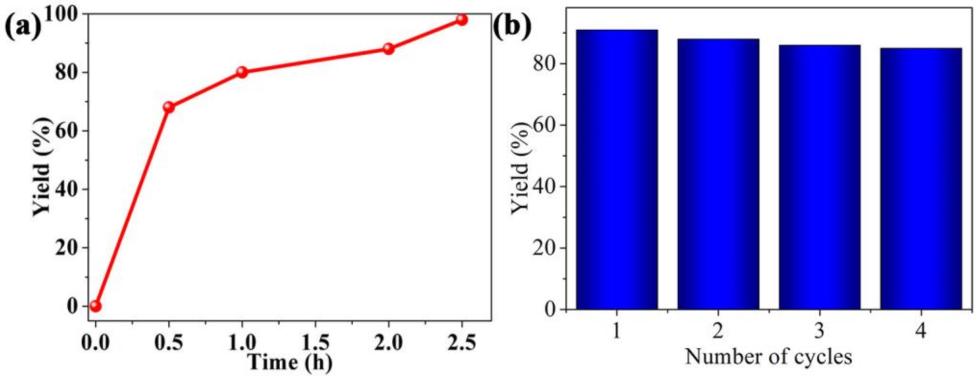

Figure S35. (a) Time-dependent catalytic yield of C(sp^3)-H bonds activation of cyclohexane (HAT). (b) Cyclic experiments on C(sp^3)-H bonds activation of cyclohexane catalyzed by **Fe_3-MOF**.

8. Data Relative to Photocatalytic Borylation of Inert C(sp^3)-H Bonds.

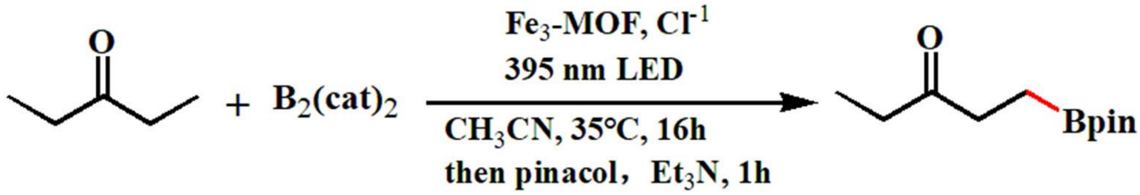

Table S6. Optimization Study for C(sp^3)-H Borylation.[a]

Entry	Variation from the standard conditions	Yield (%)
1	None	51
2	No light	0
3	without **Fe_3-MOF**	2
4	30w 395nm instead of 10w 395nm	10
5	30w 405 nm instead of 10w 395nm	0
6	Without Cl^{-1}	0
7	DMF instead of CH_3CN	5
8	acetone instead of CH_3CN	0

[a] Standard condition: Ar atmosphere and irradiation with an 10 W 395 nm LED, R-H (2 mmol, 10 equiv.), $B_2(cat)_2$ (0.2 mmol, 1 equiv.), **Fe_3-MOF** (0.04 mmol, 0.2 equiv.), additive (pyridine hydrochloride, 0.04 mmol, 0.2 equiv.), CH_3CN (2 mL), temperature (rt, 35 °C), in a 20 mL round-bottle Schlenk bottle, 16 h. Then pinacol (0.24 mmol, 1.2 equiv) in triethylamine (1 mL, 0.2 M), 1 h.

9. Activated Oxygen Performance.

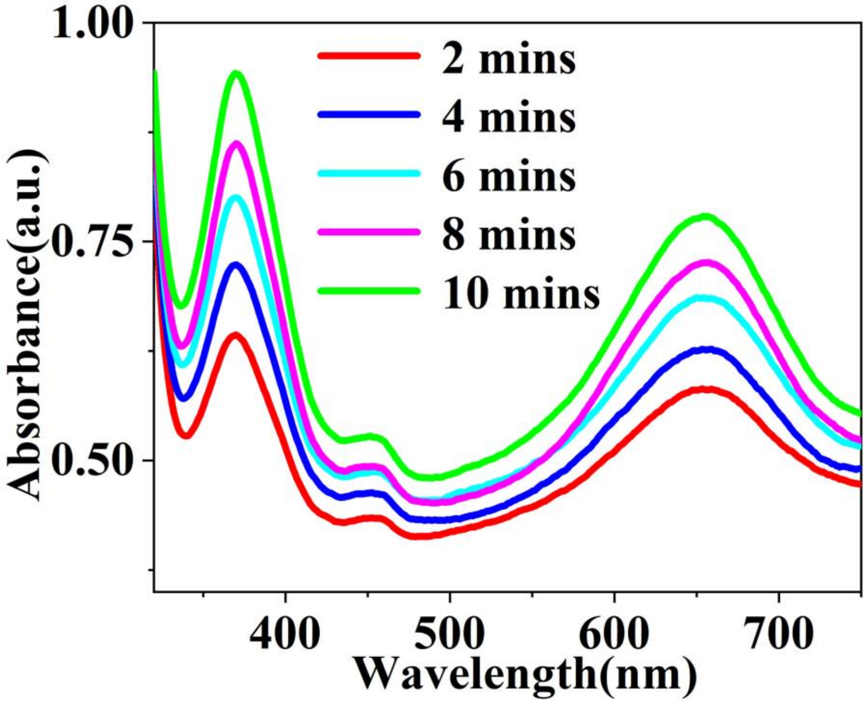

Figure S36. UV-Vis spectra of **Fe_3-MOF** in the presence of TMB in air under 395nm LED irradiation.

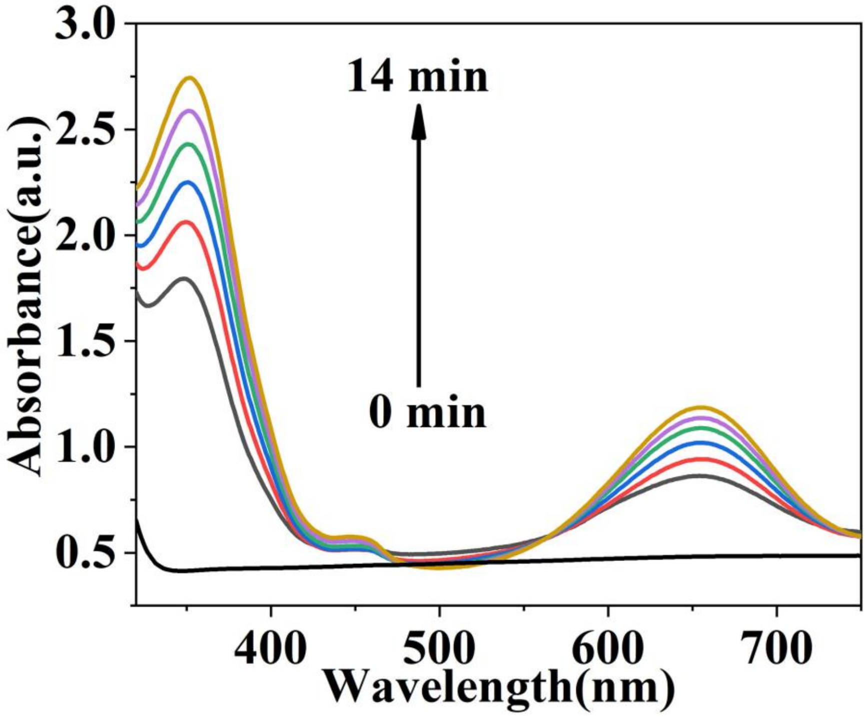

Figure S37. UV-Vis spectra of H_3TCA in the presence of TMB in air under 395nm LED irradiation.

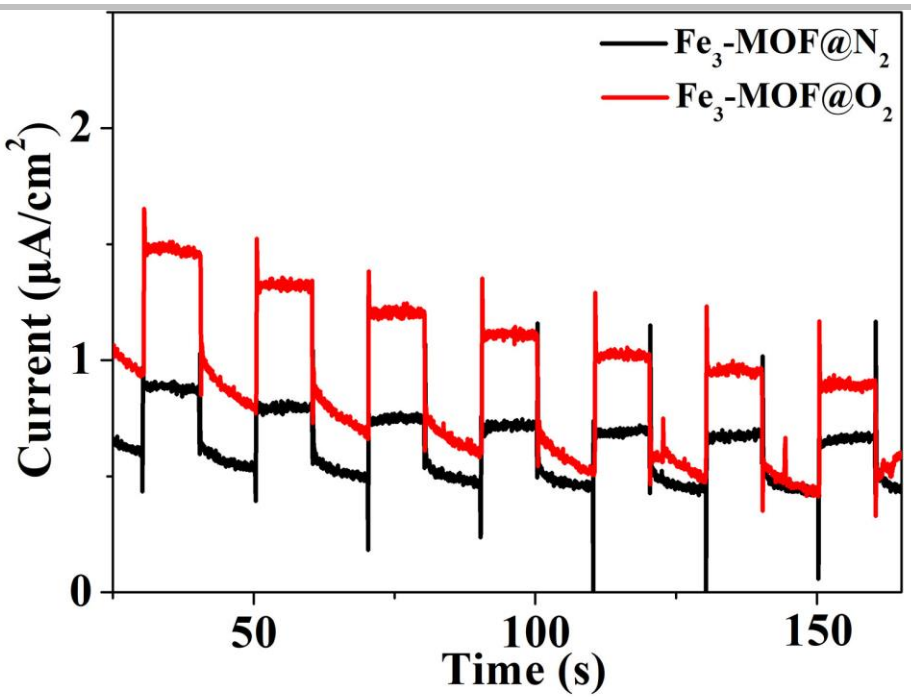

Figure S38. Comparison transient photocurrent responses for **Fe_3-MOF** under N_2 (black line) and (red line) O_2 atmosphere in 0.1 M KCl aqueous solution.

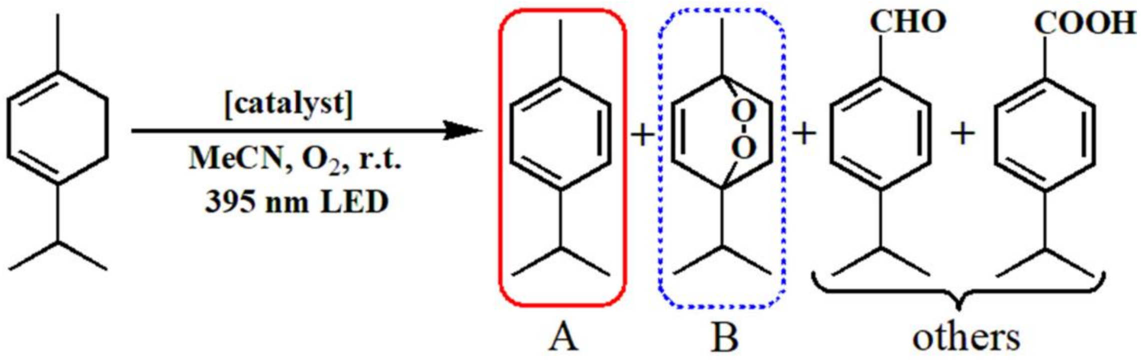

Table S7. Photocatalytic oxidation of α-terpinene.

Entry	Catalyst	Atm	Selectivity/%	
			A	B
1	**Fe_3-MOF**	O_2	80	0
2	**H_3TCA**	O_2	81	9
3	**$FeCl_3$**	O_2	90	0
4	**H_3TCA + $FeCl_3$**	O_2	90	0
5	**----**	O_2	0	0

Reaction conditions: α-terpinene (0.2 mmol), catalyst (0.5 mol%) in acetonitrile (2 mL) under irradiation with a 395 nm LED in oxygen atmosphere within 1 hour. The selectivity was determined by GC-MS analysis.

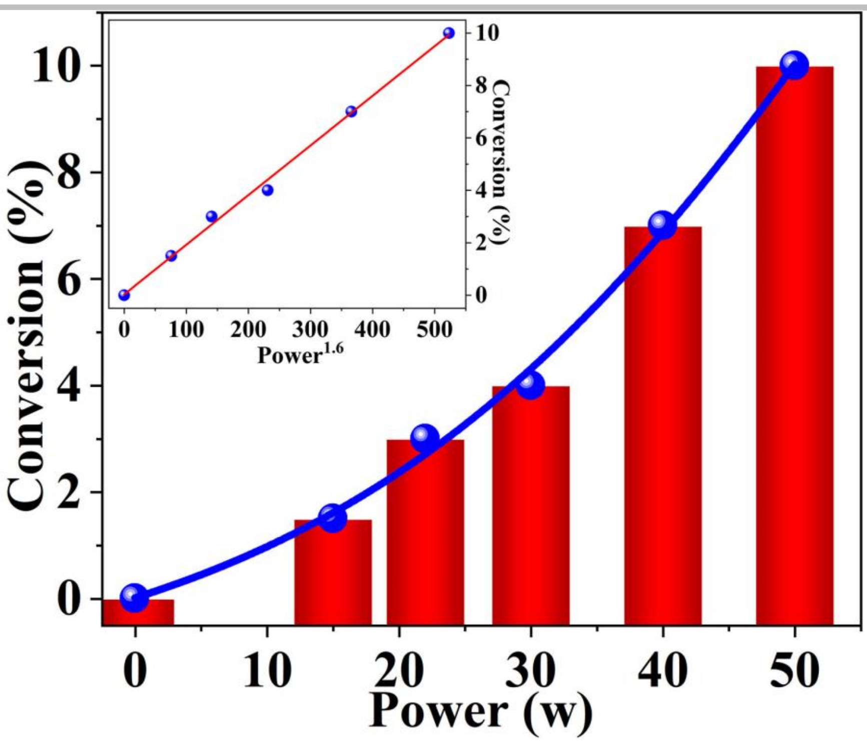

Figure S39. The oxidation conversions of ɑ-terpinene as a function of irradiation powers under the standard conditions with a 395 nm LED irradiation within 2 mins. Inset shows the linear relationship between the yields of the photocatalysis and the times n of photon powers.

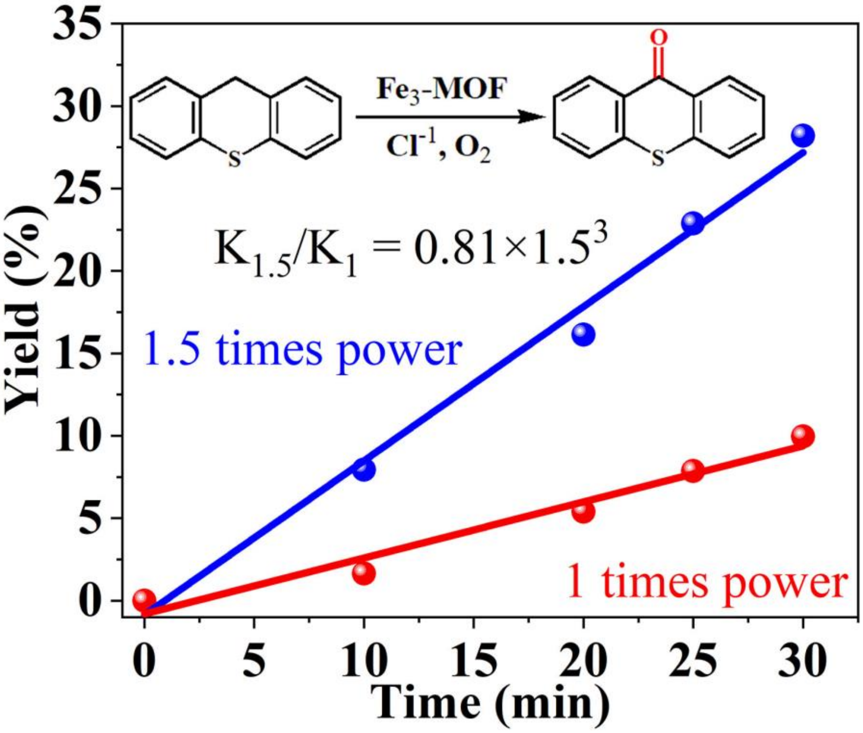

Figure S40. Two oxidation yields of thioxanthene thioxanthene as a function of the time under the standard conditions with a 395 nm LED irradiation of 1.5 times power and 1 times power within 30 mins, respectively.

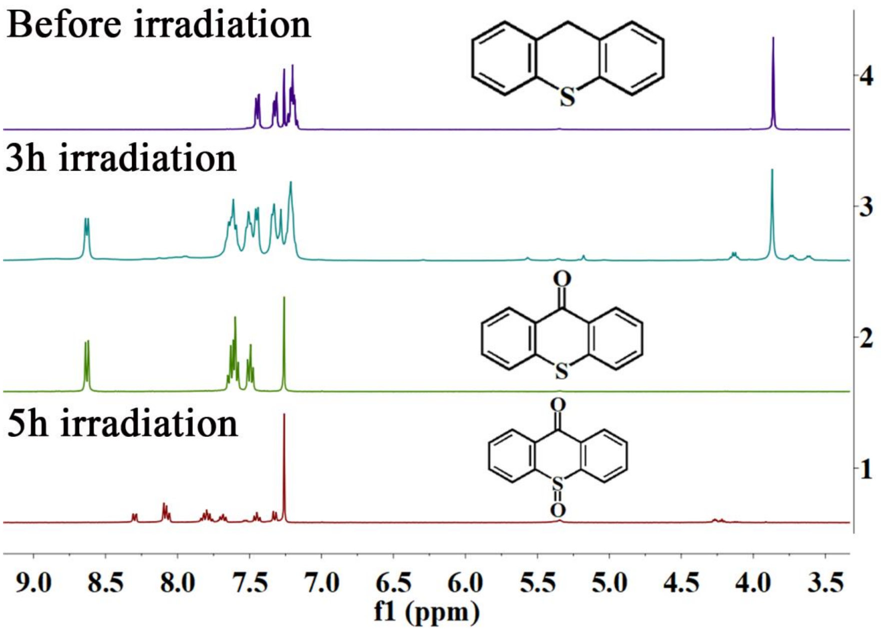

Figure S41. Kinetic profiles of the oxidation of thioxanthene to thioxanthen-9-one and thioxanthone sulfoxide catalyzed by **Fe_3-MOF** and Cl^{-1} under 395 nm LED.

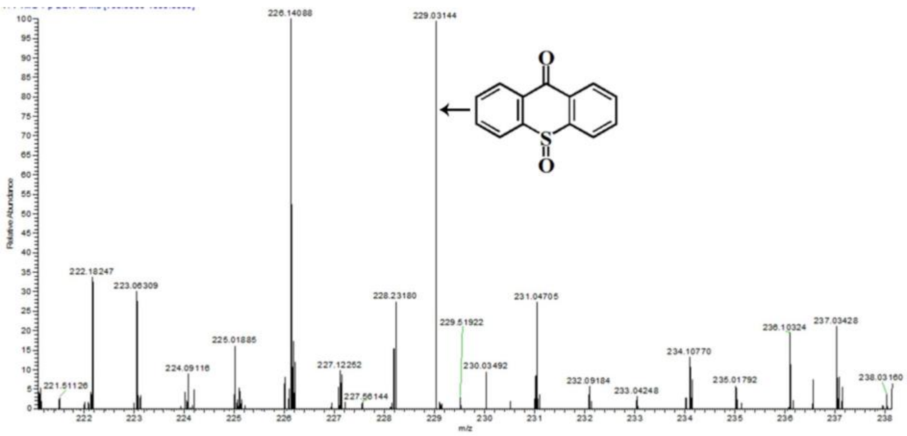

Figure S42. LC-MS spectra of Thioxanthen-9-one sulfoxide. HRMS (ESI+): calcd for $C_{13}H_8O_2S$ $[M+H]^+$ 229.02, found 229.03.

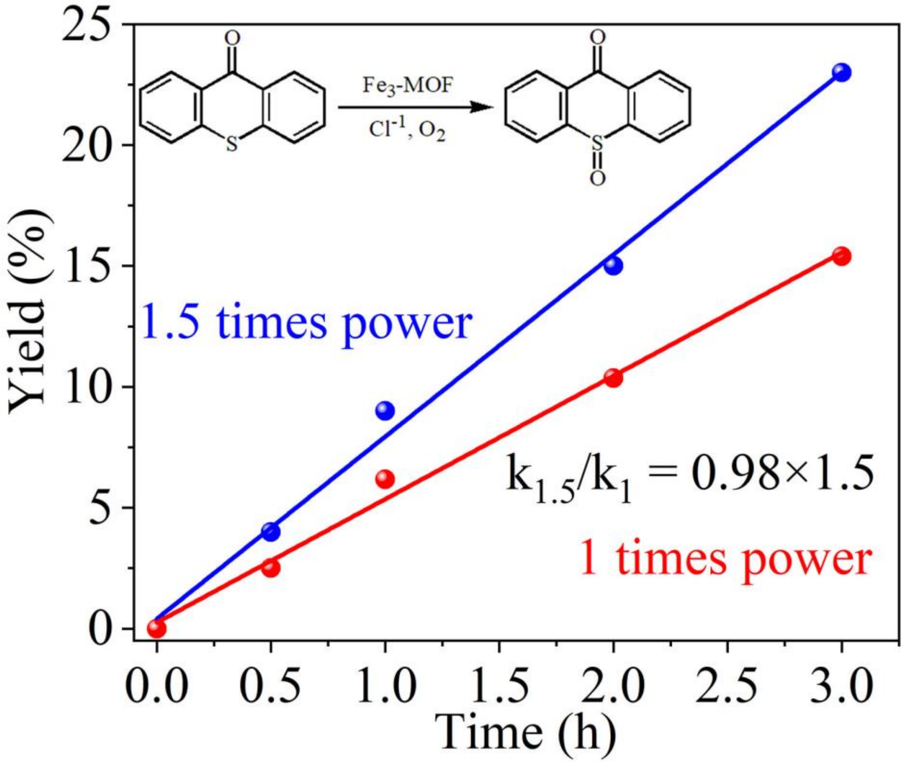

Figure S43. Two oxidation yields of thioxanthen-9-one as a function of the time under the standard conditions with a 395 nm LED irradiation of 1.5 times power and 1 times power within 3 hours, respectively.

10. Data Relative to Photocatalytic Arylation of Inert C(sp^3)-H Bonds.

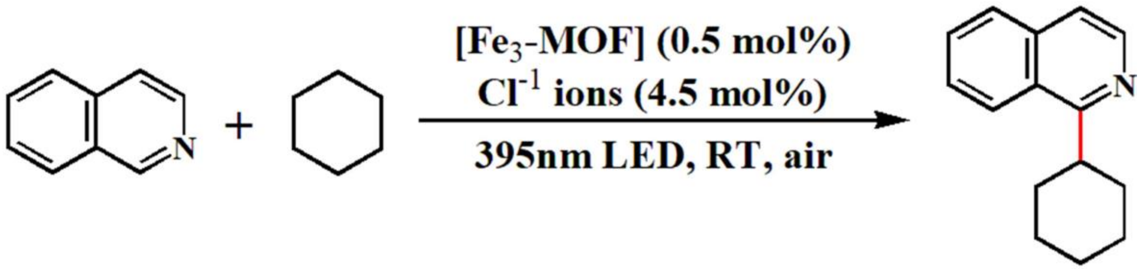

Table S8. Optimization study for C(sp^3)-H arylation.[a]

Entry	Catalyst	Gas	Time (h)	.Yield (%)
1	**Fe_3-MOF**	air	6	91
2	**Fe_3-MOF**	O_2	6	93
3[c]	**Fe_3-MOF**	air	6	27
4[d]	**Fe_3-MOF**	air	6	0
5[e]	**Fe_3-MOF**	air	6	0
6[f]	**----**	air	6	6
7[g]	**Fe_3-MOF**	air	6	19

[a]Reaction conditions: Air atmosphere and irradiation with 30 W 395 nm LED, cyclohexane (2 mmol, 10 equiv.), isoquinoline (0.2 mmol, 1 equiv.), catalyst (0.001 mmol, 0.005 equiv.), Cl^{-1} additive (0.009 mmol, 0.045 equiv.), DCE (2 mL), temperature (rt, ~25 °C), in a 16 ml quartz tube. [b]Yields of product were determined by ^{1}H NMR using dibromomethane as the internal standard. [c]Cl^{-1} additive is NH_4Cl. [d]No Cl^{-1} additive. [e]No light. [f]No catalyst. [g]The wavelength of light source is 405 nm.

Table S9. Screening of solvents in **Fe_3-MOF**-catalyzed activation and arylation of cyclohexane C(sp^3)-H bonds.[a]

Entry	Catalyst	Solvent	Time (h)	Conversion (%)	Yield (%)
1	**Fe_3-MOF**	DCE	6	100	91
2	**Fe_3-MOF**	DMF	6	100	0
3	**Fe_3-MOF**	DMSO	6	83	6
4	**Fe_3-MOF**	acetone	6	79	23
5	**Fe_3-MOF**	chloroform	6	100	16
6	**Fe_3-MOF**	methanol	6	100	12
7	**Fe_3-MOF**	ethyl acetate	6	100	9.3

[a]Reaction conditions: Air atmosphere and irradiation with 30 W 395 nm LED, cyclohexane (2 mmol, 10 equiv.), isoquinoline (0.2 mmol, 1 equiv.), **Fe_3-MOF** (0.001 mmol, 0.005 equiv.), pyridine hydrochloride, (0.009 mmol, 0.045 equiv.), solvent (2 mL), temperature (rt, ~25 °C), in a 16 ml quartz tube. [b]Yields of product were determined by ^{1}H NMR using dibromomethane as the internal standard.

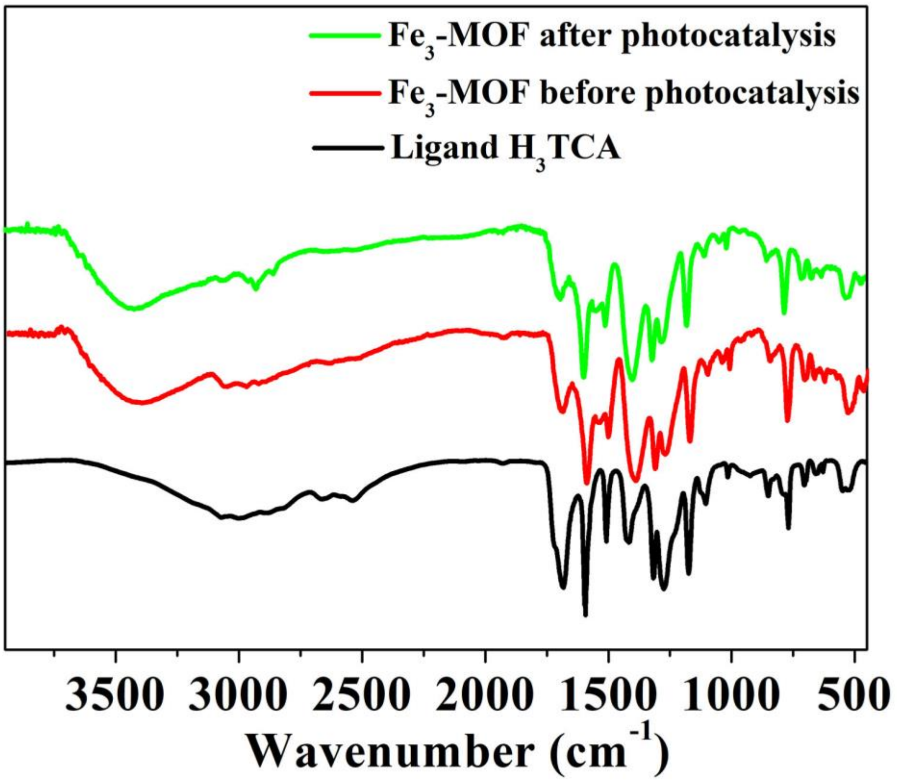

Figure S44. Experimental IR spectra of **Fe3-MOF** before and after photocatalysis.

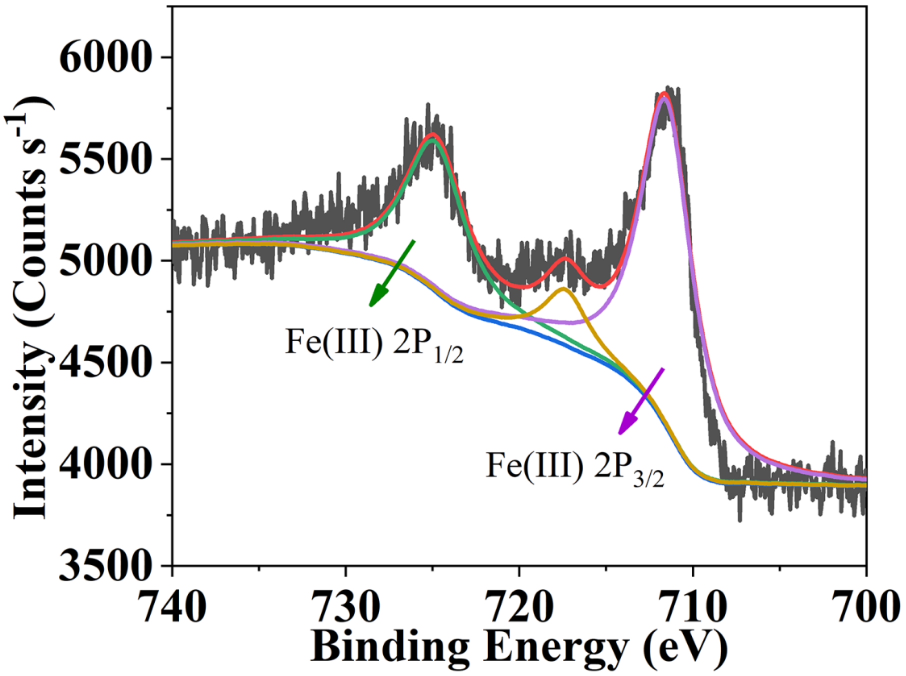

Figure S45. High-resolution XPS spectra of the Fe(2p) for **Fe3-MOF** after photocatalysis with pyridine hydrochloride.

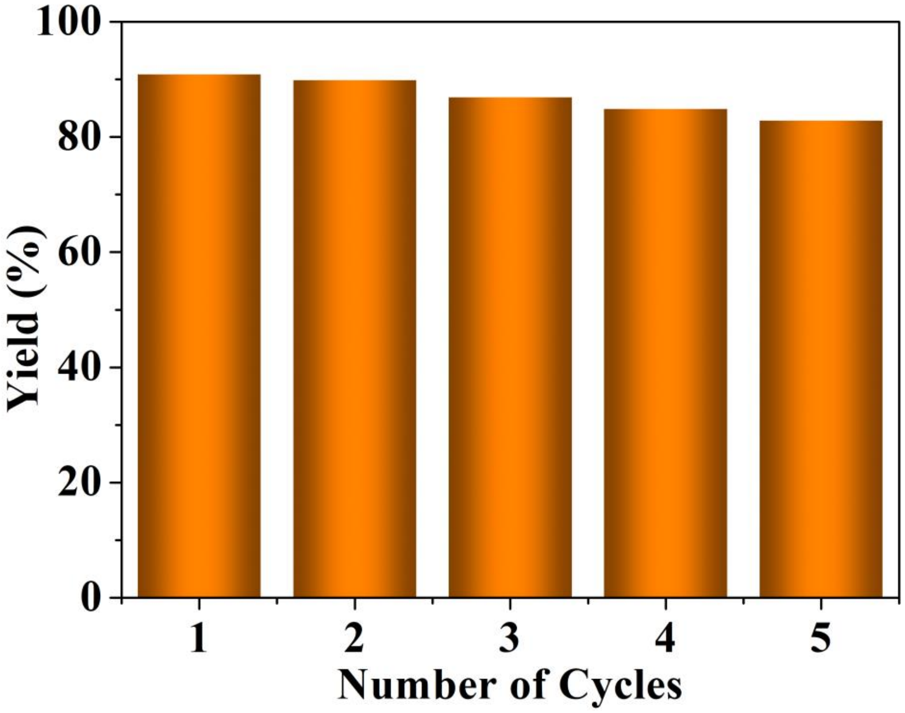

Figure S46. Stability of the reaction of cyclohexane and isoquinoline under optimal reaction conditions in 5 cycles.

11. Data Relative to Photocatalytic Oxidation of Inert C(sp^3)-H Bonds.

Table S10. Optimization study for C(sp^3)-H oxidation.[a]

Entry	Catalyst	Solvent	Time (h)	Yield (%)
1	**Fe_3-MOF** + Cl^{-1}	CH_3CN	2	60
2	**Fe_3-MOF** + Cl^{-1}	CH_3CN	3	93
3	**pyridine hydrochloride**	CH_3CN	2	6.7
4	**Fe_3-MOF (no additive)**	CH_3CN	3	0.3
5	**No light**	CH_3CN	3	0
6	**$FeCl_3$**	CH_3CN	3	47.8

[a]Reaction conditions: O_2 atmosphere and irradiation with 30 W 395 nm LED, substrate (0.2 mmol, 1 equiv.), **Fe_3-MOF** (0.001 mmol, 0.005 equiv.), additive (pyridine hydrochloride, 0.009 mmol, 0.045 equiv.), CH_3CN (2 mL), temperature (rt, ~25 °C), in a 16 mL quartz tube. [b]Yields were determined by GC.

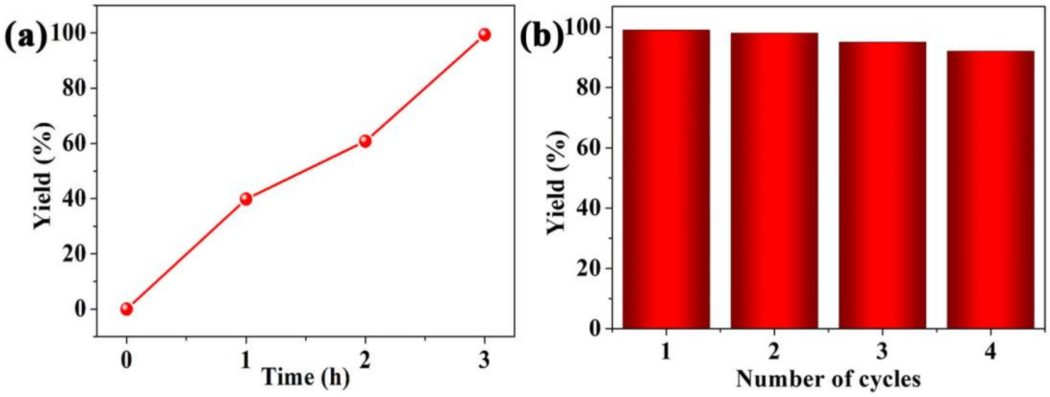

Figure S47. (a) Time-dependent catalytic yield of C-H bond oxidation of diphenylmethane. (b) Cyclic experiments on C-H bond oxidation of diphenylmethane catalyzed by **Fe_3-MOF**.

Artificial metabolic enzyme

Table S11. Fe_3-MOF photocatalyzed oxidative metabolism of C-H bonds.

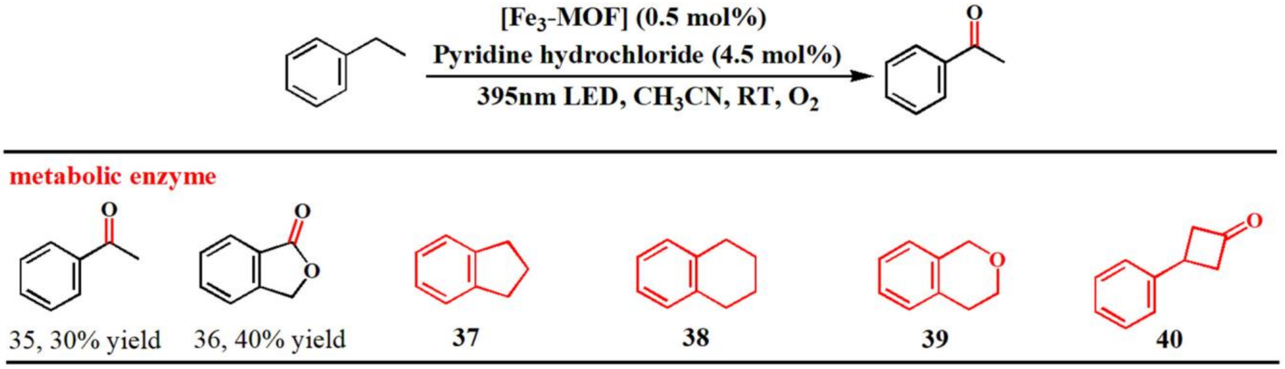

[a]Reaction conditions: O_2 atmosphere and irradiation with 30 W 395 nm LED, substrate (0.2 mmol, 1 equiv.), **Fe_3-MOF** (0.001 mmol, 0.005 equiv.), additive (pyridine hydrochloride, 0.009 mmol, 0.045 equiv.), CH_3CN (2 mL), temperature (rt, ~25 °C), in a 16 mL quartz tube. [b]Products were analyzed by GC-MS.

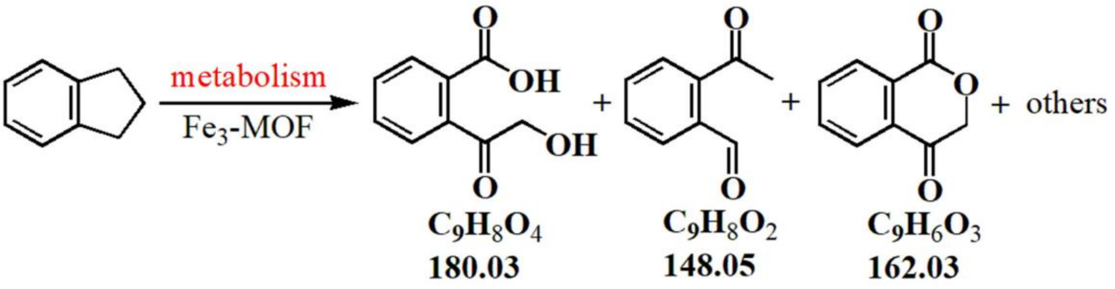

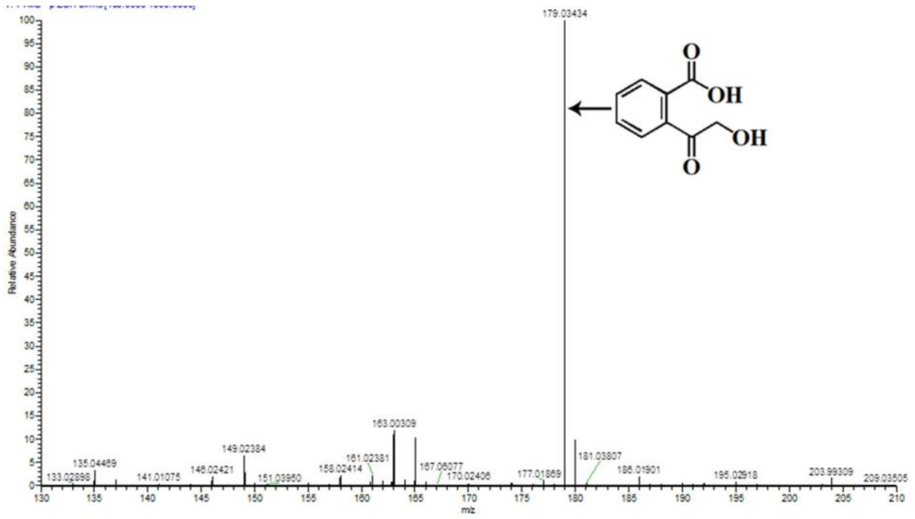

Figure S48. LC-MS spectrum of indane metabolite-1. HRMS-1 (ESI-): calcd for $C_9H_7O_4$ $[M-H]^-$ 179.03, found 179.03.

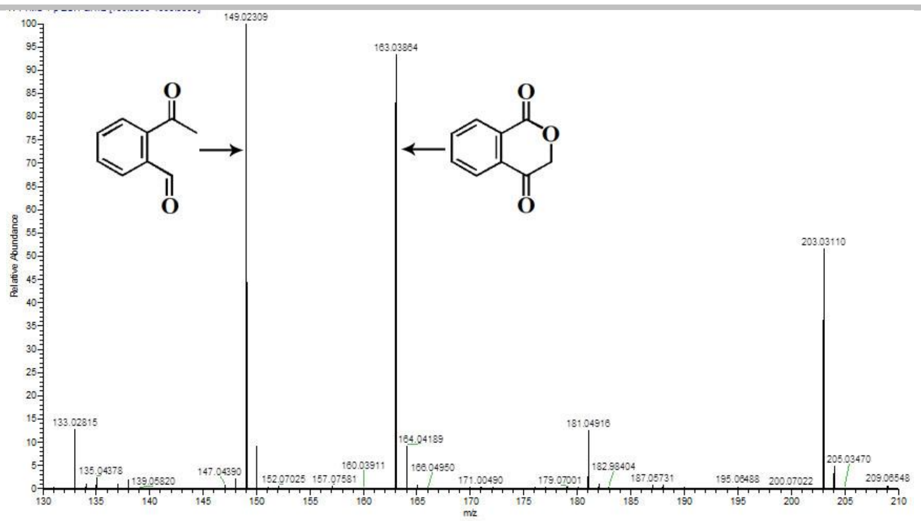

Figure S49. LC-MS spectrum of indane metabolite-2 and metabolite-3. HRMS-2 (ESI+): calcd for $C_9H_9O_2$ $[M+H]^+$ 149.05, found 149.02. HRMS-3 (ESI+): calcd for $C_9H_7O_3$ $[M+H]^+$ 163.03, found 163.03.

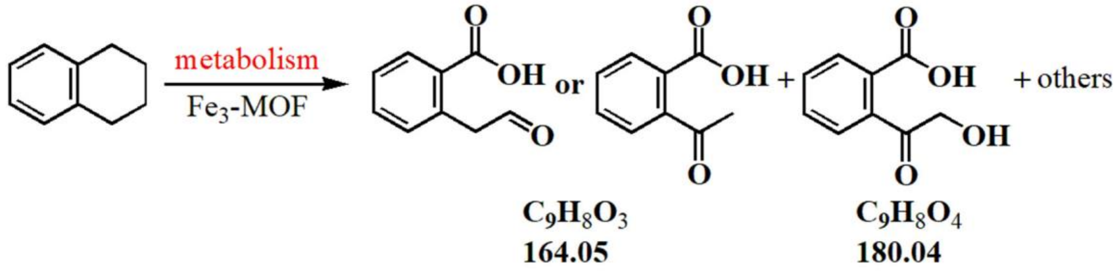

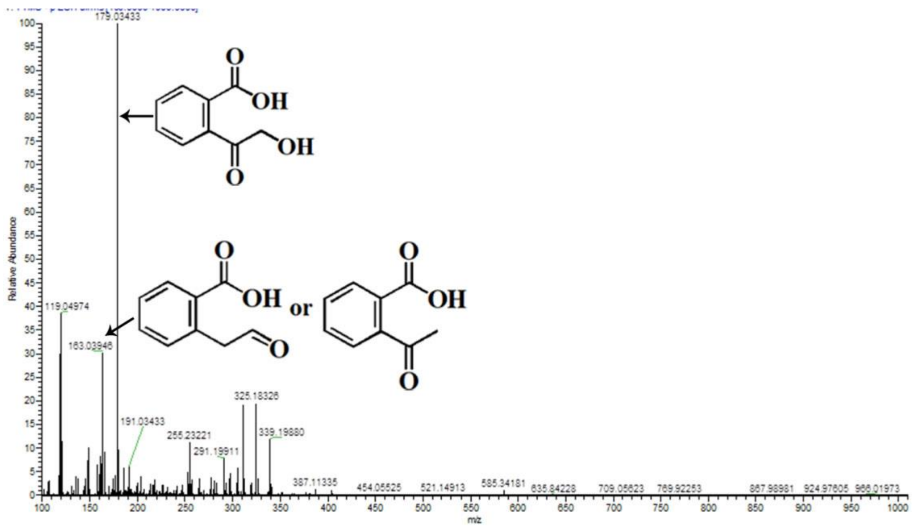

Figure S50. LC-MS spectrum of tetraline metabolite-1 and metabolite-2. HRMS-1 (ESI-): calcd for $C_9H_7O_3$, $[M-H]^-$ 163.05, found 163.04. HRMS-2 (ESI-): calcd for $C_9H_7O_4$ $[M-H]^-$ 179.03, found 179.03.

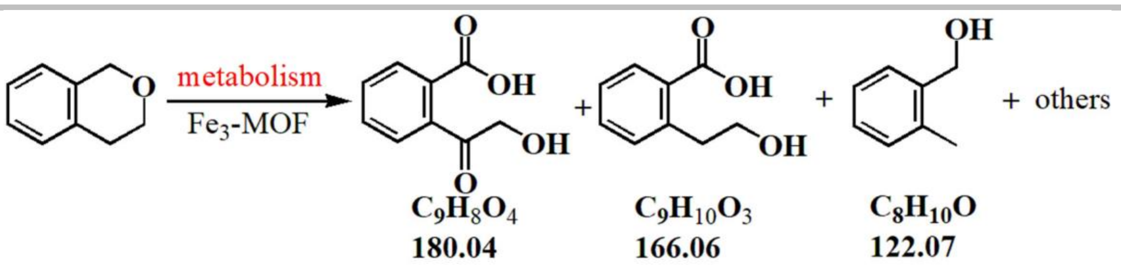

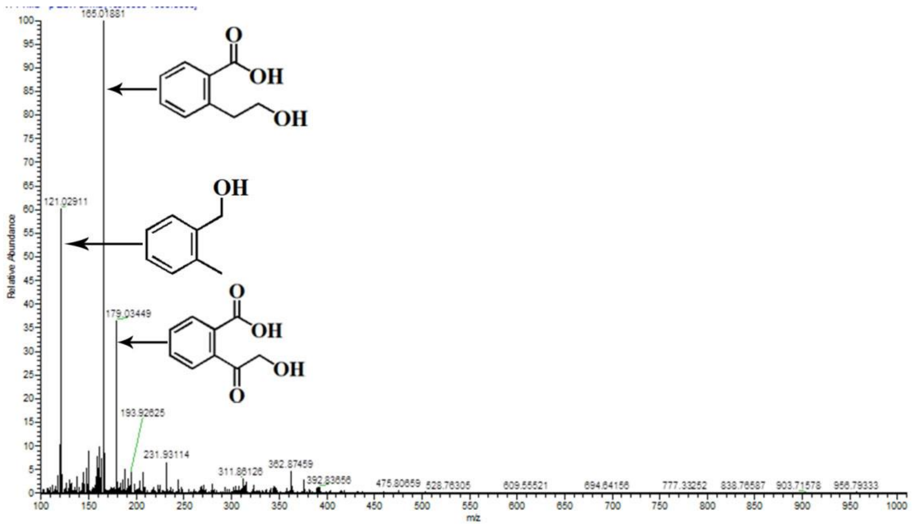

Figure S51. LC-MS spectrum of isochroman metabolite-1, metabolite-2 and metabolite-3. HRMS-1 (ESI-): calcd for $C_9H_7O_4$, $[M-H]^-$ 179.04, found 179.03. HRMS-2 (ESI-): calcd for $C_9H_9O_3$ $[M-H]^-$ 165.06, found 165.02, HRMS-3 (ESI-): calcd for C_8H_9O, $[M-H]^-$ 121.07, found 121.03.

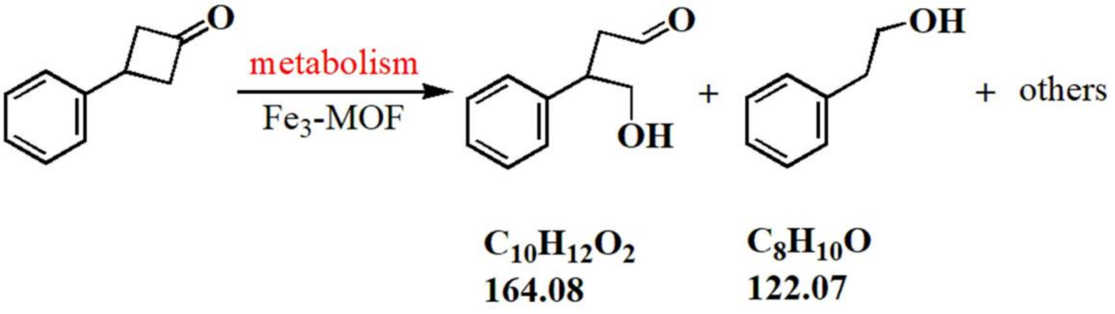

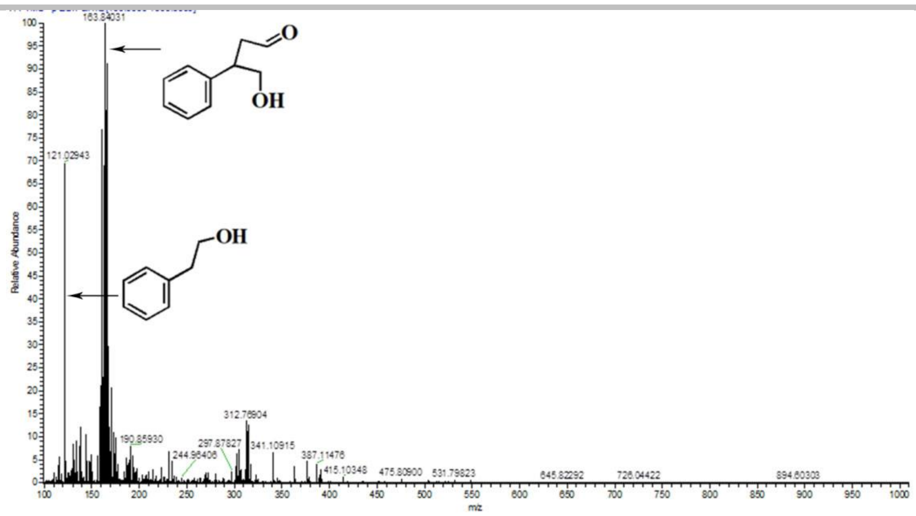

Figure S52. LC-MS spectrum of 3-phenyl-cyclobutan-1-one metabolite-1 and metabolite-2. HRMS-1 (ESI-): calcd for $C_{10}H_{11}O_2$, $[M-H]^-$ 163.08, found 163.84. HRMS-2 (ESI-): calcd for C_8H_9O, $[M-H]^-$ 121.07, found 121.03.

Table S12. Calculation of the proportion of single photon, two photon and three photon excitation processes.

Photocatalytic oxidation of a-terpinene	$0.74 \times 2^2 = x \times 2^1 + (1-x) \times 2^2$
	1 photon, x = 0.52
	2 photon, 1-x = 0.48
Photocatalytic oxidation C-C coupling of isoquinoline and cyclohexane	$0.86 \times 1.5^3 = x \times 1.5^2 + (1-x) \times 1.5^3$
	2 photon, x = 0.42
	3 photon, 1-x = 0.58
Photocatalytic oxidation of thioxanthene	$0.81 \times 1.5^3 = x \times 1.5^2 + (1-x) \times 1.5^3$
	2 photon, x = 0.57
	3 photon, 1-x = 0.43

The above calculation results are converted into Fig. 3h in the manuscript.

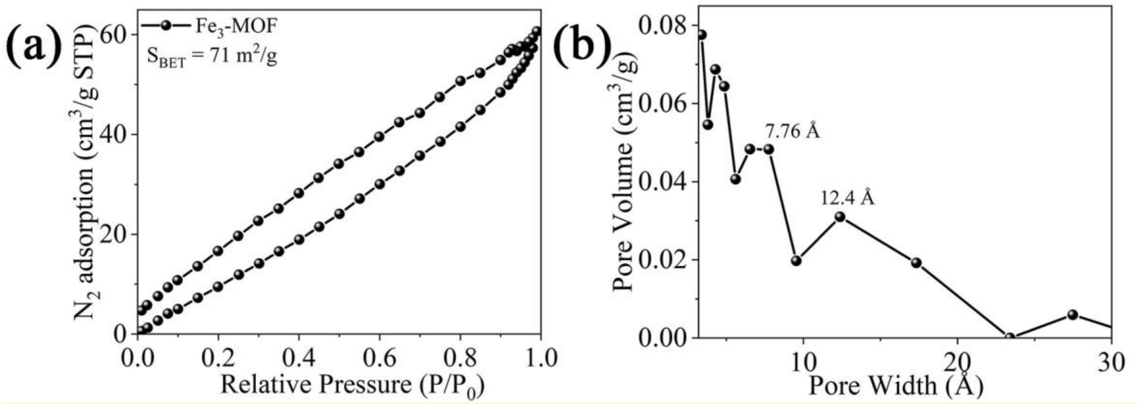

Figure **S53**. (a) N_2 adsorption-desorption of **Fe_3-MOF**. (b) Pore size distribution of **Fe_3-MOF**.

N_2 sorption analysis of **Fe_3-MOF** shows a Brunauer-Emmett-Teller (BET) surface area of 71 m^2 g^{-1}. The main pore sizes are 7.8 Å and 12.4 Å. When the solvent inside pore is removed under vacuum conditions, the pore will change because of the porous material composed of this flexible ligand, resulting in a large bias of N_2 sorption tests, which is greatly different from the single crystal X-ray diffraction data.

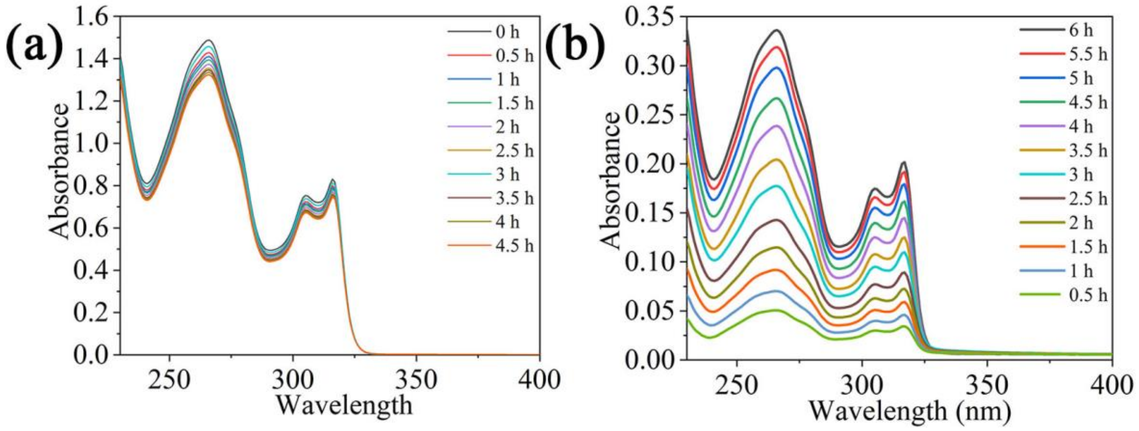

Figure S54. (a) Time-dependent UV-Vis spectrum of isoquinoline (1.5 mM) in acetonitrile solution after adsorption by Fe_3-MOF (5 mg). (b) Time-dependent UV-Vis spectrum of isoquinoline released in acetonitrile solution by the substrate-absorption crystals Fe_3-MOF (5 mg).

Isoquinoline adsorption and desorption followed reversible diffusion-controlled adsorption dynamics along the channels

12. NMR and GC-MS spectra for products

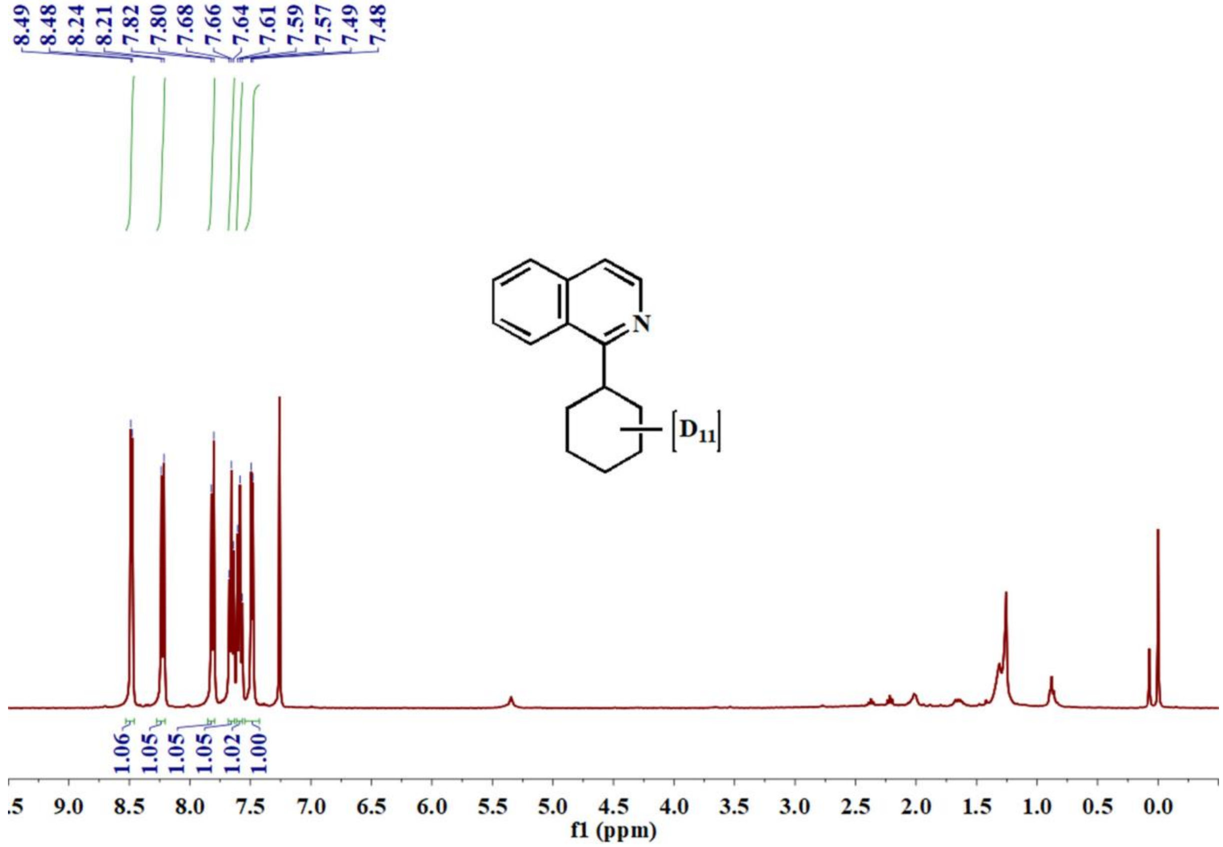

D-1-cyclohexylisoquinoline ^{1}H NMR (400 MHz, $CDCl_3$) δ 8.48 (d, *J* = 5.6 Hz, 1H), 8.22 (d, *J* = 8.4 Hz, 1H), 7.81 (d, *J* = 8.1 Hz, 1H), 7.66 (t, *J* = 7.4 Hz, 1H), 7.59 (t, *J* = 7.6 Hz, 1H), 7.49 (d, *J* = 5.7 Hz, 1H).

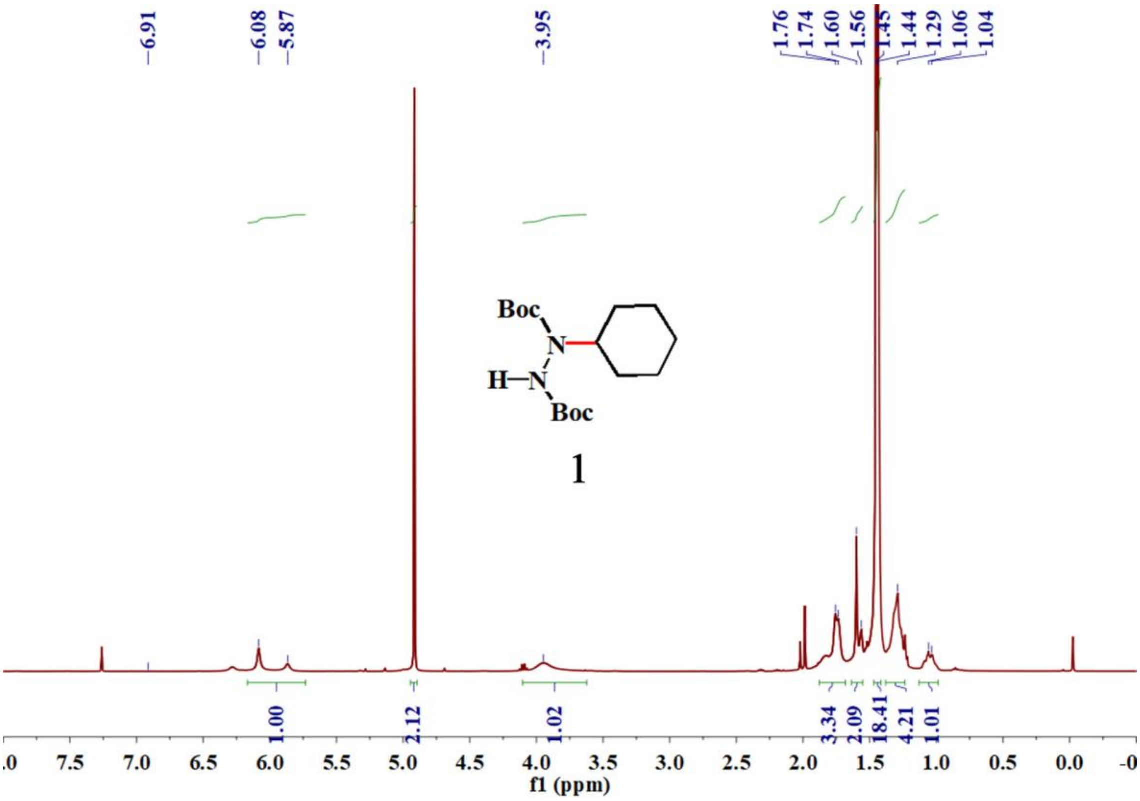

^{1}H NMR (400 MHz, $CDCl_3$) δ 5.97 (d, *J* = 86.9 Hz, 1H), 3.95 (s, 1H), 1.75 (d, *J* = 8.3 Hz, 3H), 1.58 (d, *J* = 14.6 Hz, 2H), 1.44 (d, *J* = 5.3 Hz, 18H), 1.29 (s, 4H), 1.05 (d, *J* = 9.2 Hz, 1H).

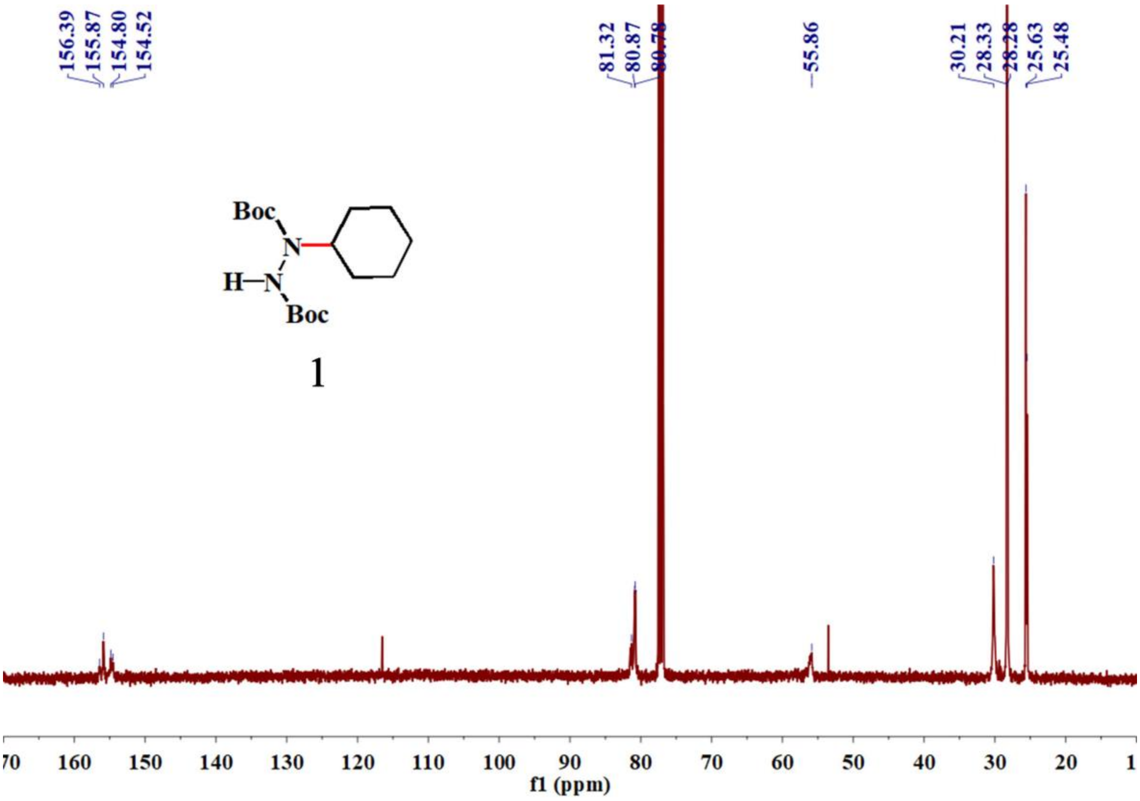

^{13}C NMR (101 MHz, $CDCl_3$) δ 156.39, 155.87, 154.80, 154.52, 81.32, 80.87, 80.78, 55.86, 30.21, 28.33, 28.28, 25.63, 25.48.

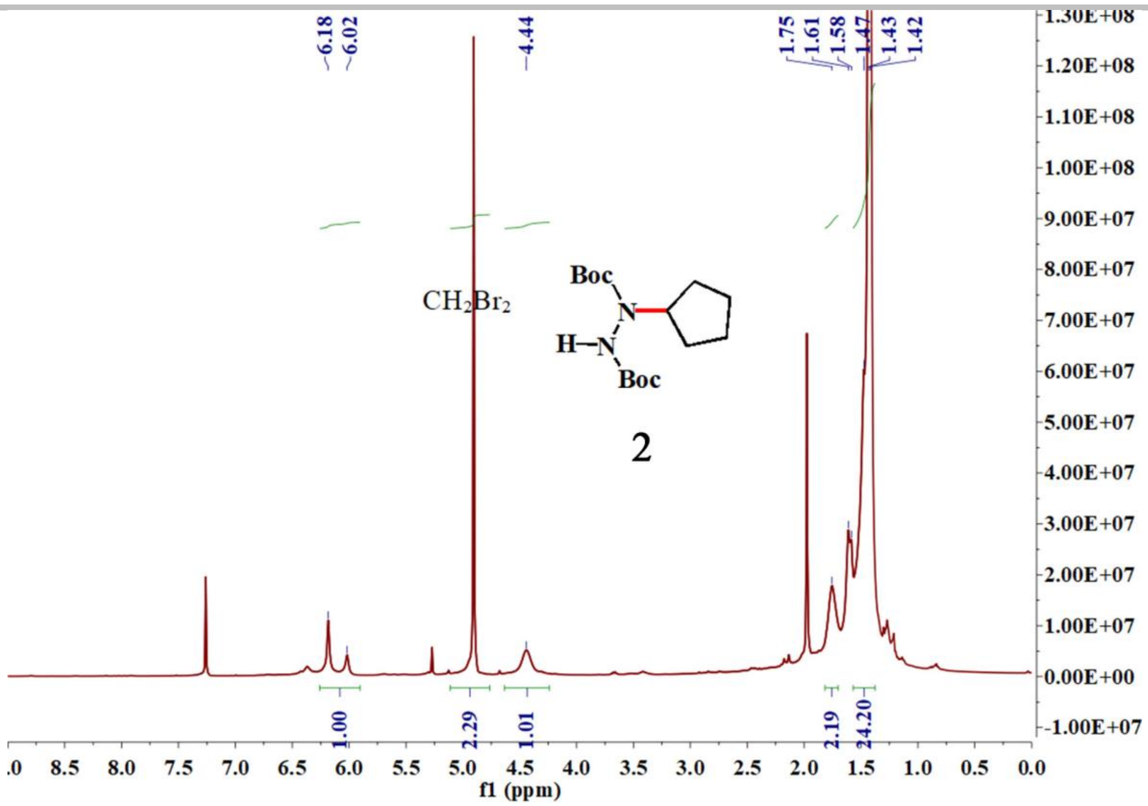

^{1}H NMR spectrum of crude product of 2.

^{1}H NMR (400 MHz, $CDCl_3$) δ 6.10 (d, *J* = 66.5 Hz, 1H), 4.91 (s, 2H), 4.44 (s, 1H), 1.75 (s, 2H), 1.44 (t, *J* = 10.7 Hz, 24H).

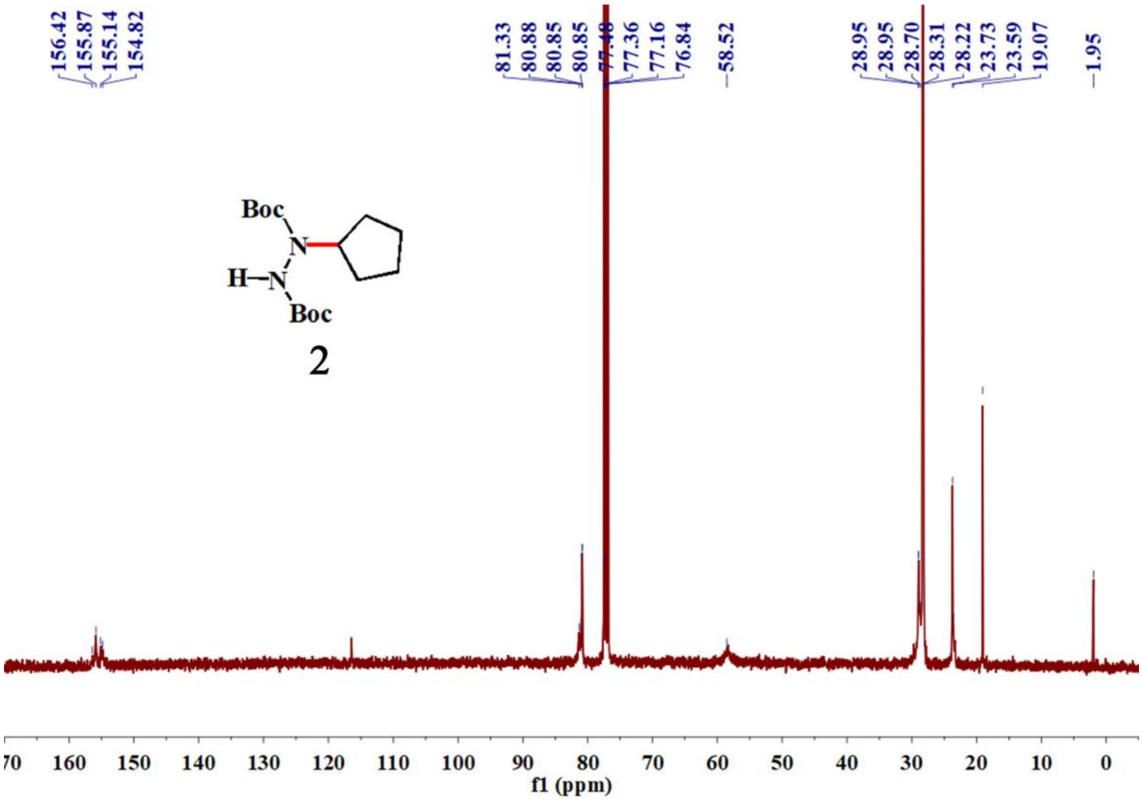

^{13}C NMR (101 MHz, $CDCl_3$) δ 156.42, 155.87, 155.14, 154.82, 81.33, 80.88, 80.85, 58.52, 28.95, 28.70, 28.31, 28.22, 23.73, 23.59.

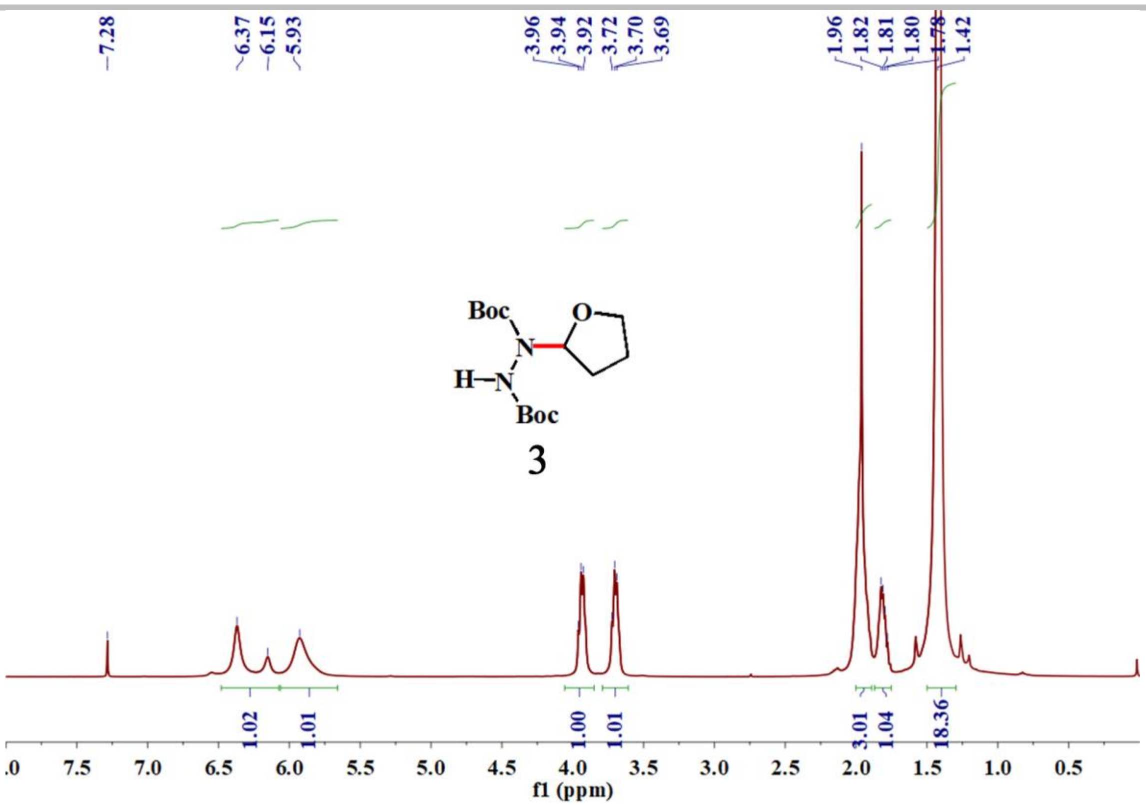

^{1}H NMR (400 MHz, $CDCl_3$) δ 6.26 (d, *J* = 86.8 Hz, 1H), 5.93 (s, 1H), 4.06 – 3.85 (m, 1H), 3.79 – 3.61 (m, 1H), 1.96 (s, 3H), 1.80 (dd, *J* = 11.6, 6.3 Hz, 1H), 1.42 (s, 18H).

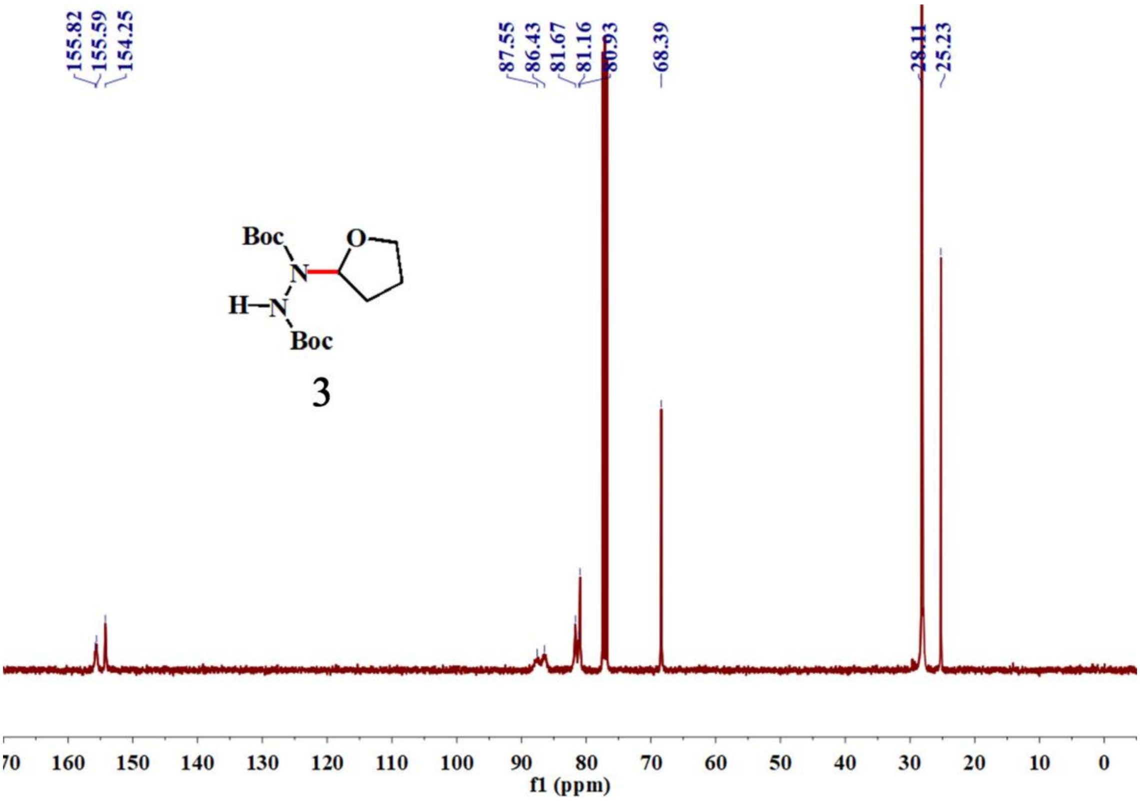

^{13}C NMR (101 MHz, $CDCl_3$) δ 155.82, 155.59, 154.25, 87.55, 86.47, 81.63, 81.21, 80.93, 68.39, 28.11, 25.23.

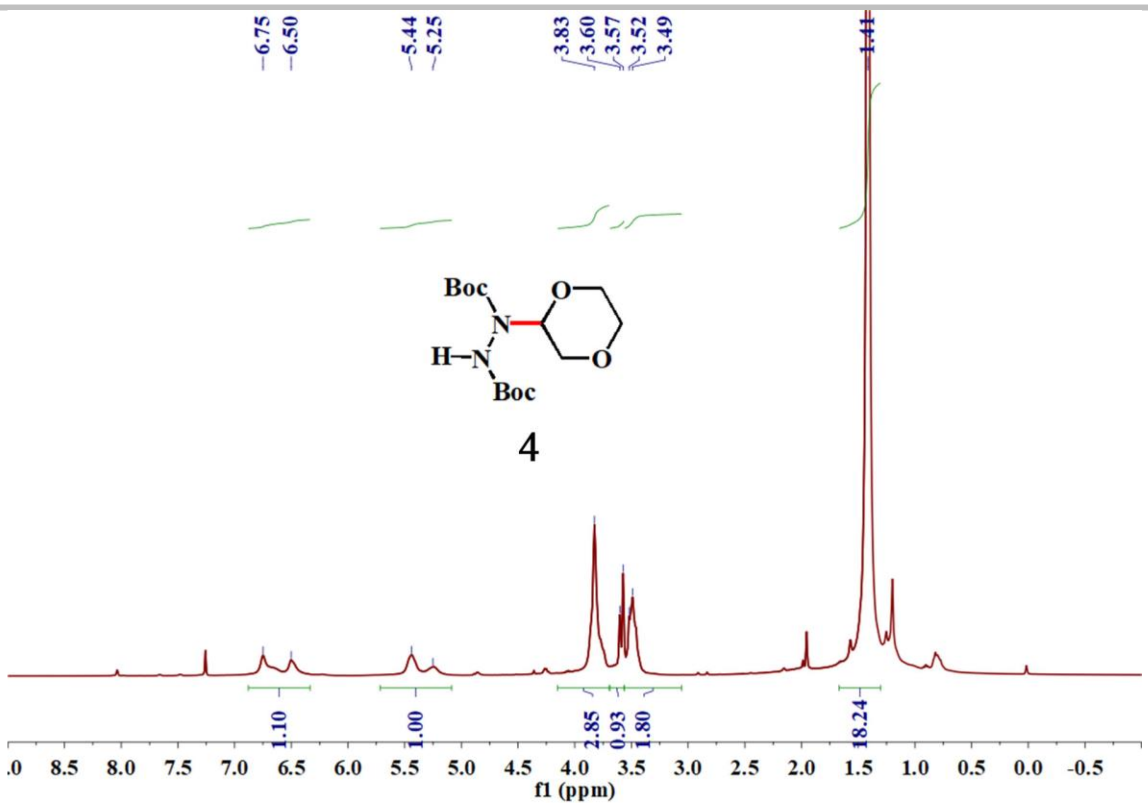

^{1}H NMR (400 MHz, $CDCl_3$) δ 6.63 (d, J = 99.3 Hz, 1H), 5.34 (d, J = 75.1 Hz, 1H), 3.83 (s, 3H), 3.59 (d, J = 11.4 Hz, 1H), 3.50 (d, J = 12.2 Hz, 2H), 1.41 (s, 18H).

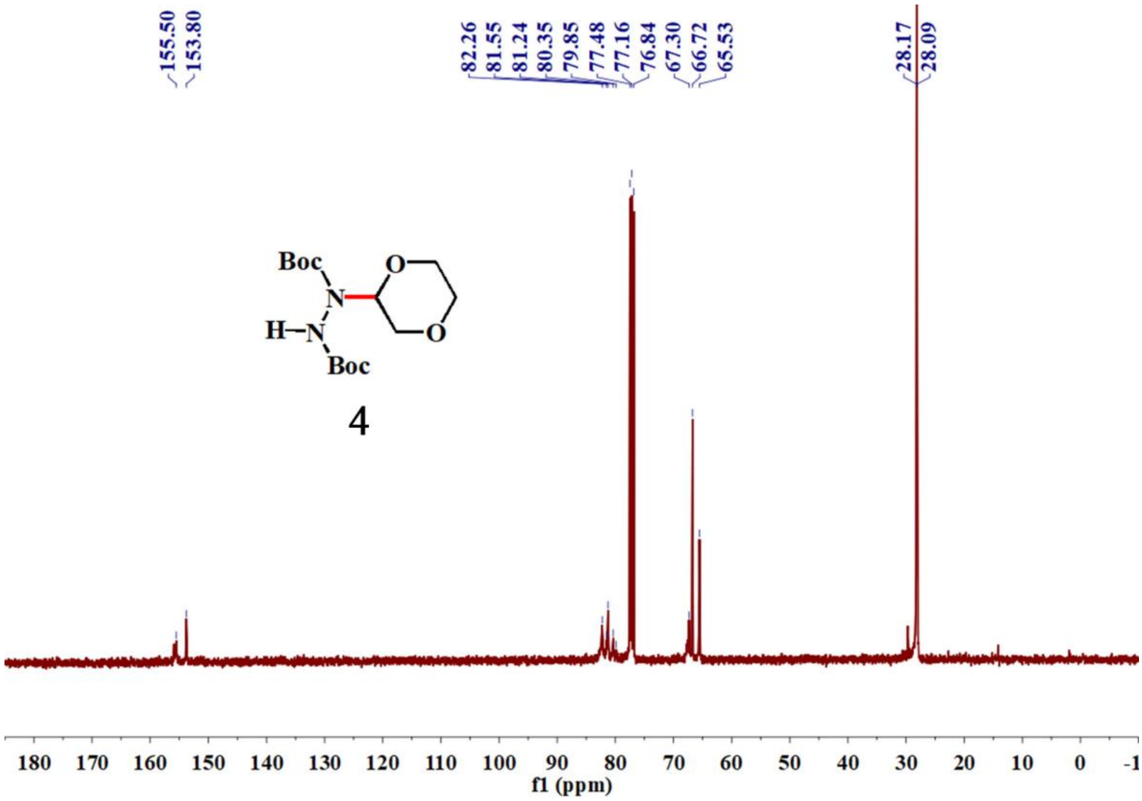

^{13}C NMR (101 MHz, $CDCl_3$) δ 155.50, 153.80, 82.26, 81.55, 81.24, 80.35, 79.85, 67.30, 66.72, 65.53, 28.17, 28.09.

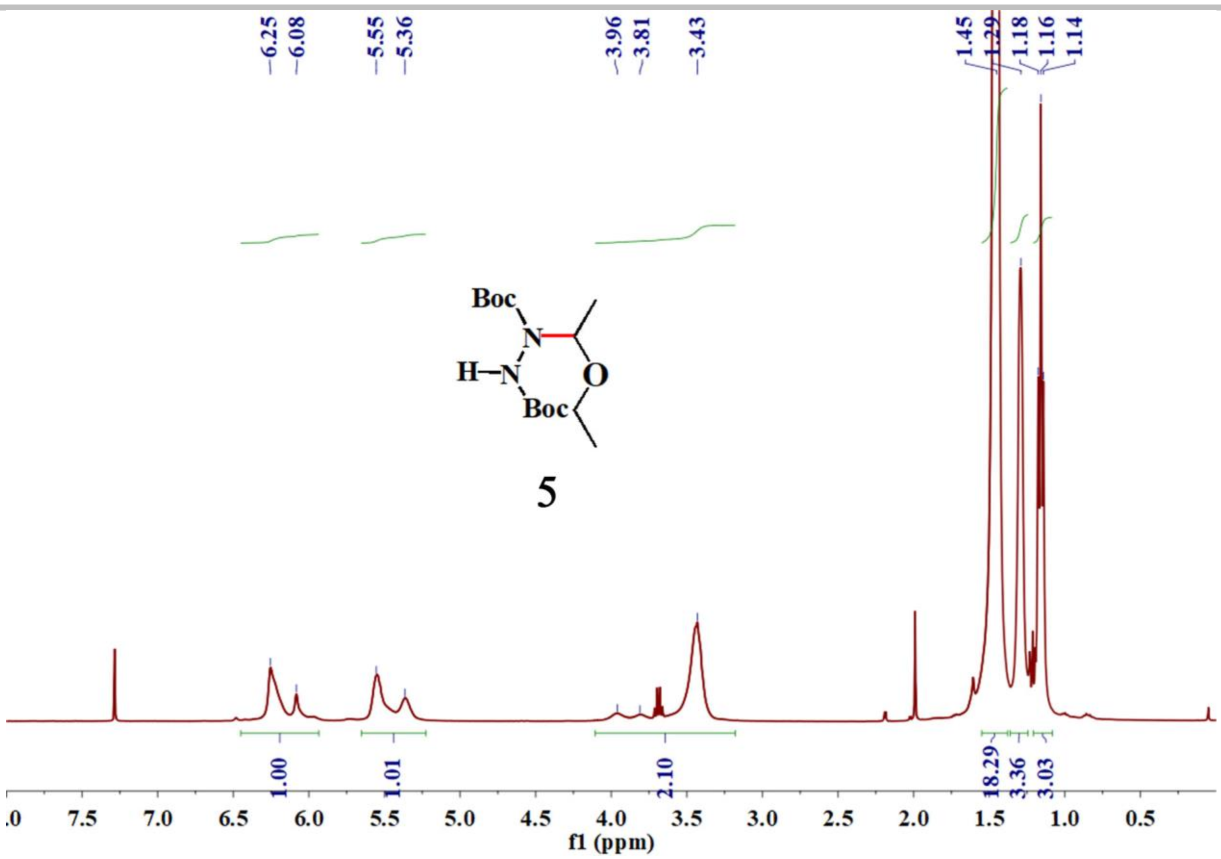

^{1}H NMR (400 MHz, CDCl$_3$) δ 6.17 (d, *J* = 68.5 Hz, 1H), 5.46 (d, *J* = 76.1 Hz, 1H), 4.11 – 3.18 (m, 2H), 1.45 (s, 18H), 1.29 (s, 3H), 1.16 (t, *J* = 6.9 Hz, 3H).

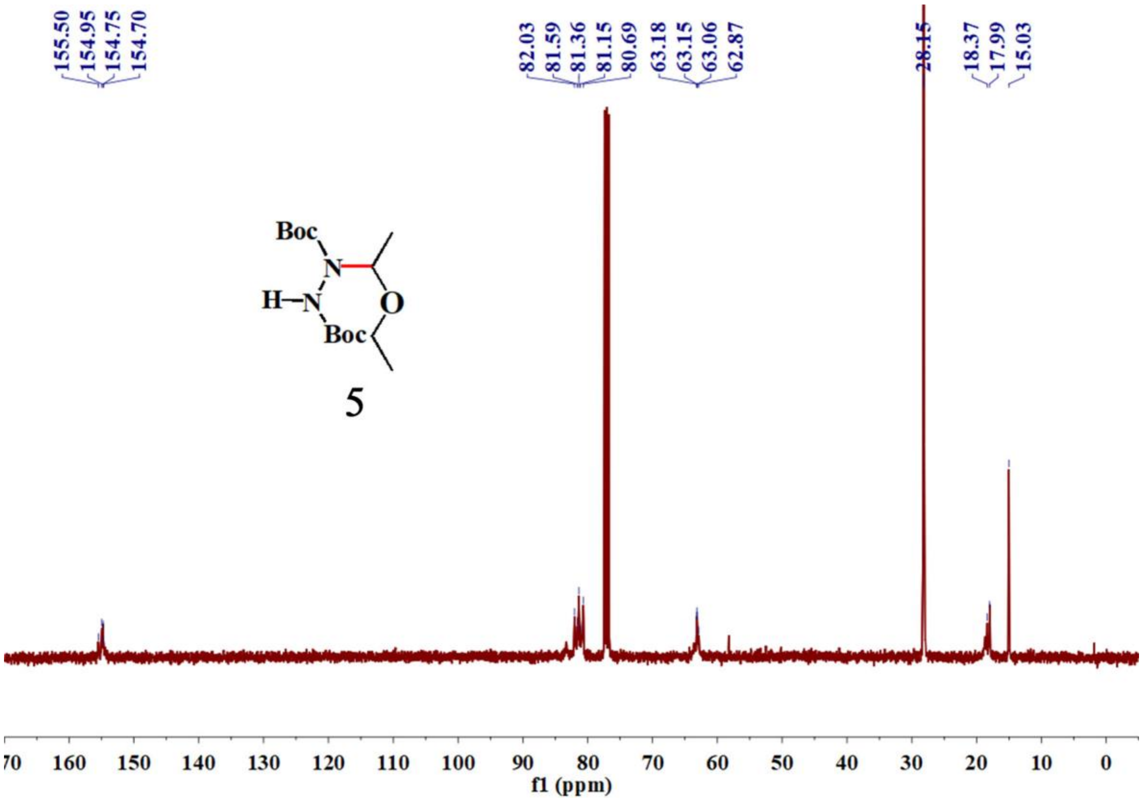

^{13}C NMR (101 MHz, CDCl$_3$) δ 155.50, 154.95, 154.75, 154.70, 82.03, 81.59, 81.36, 81.15, 80.69, 63.18, 63.15, 63.06, 62.87, 28.15, 18.37, 17.99, 15.03.

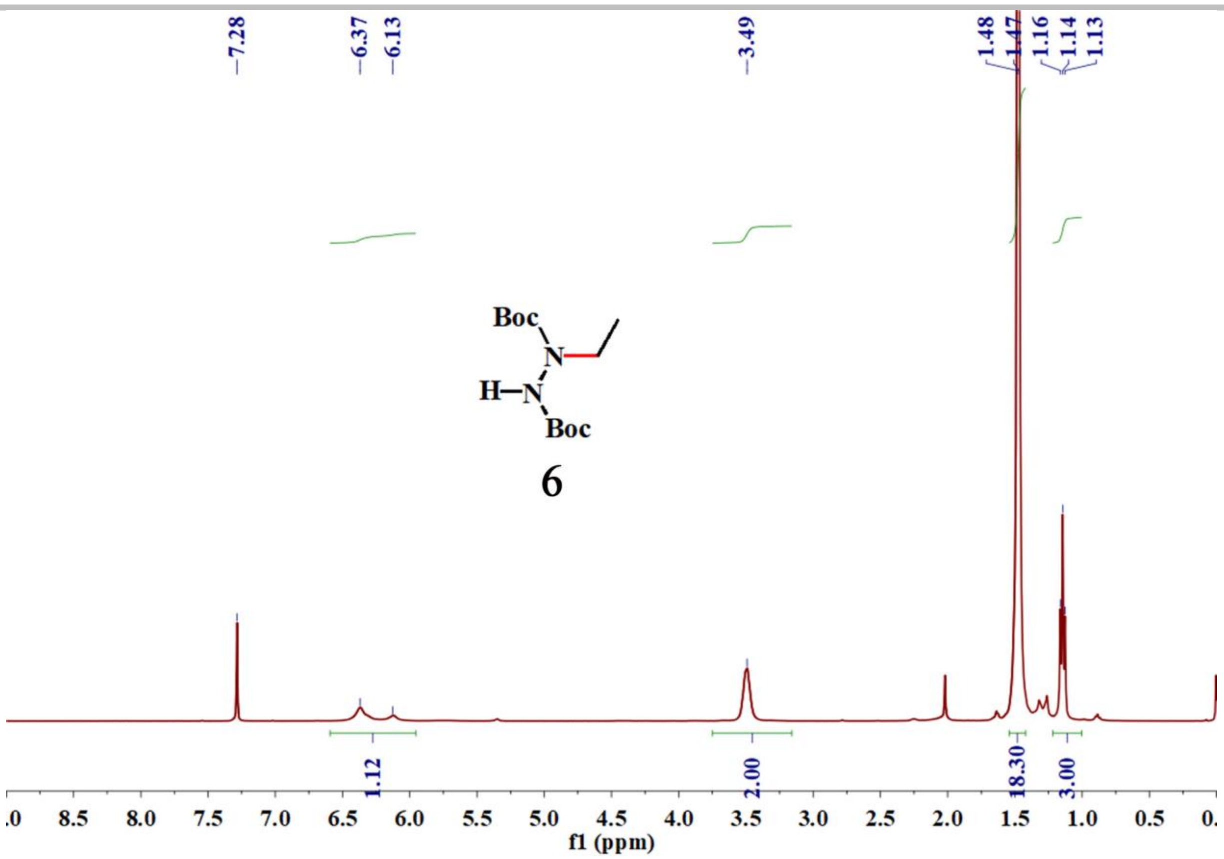

1H NMR (400 MHz, $CDCl_3$) δ 6.25 (d, *J* = 97.1 Hz, 1H), 3.49 (s, 2H), 1.48 (d, *J* = 4.8 Hz, 18H), 1.14 (t, *J* = 7.1 Hz, 3H).

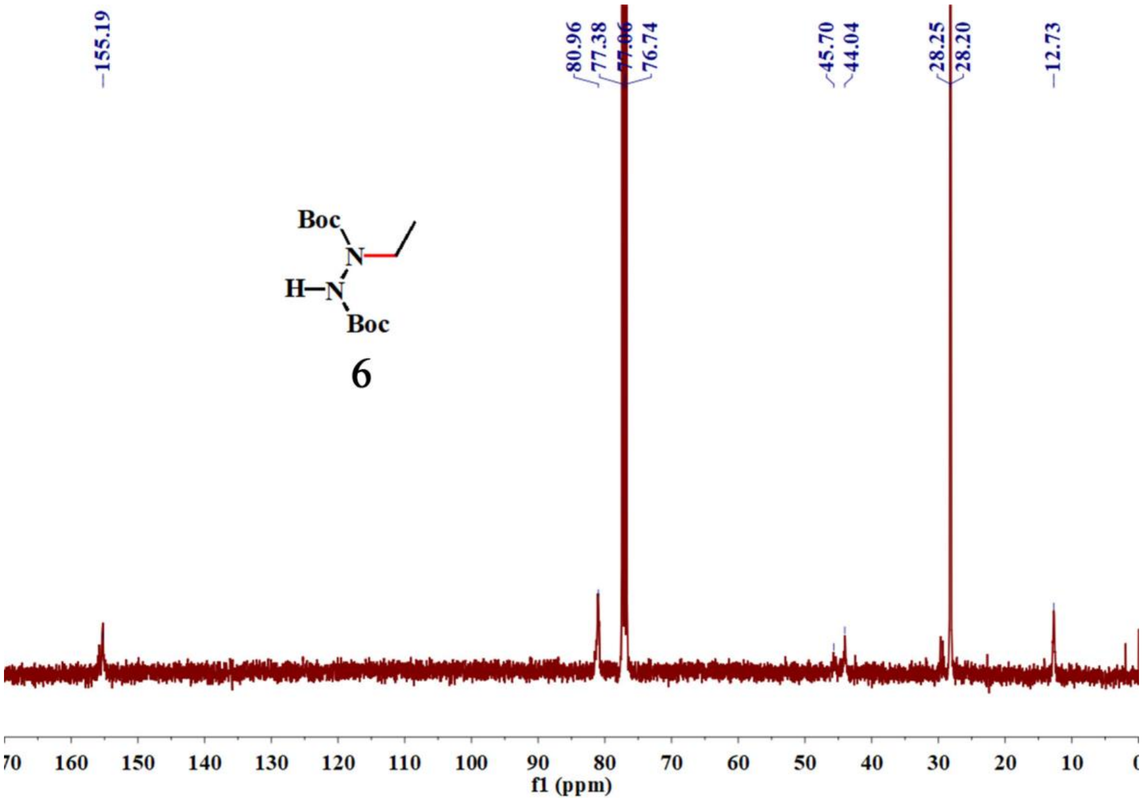

13C NMR (101 MHz, $CDCl_3$) δ 155.19, 80.96, 45.70, 44.04, 28.25, 28.20, 12.73.

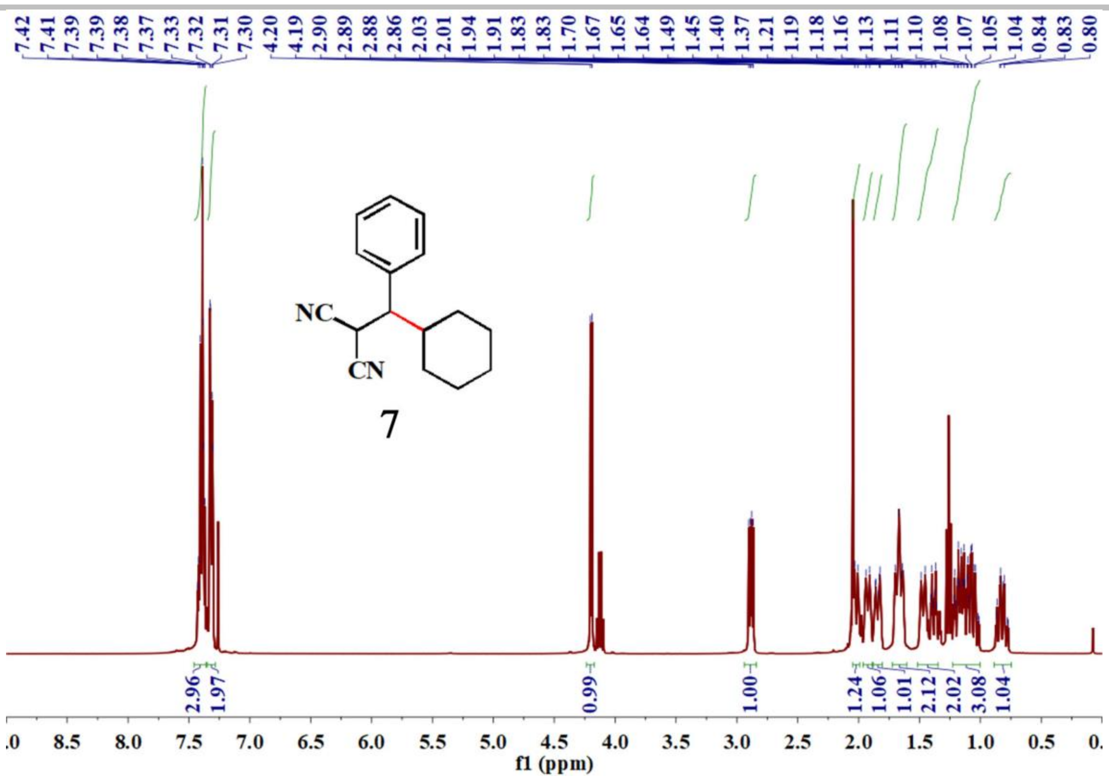

^{1}H NMR (400 MHz, $CDCl_3$) δ 7.46 – 7.36 (m, 3H), 7.32 (dd, *J* = 7.7, 1.7 Hz, 2H), 4.20 (d, *J* = 5.5 Hz, 1H), 2.88 (dd, *J* = 9.8, 5.5 Hz, 1H), 2.02 (d, *J* = 10.0 Hz, 1H), 1.92 (d, *J* = 12.3 Hz, 1H), 1.88 – 1.81 (m, 1H), 1.66 (dd, *J* = 16.8, 7.4 Hz, 2H), 1.40 (ddd, *J* = 19.3, 12.6, 8.3 Hz, 2H), 1.23 – 1.00 (m, 3H), 0.82 (ddd, *J* = 15.4, 12.3, 3.5 Hz, 1H).

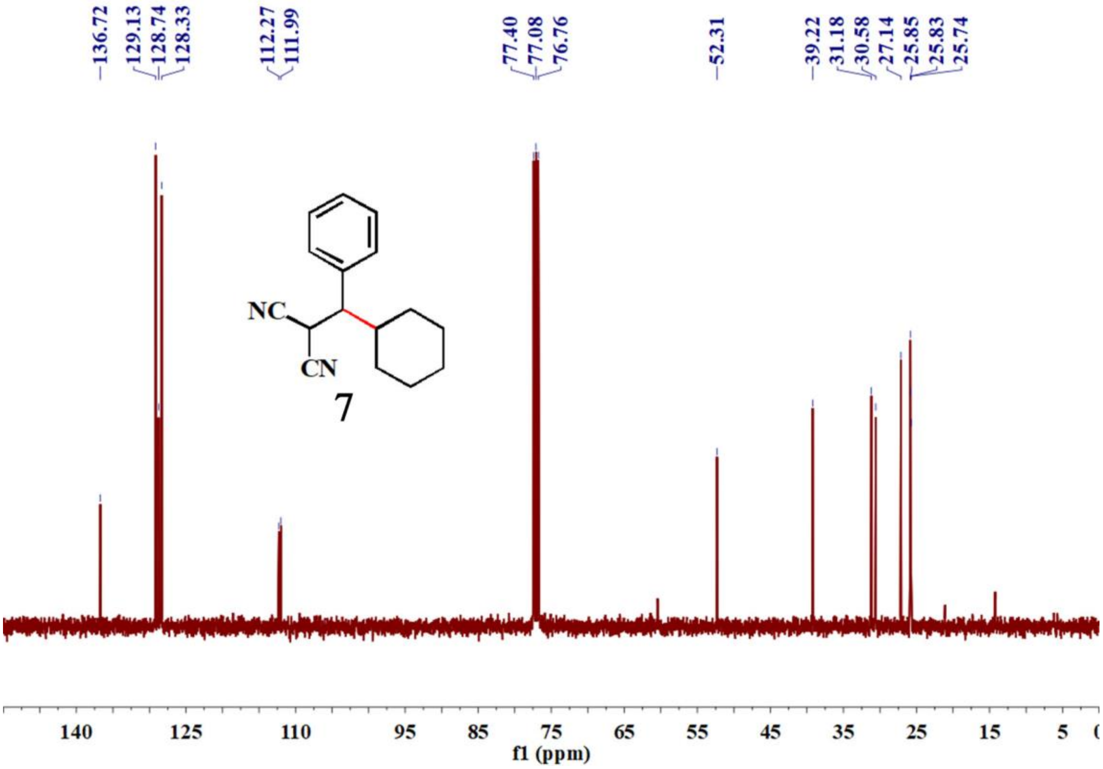

^{13}C NMR (101 MHz, $CDCl_3$) δ 136.72, 129.13, 128.74, 128.33, 112.27, 111.99, 52.31, 39.22, 31.18, 30.58, 27.14, 25.85, 25.83, 25.74.

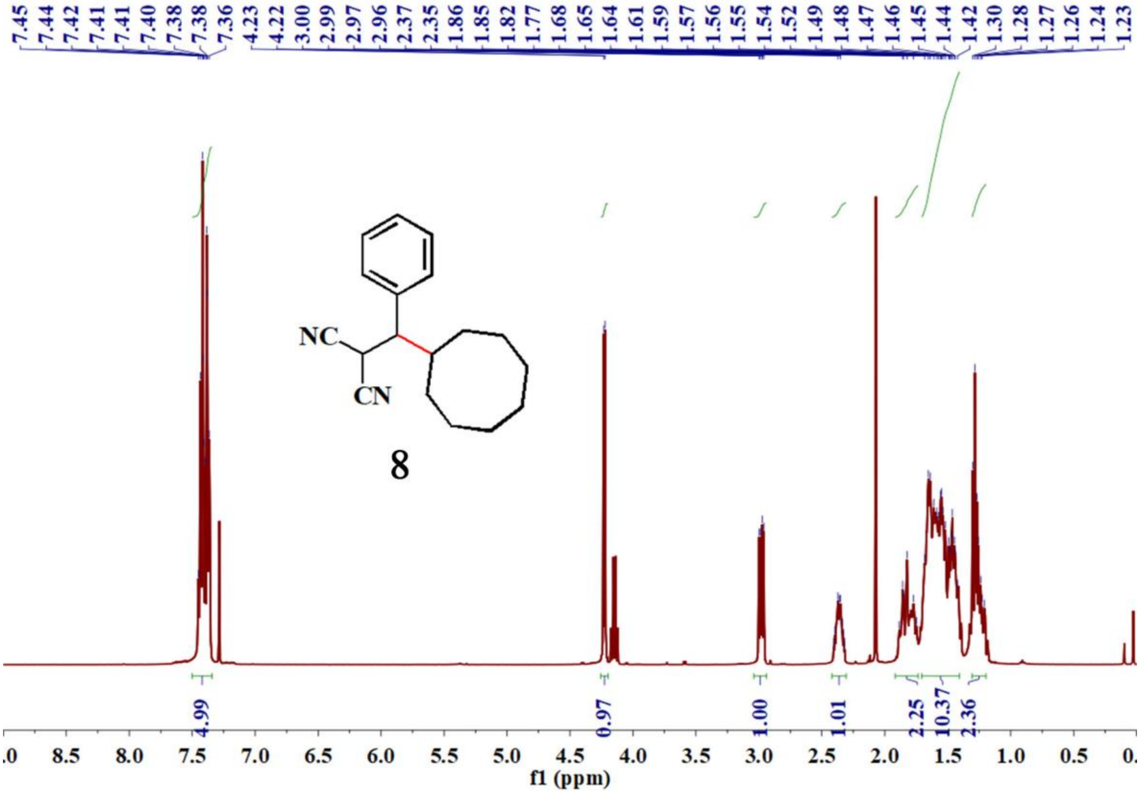

^{1}H NMR (400 MHz, $CDCl_3$) δ 7.50 – 7.34 (m, 5H), 4.23 (d, *J* = 5.3 Hz, 1H), 2.98 (dd, *J* = 10.3, 5.3 Hz, 1H), 2.42 – 2.31 (m, 1H), 1.82 (dt, *J* = 30.0, 10.0 Hz, 2H), 1.71 – 1.41 (m, 10H), 1.31 – 1.20 (m, 2H).

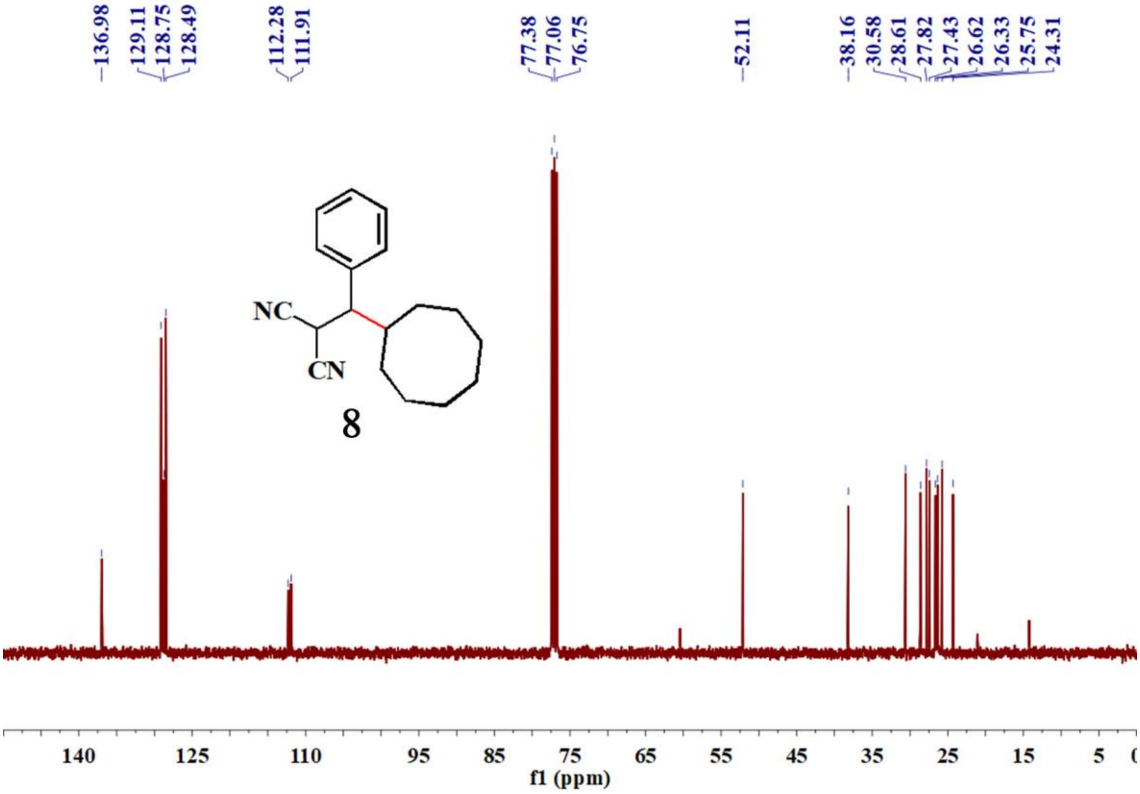

^{13}C NMR (101 MHz, $CDCl_3$) δ 136.98, 129.11, 128.75, 128.49, 112.28, 111.91, 52.11, 38.16, 30.58, 28.61, 27.82, 27.43, 26.62, 26.33, 25.75, 24.31.

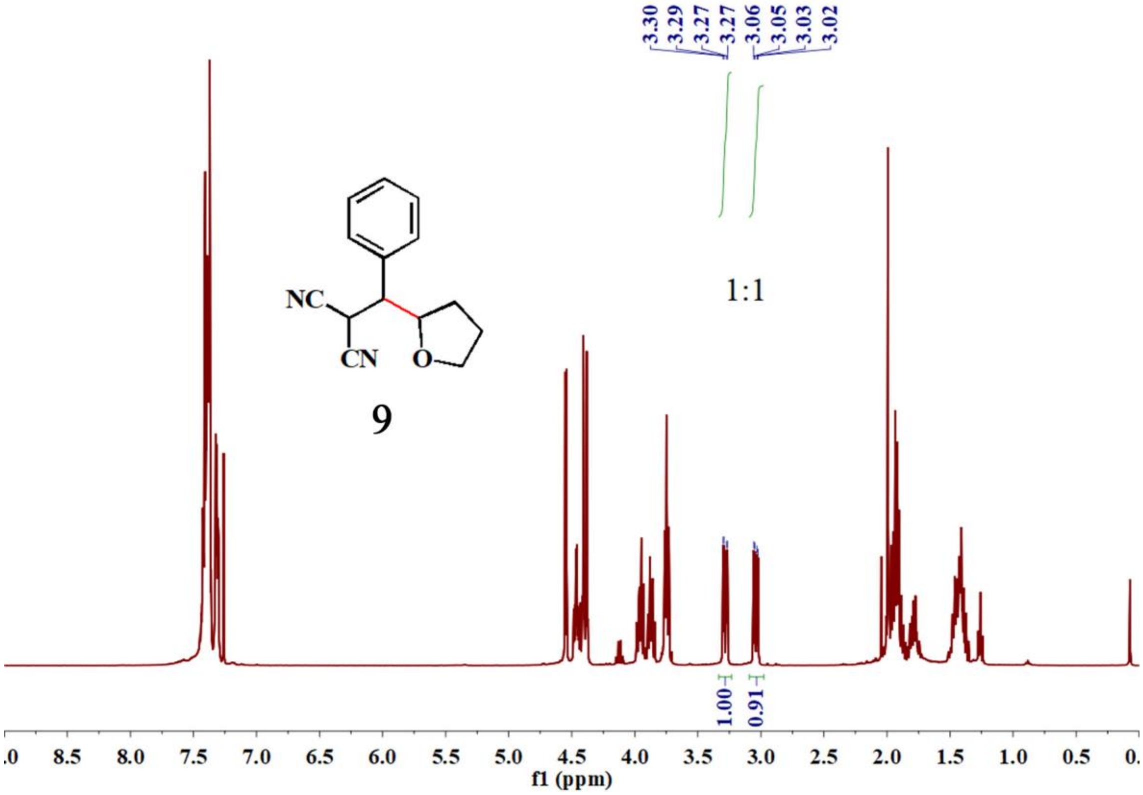

^{1}H NMR spectrum of crude product of 9.

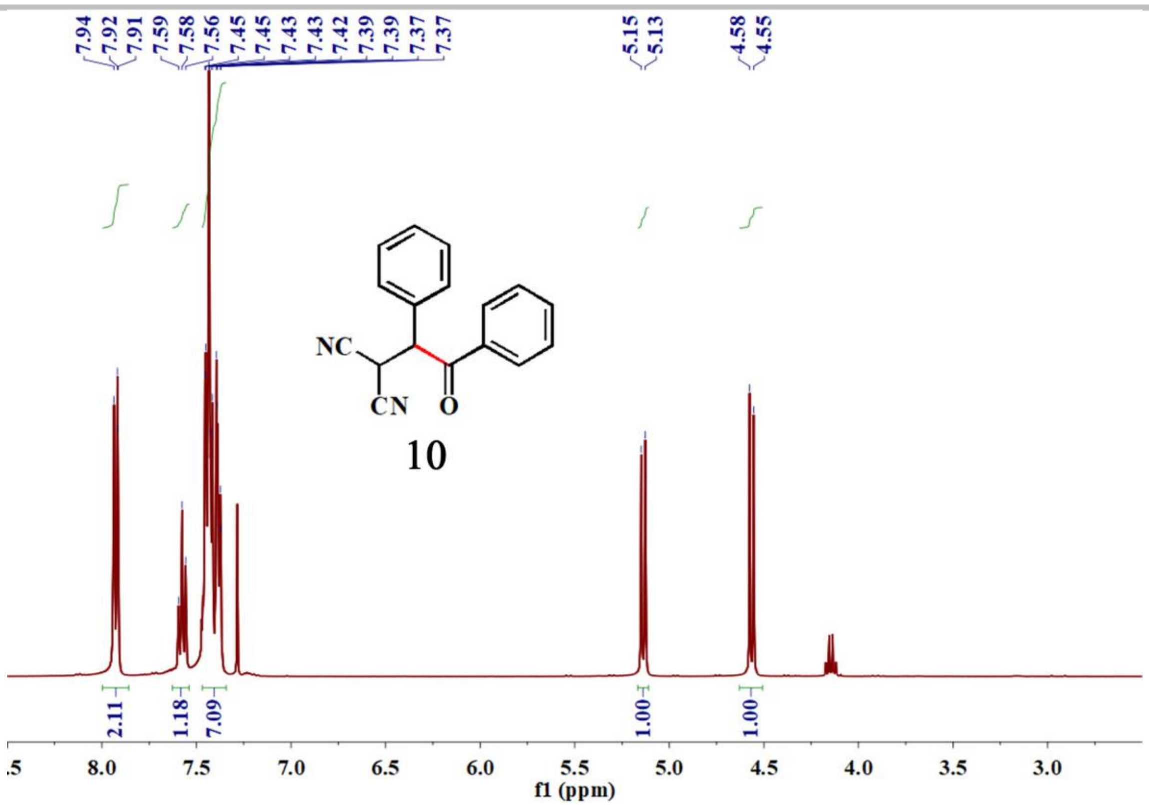

^{1}H NMR (400 MHz, $CDCl_3$) δ 8.00 – 7.86 (m, 2H), 7.58 (t, *J* = 7.4 Hz, 1H), 7.47 – 7.34 (m, 7H), 5.14 (d, *J* = 8.4 Hz, 1H), 4.56 (d, *J* = 8.4 Hz, 1H).

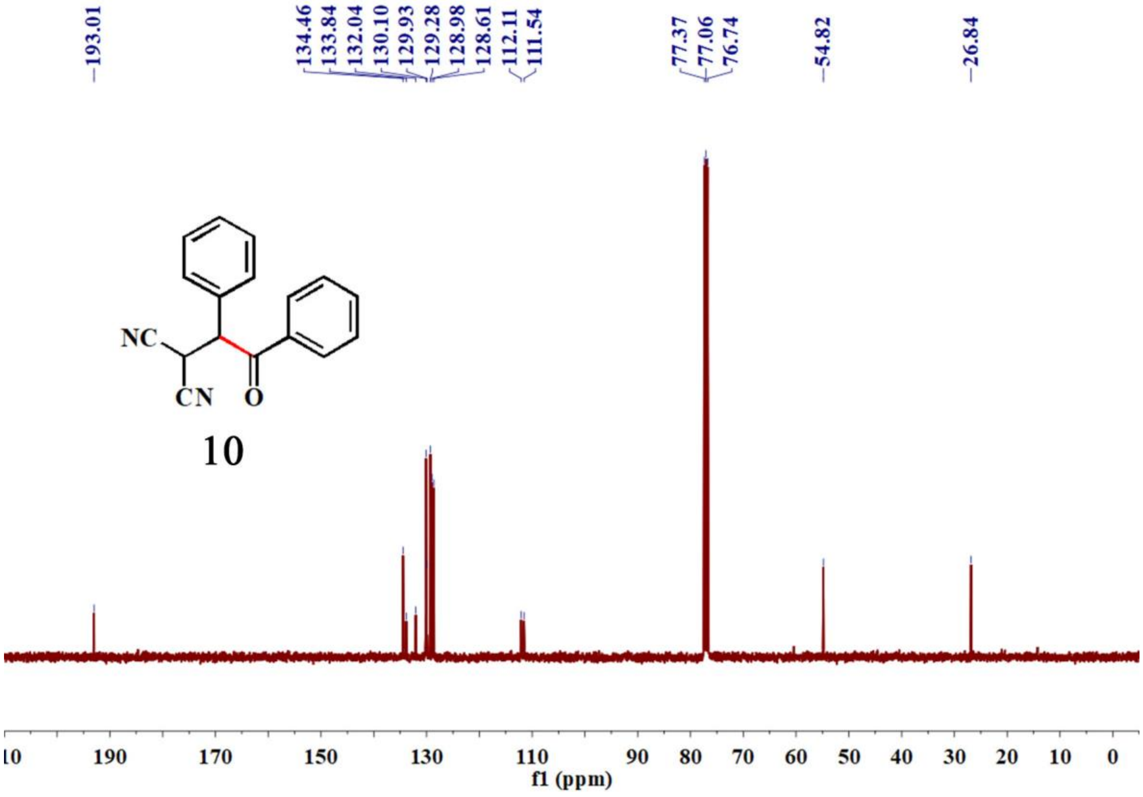

^{13}C NMR (101 MHz, $CDCl_3$) δ 193.01, 134.46, 133.84, 132.04, 130.10, 129.93, 129.28, 128.98, 128.61, 112.11, 111.54, 54.82, 26.84.

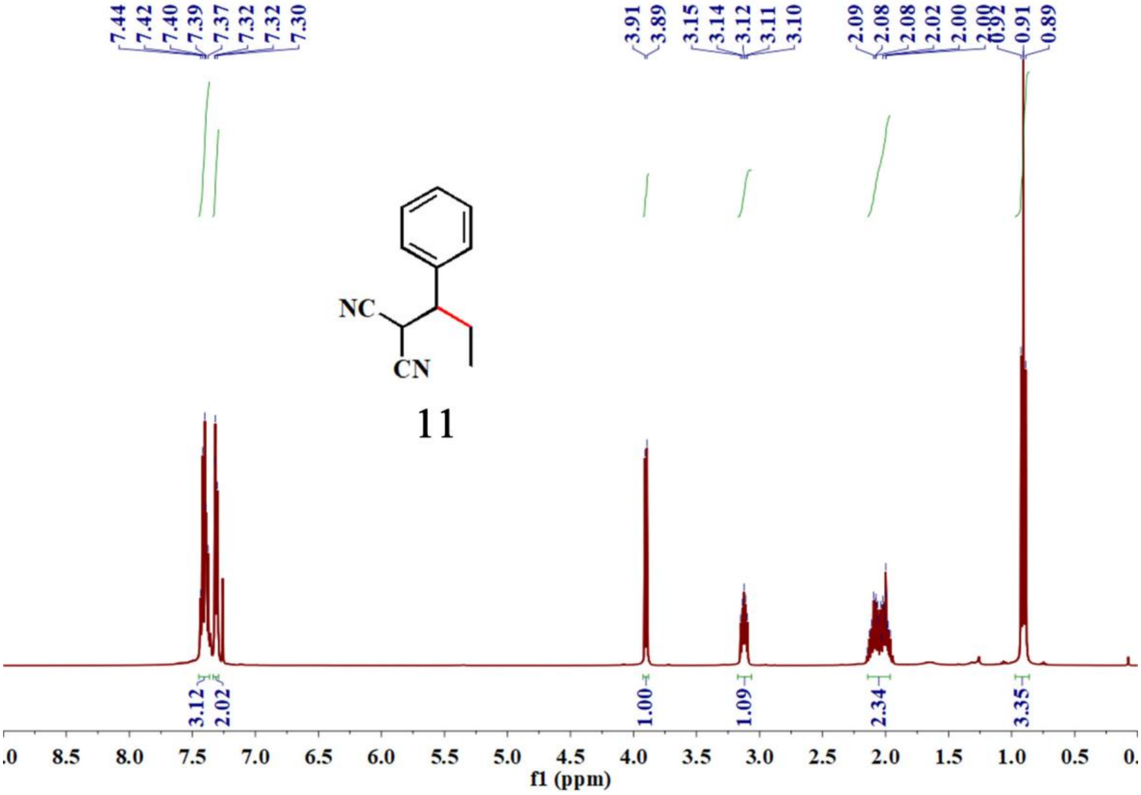

^{1}H NMR (400 MHz, CDCl3) δ 7.45 – 7.36 (m, 3H), 7.34 – 7.29 (m, 2H), 3.90 (d, J = 6.3 Hz, 1H), 3.17 – 3.06 (m, 1H), 2.14 – 1.96 (m, 2H), 0.91 (t, J = 7.3 Hz, 3H).

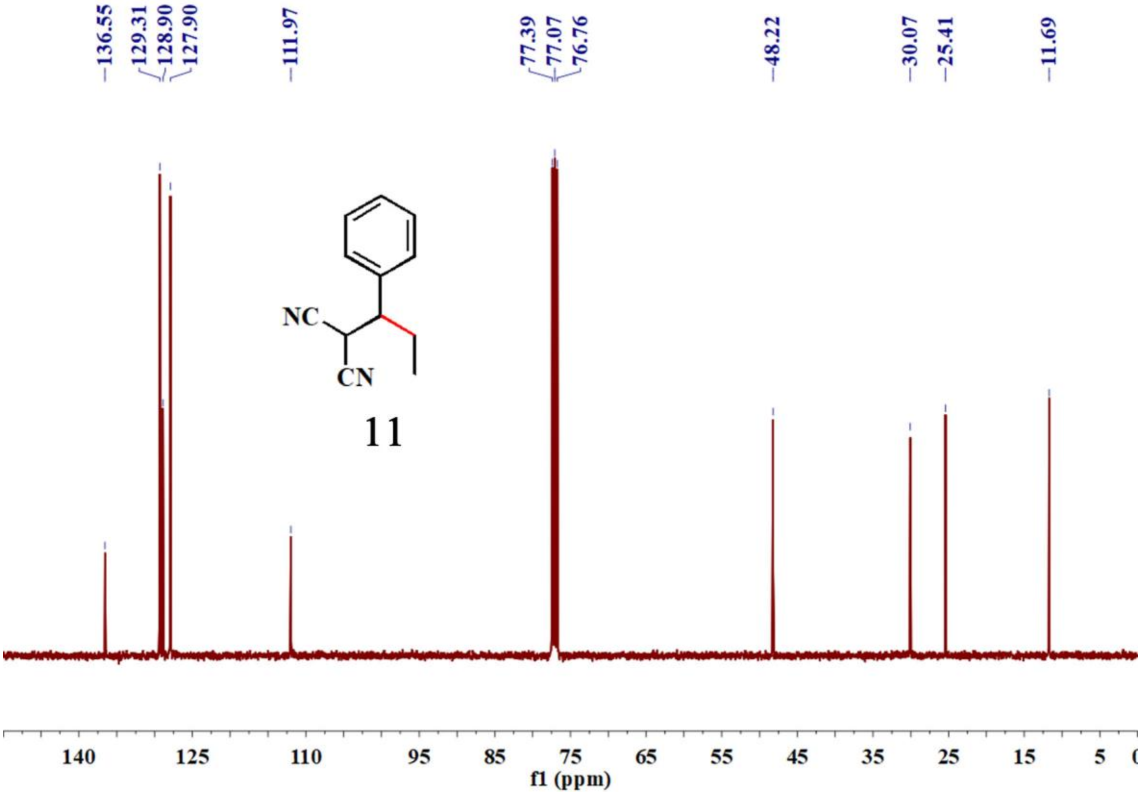

^{13}C NMR (101 MHz, $CDCl_3$) δ 136.55, 129.31, 128.90, 127.90, 111.97, 48.22, 30.07, 25.41, 11.69.

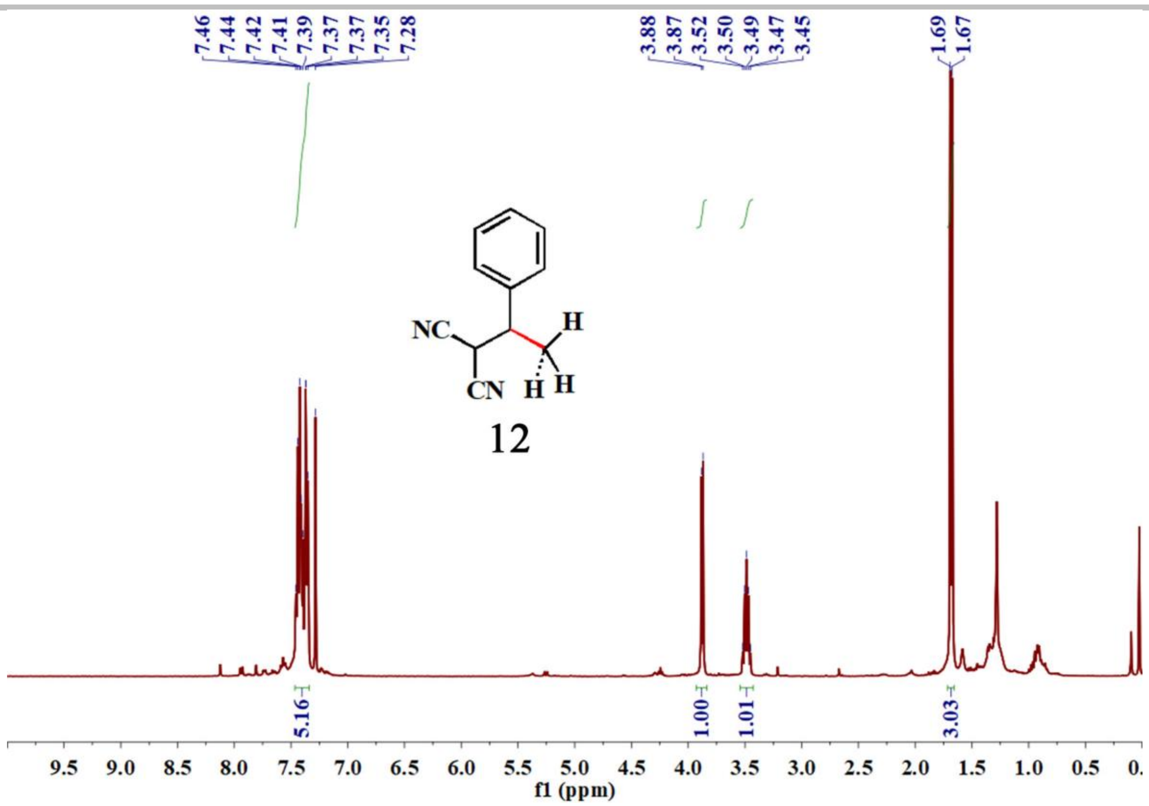

^{1}H NMR (400 MHz, $CDCl_3$) δ 7.40 (ddd, *J* = 17.1, 10.9, 6.7 Hz, 5H), 3.88 (d, *J* = 6.2 Hz, 1H), 3.49 (p, *J* = 6.9 Hz, 1H), 1.68 (d, *J* = 7.1 Hz, 3H).

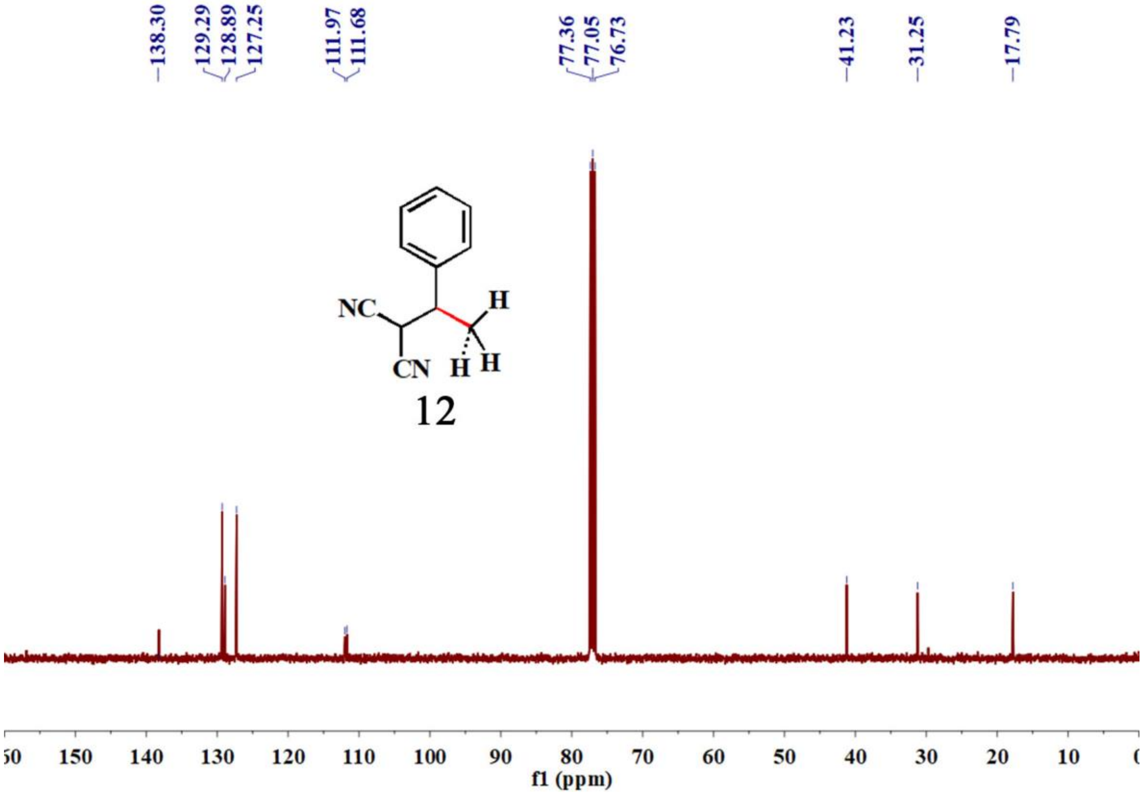

^{13}C NMR (101 MHz, $CDCl_3$) δ 138.30, 129.29, 128.89, 127.25, 111.97, 111.68, 41.23, 31.25, 17.79.

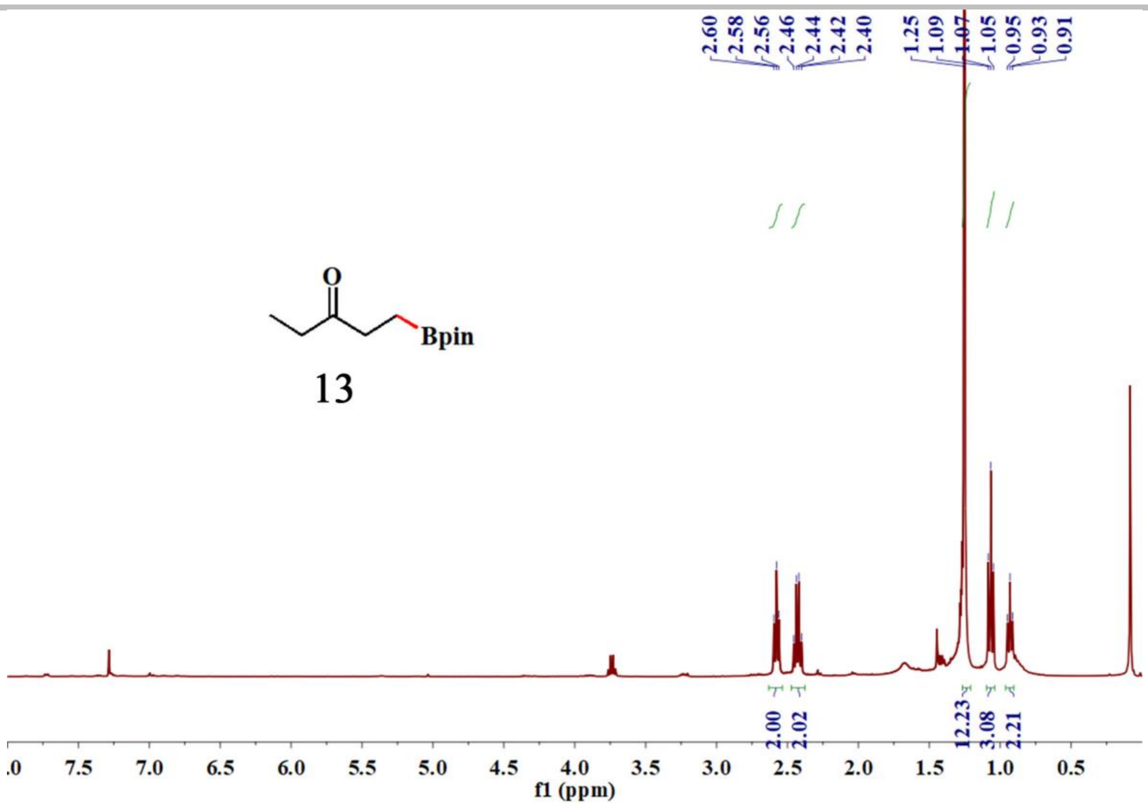

^{1}H NMR (400 MHz, $CDCl_3$) δ 2.58 (t, *J* = 7.1 Hz, 2H), 2.43 (q, *J* = 7.4 Hz, 2H), 1.25 (s, 12H), 1.07 (t, *J* = 7.4 Hz, 3H), 0.93 (t, *J* = 7.1 Hz, 2H).

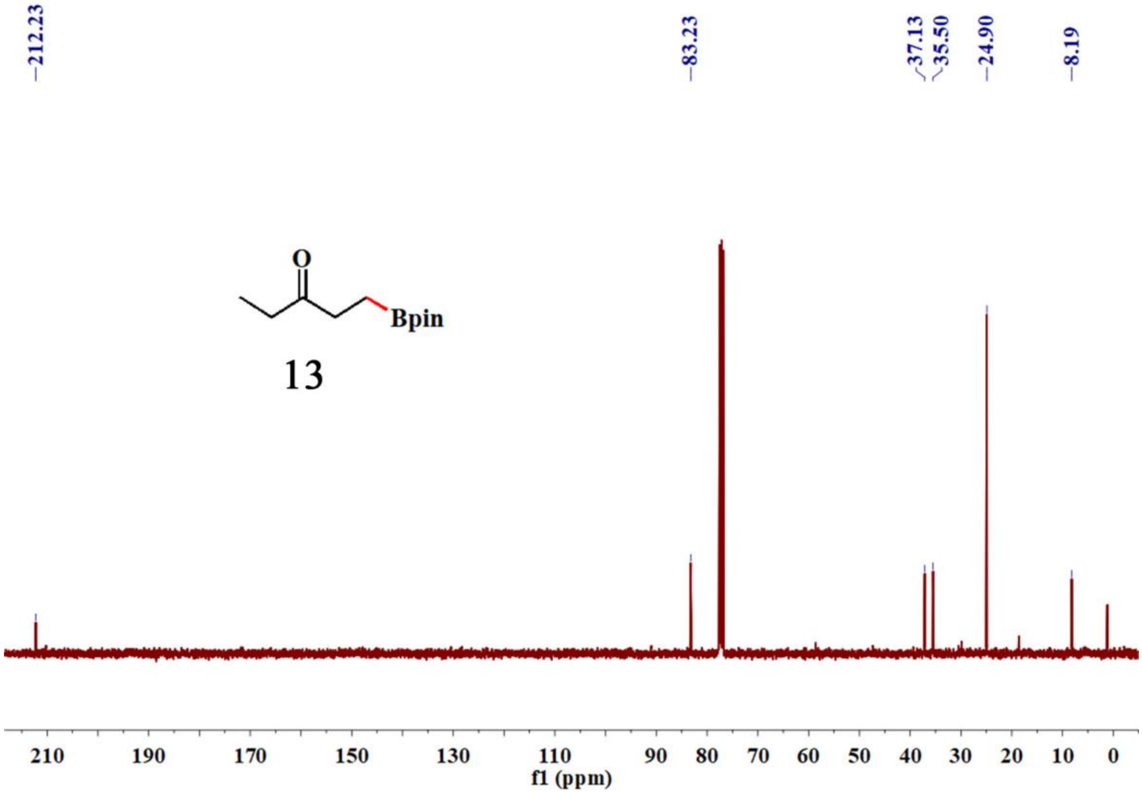

^{13}C NMR (101 MHz, $CDCl_3$) δ 212.23, 83.23, 37.13, 35.50, 24.90, 8.19.

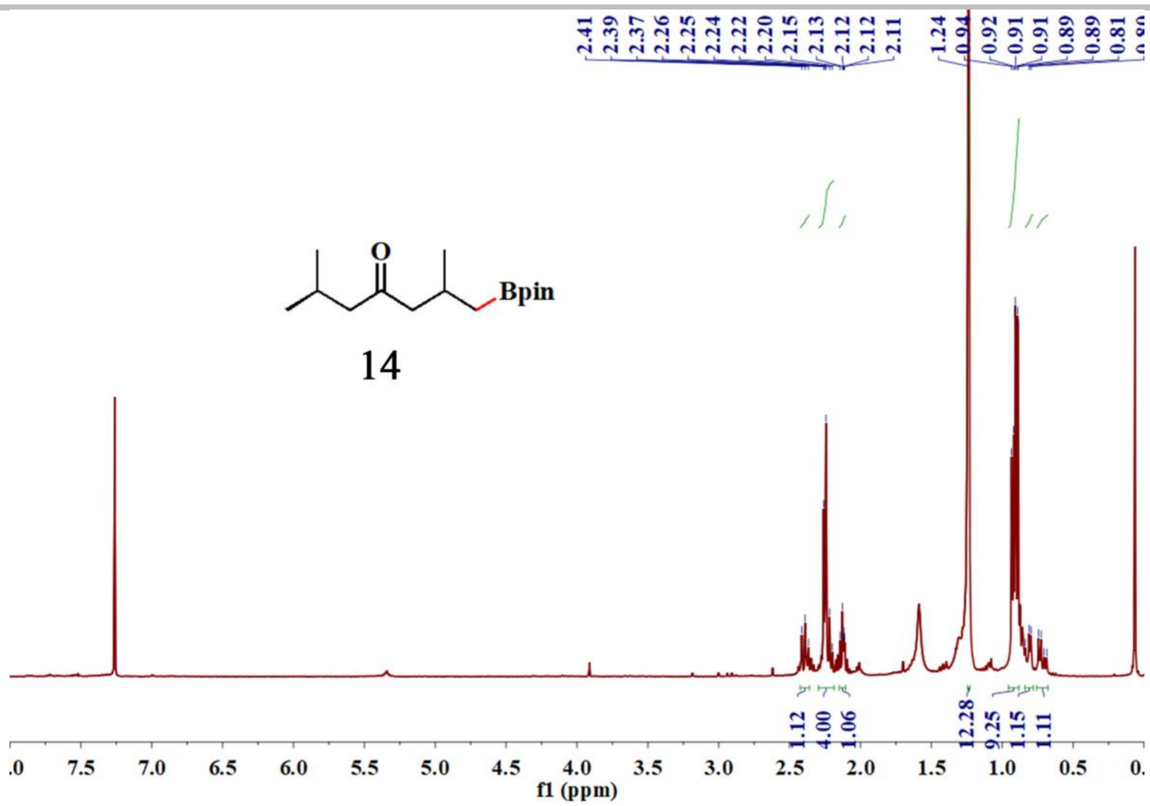

^{1}H NMR (400 MHz, $CDCl_3$) δ 2.39 (t, *J* = 9.5 Hz, 1H), 2.30-2.19 (m, 4H), 2.15-2.11 (m, 1H), 1.24 (s, 12H), 0.96-0.88 (m, 9H), 0.84-0.78 (m, 1H), 0.72 (dd, *J* = 15.6, 7.5 Hz, 1H).

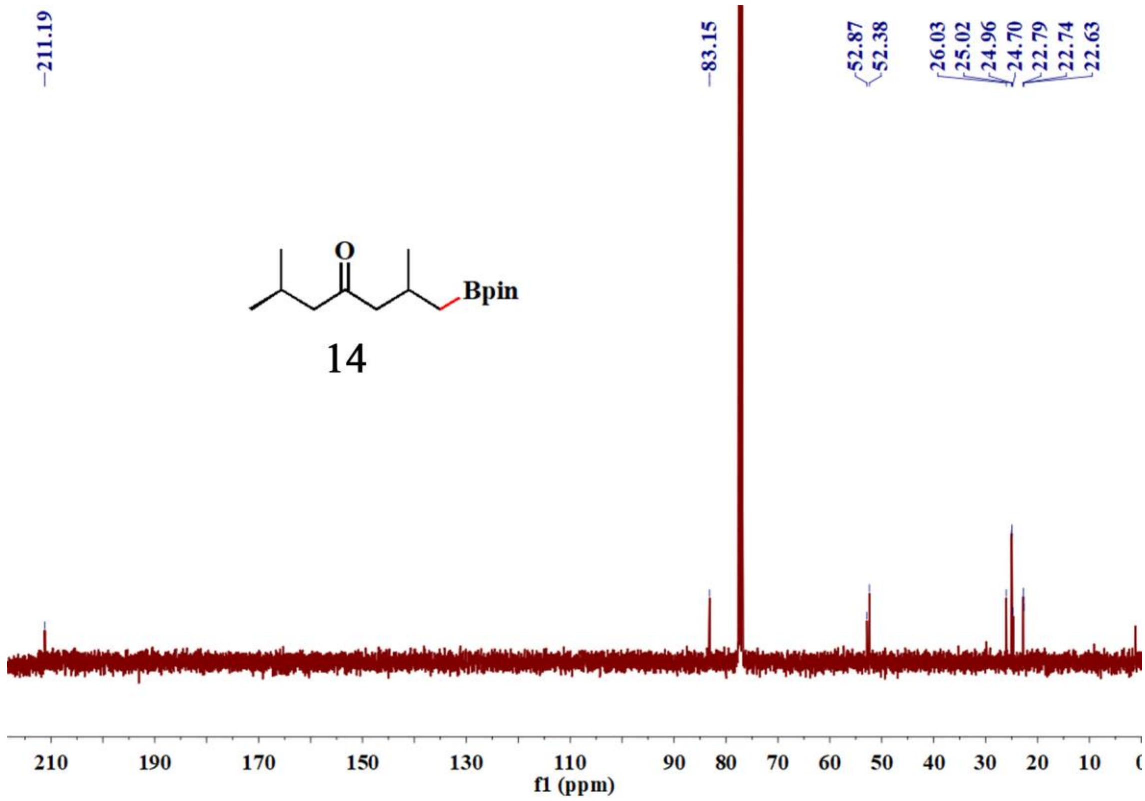

^{13}C NMR (101 MHz, $CDCl_3$) δ 211.19, 83.15, 52.87, 52.38, 26.03, 25.02, 24.96, 24.70, 22.79, 22.74, 22.63.

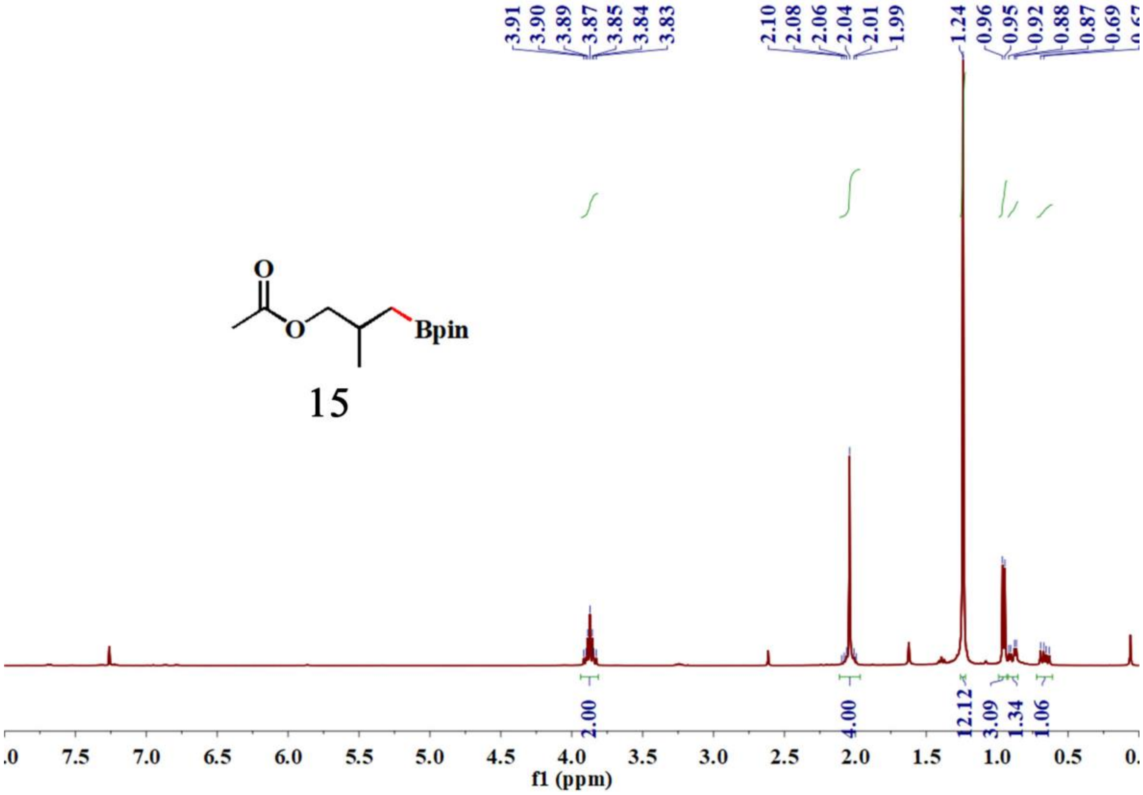

^{1}H NMR (400 MHz, $CDCl_3$) δ 3.94-3.81 (m, 2H), 2.11-1.97 (m, 4H), 1.24 (s, 12H), 0.96 (d, J = 6.7 Hz, 3H), 0.89 (dd, J = 15.7, 5.6 Hz, 1H), 0.66 (dd, J = 15.7, 8.7 Hz, 1H).

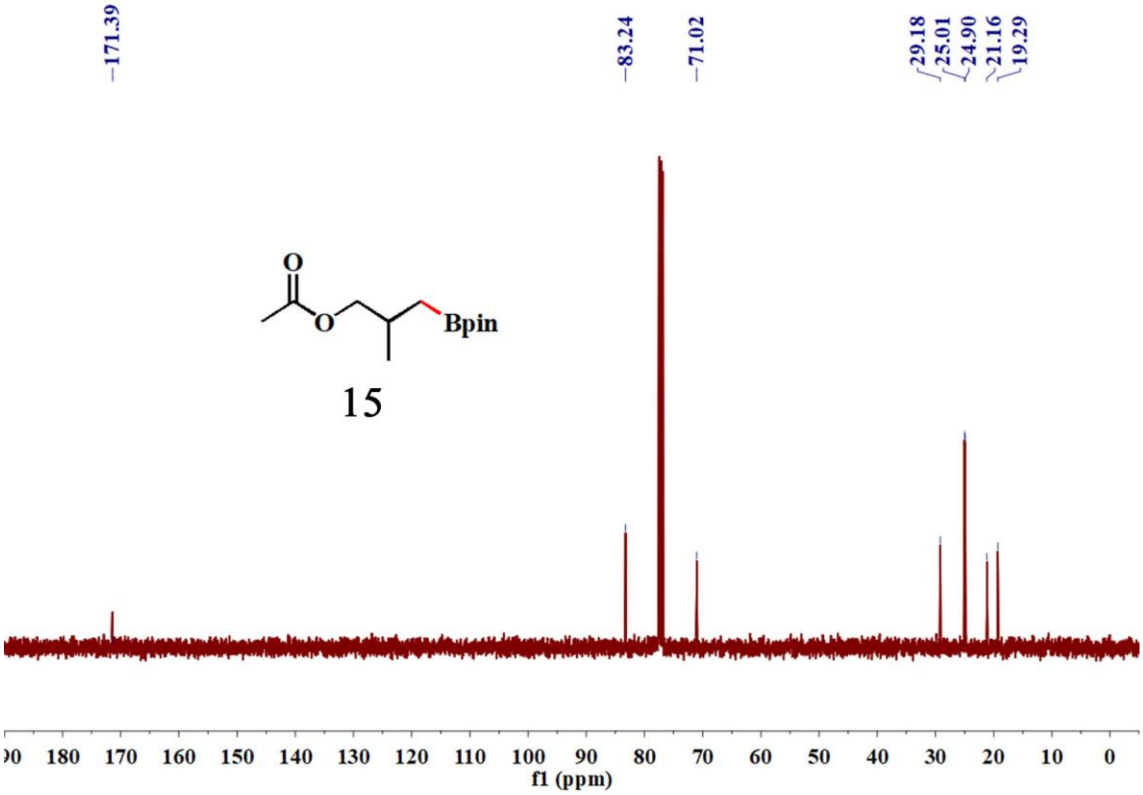

^{13}C NMR (101 MHz, $CDCl_3$) δ 171.39, 83.24, 71.02, 29.18,25.01, 24.90, 21.16, 19.29.

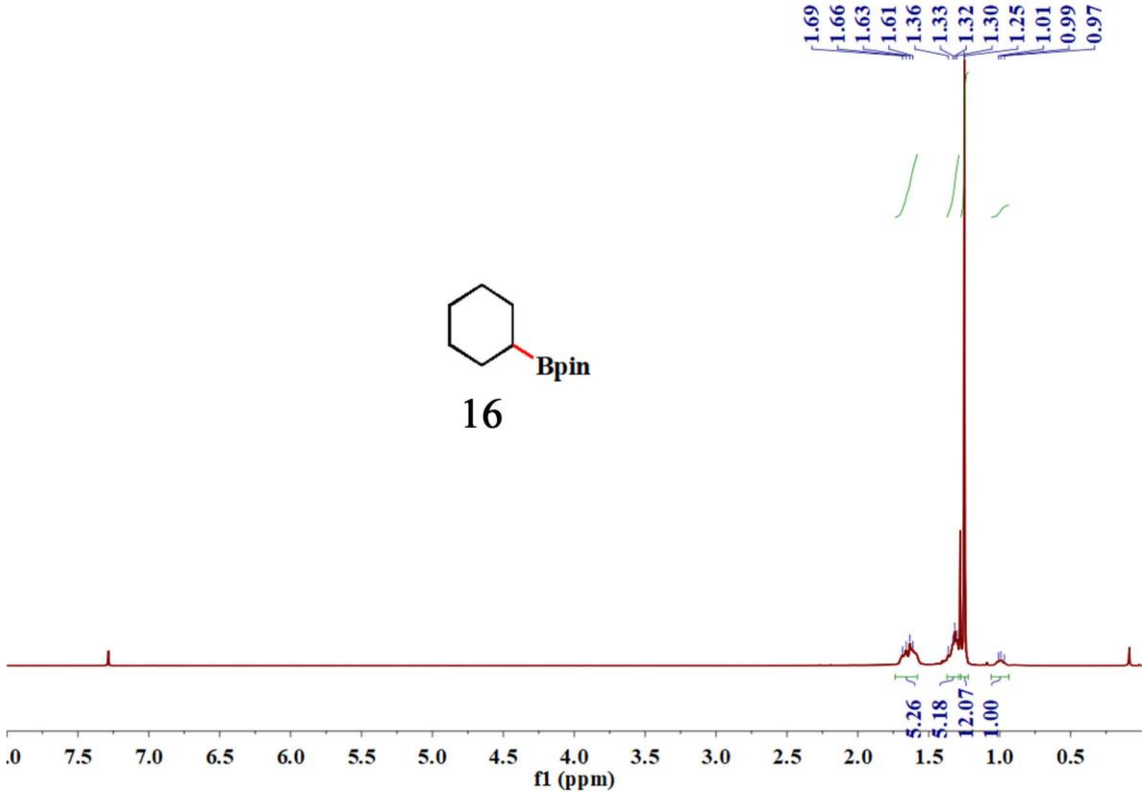

^{1}H NMR (400 MHz, $CDCl_3$) δ 1.65 (dd, *J* = 20.0, 9.4 Hz, 5H), 1.33 (dd, *J* = 15.2, 9.8 Hz, 5H), 1.25 (s, 12H), 0.99 (t, *J* = 8.2 Hz, 1H).

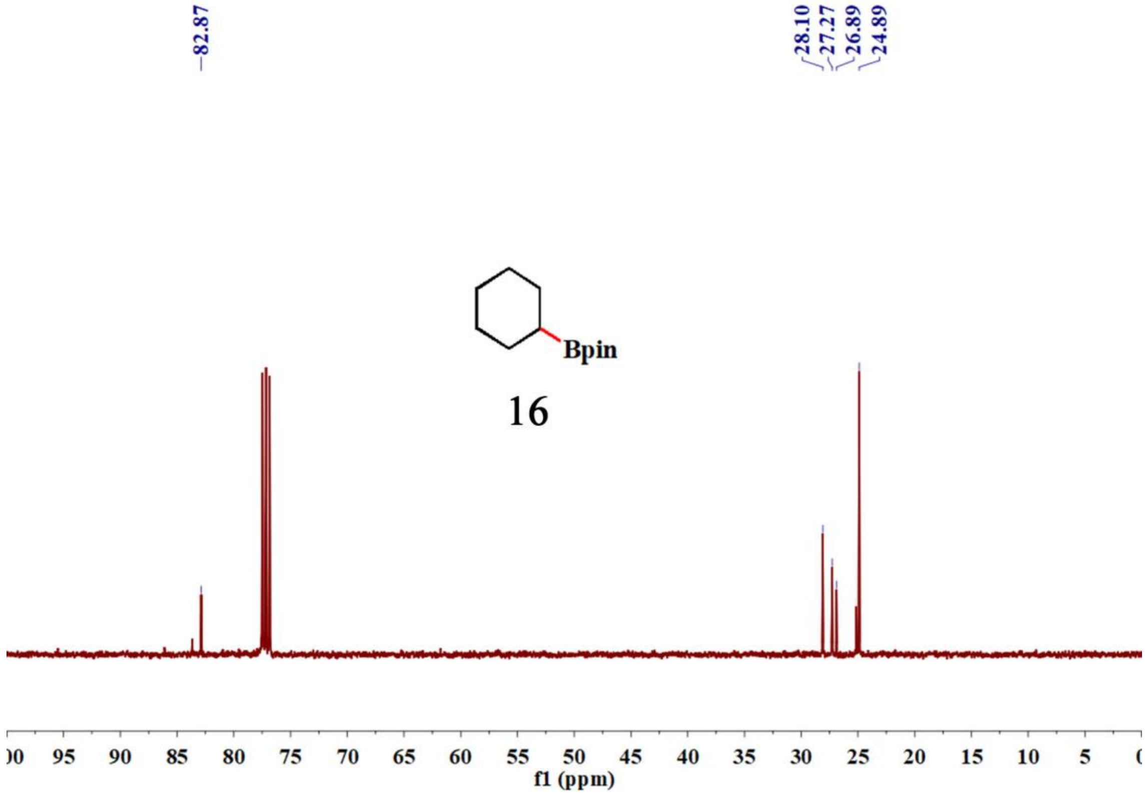

^{13}C NMR (101 MHz, $CDCl_3$) δ 82.87, 28.10, 27.27, 26.89, 24.89.

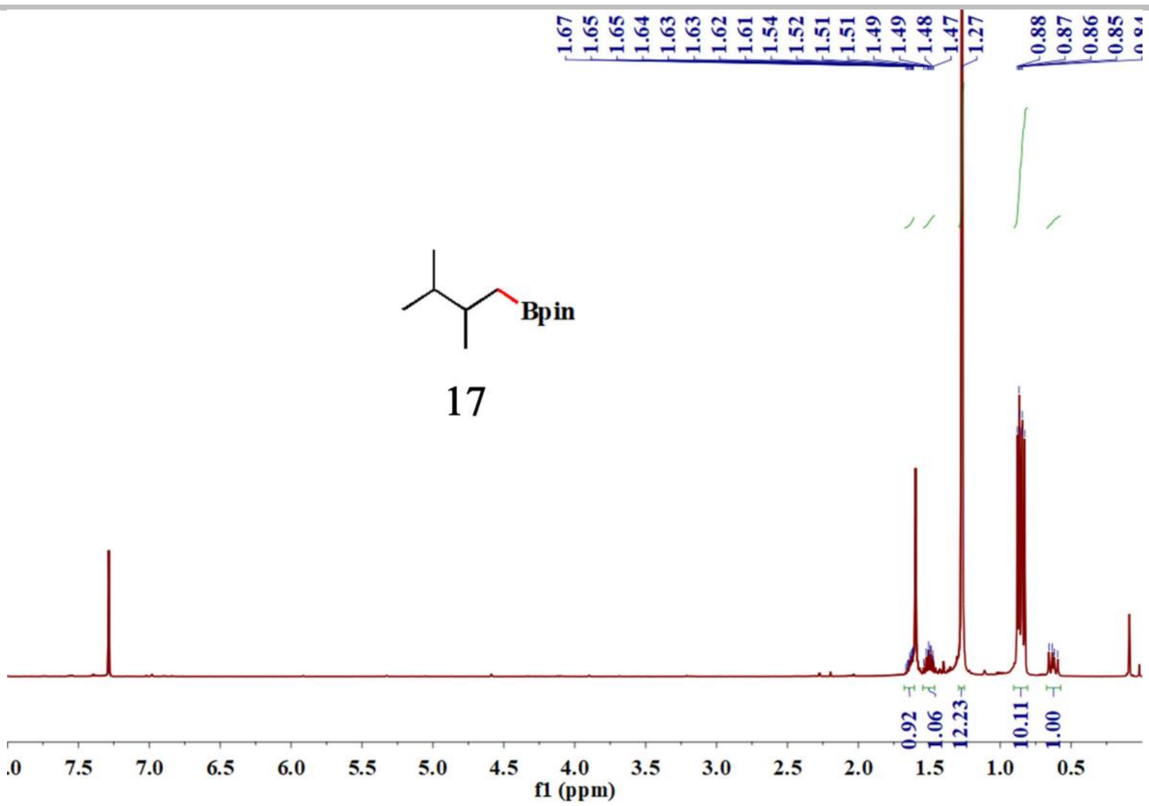

^1H NMR (400 MHz, $CDCl_3$) δ 1.64 (ddd, *J* = 8.3, 6.0, 4.2 Hz, 1H), 1.50 (ddd, *J* = 9.3, 8.5, 4.2 Hz, 1H), 1.27 (s, 12H), 0.90 – 0.81 (m, 10H), 0.63 (dd, *J* = 15.3, 9.8 Hz, 1H).

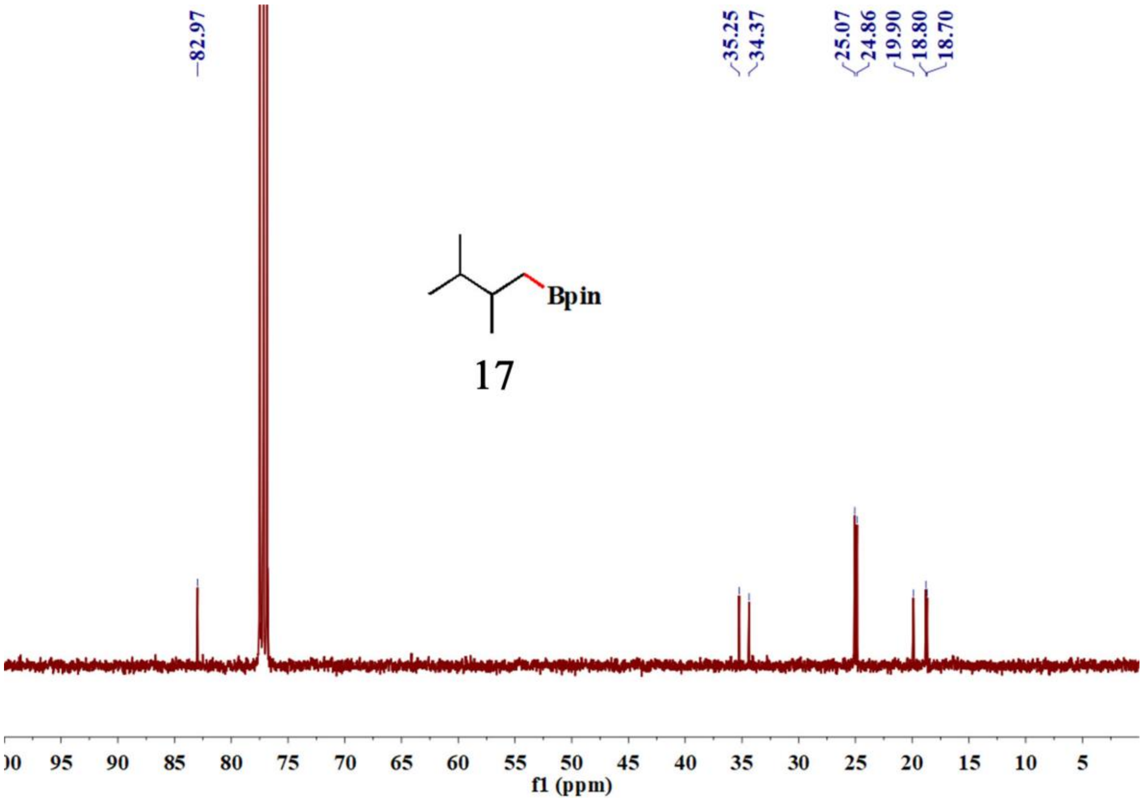

^{13}C NMR (101 MHz, $CDCl_3$) δ 82.97, 35.25, 34.37, 25.07, 24.86, 19.90, 18.80, 18.70.

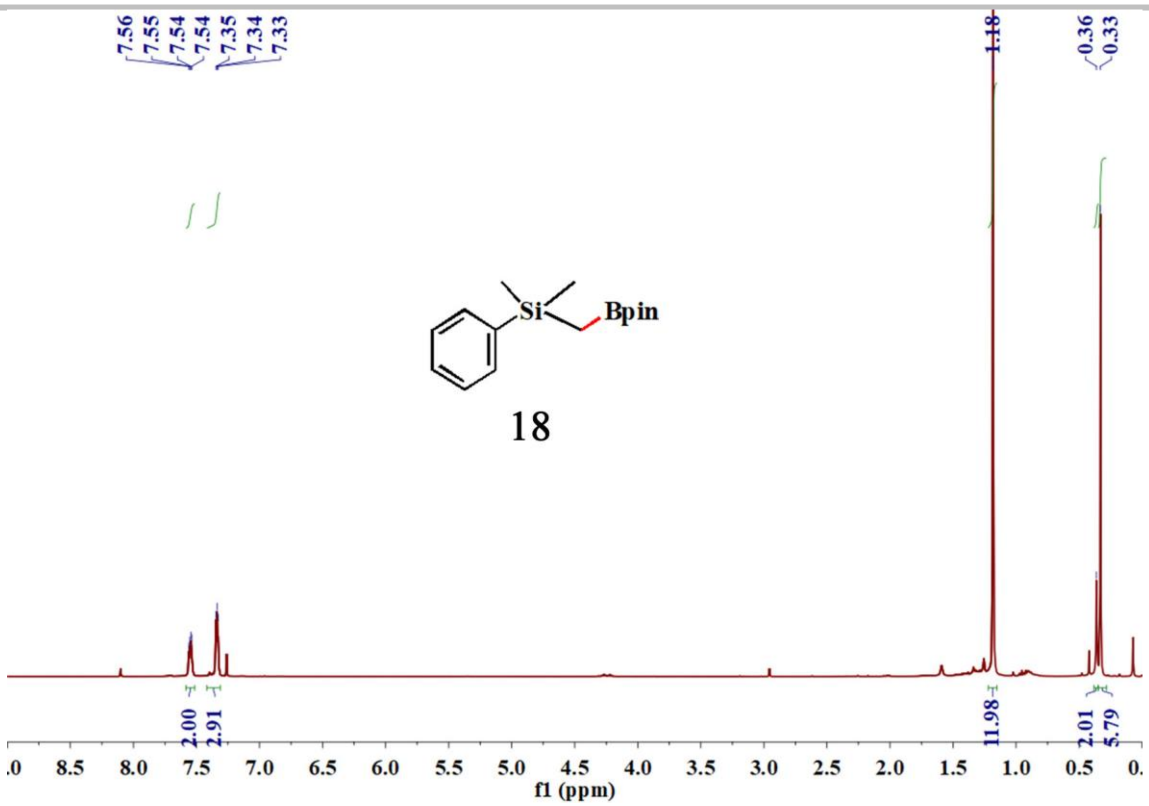

^1H NMR (400 MHz, $CDCl_3$) δ 7.55 (dd, J = 6.5, 2.9 Hz, 2H), 7.42 – 7.31 (m, 3H), 1.18 (s, 12H), 0.36 (s, 2H), 0.33 (s, 6H).

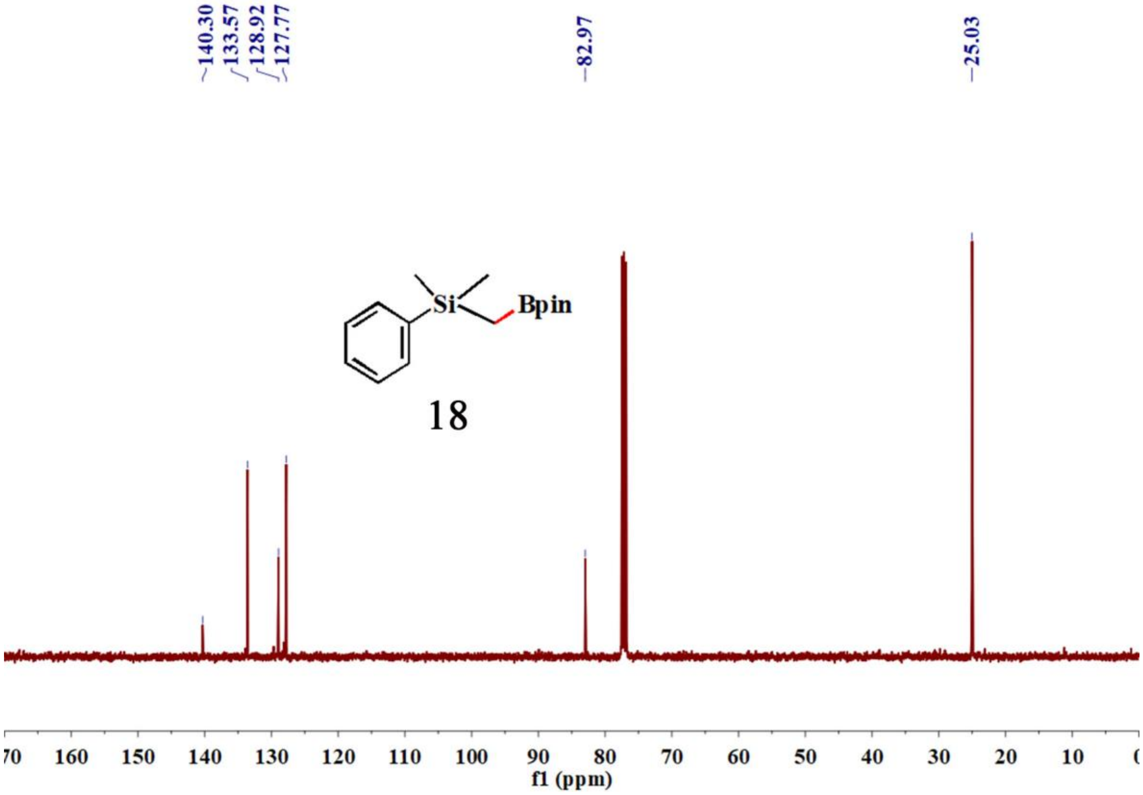

^{13}C NMR (101 MHz, $CDCl_3$) δ 140.30, 133.57, 128.92, 127.77, 82.97, 25.03.

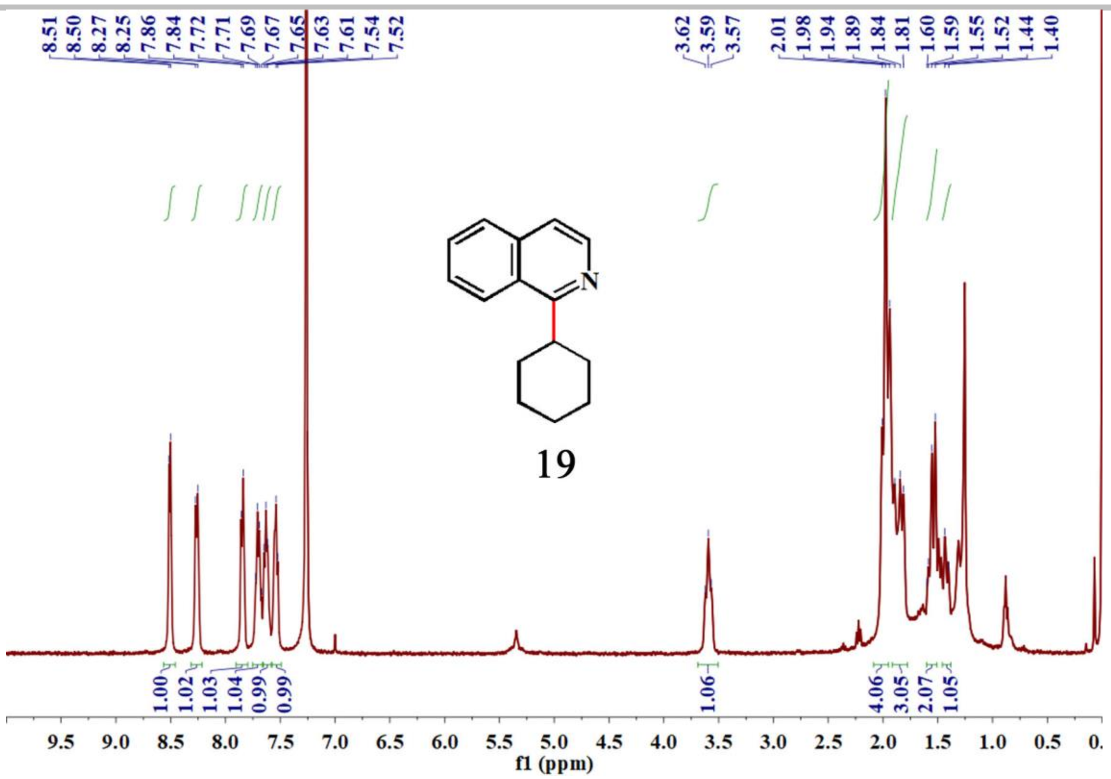

^1H NMR (400 MHz, $CDCl_3$) δ 8.51 (d, *J* = 5.5 Hz, 1H), 8.26 (d, *J* = 8.3 Hz, 1H), 7.85 (d, *J* = 7.8 Hz, 1H), 7.70 (dd, *J* = 13.6, 7.1 Hz, 1H), 7.65 – 7.58 (m, 1H), 7.53 (d, *J* = 6.7 Hz, 1H), 3.59 (t, *J* = 11.2 Hz, 1H), 1.99 (d, *J* = 12.7 Hz, 4H), 1.92 – 1.78 (m, 3H), 1.57 (dd, *J* = 21.6, 8.3 Hz, 2H), 1.42 (d, *J* = 14.6 Hz, 1H).

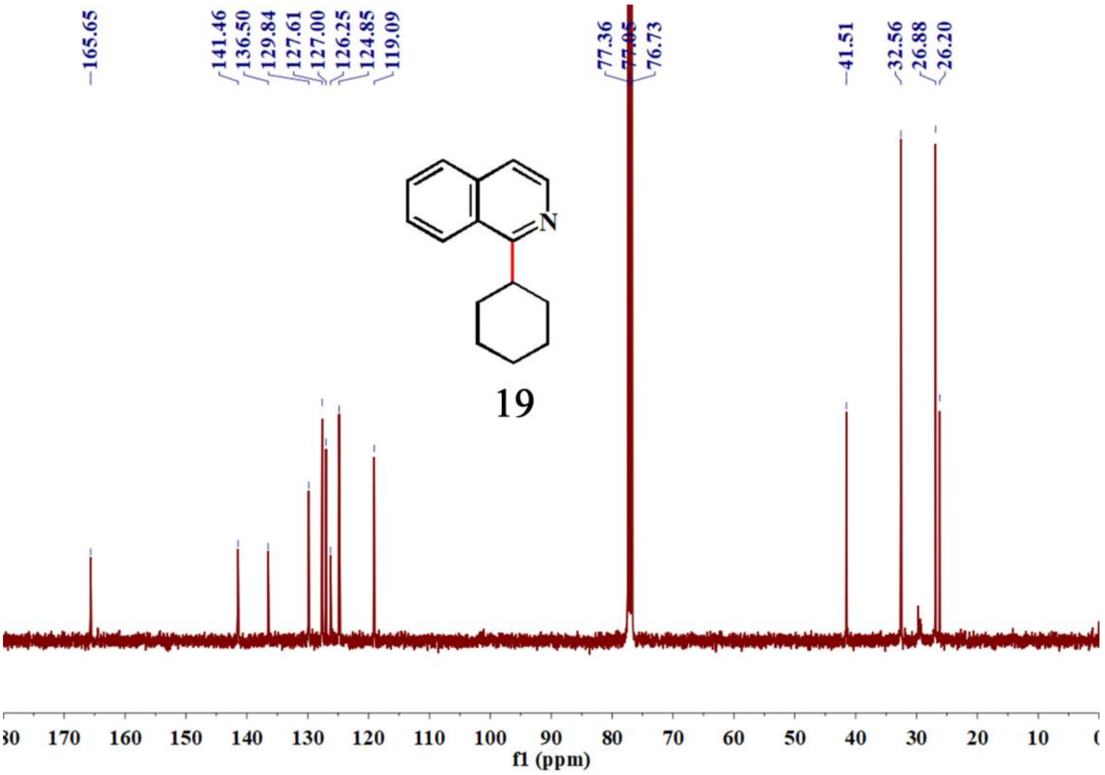

^{13}C NMR (101 MHz, $CDCl_3$) δ 165.65, 141.46, 136.50, 129.84, 127.61, 127.00, 126.25, 124.85, 119.09, 41.51, 32.56, 26.88, 26.20.

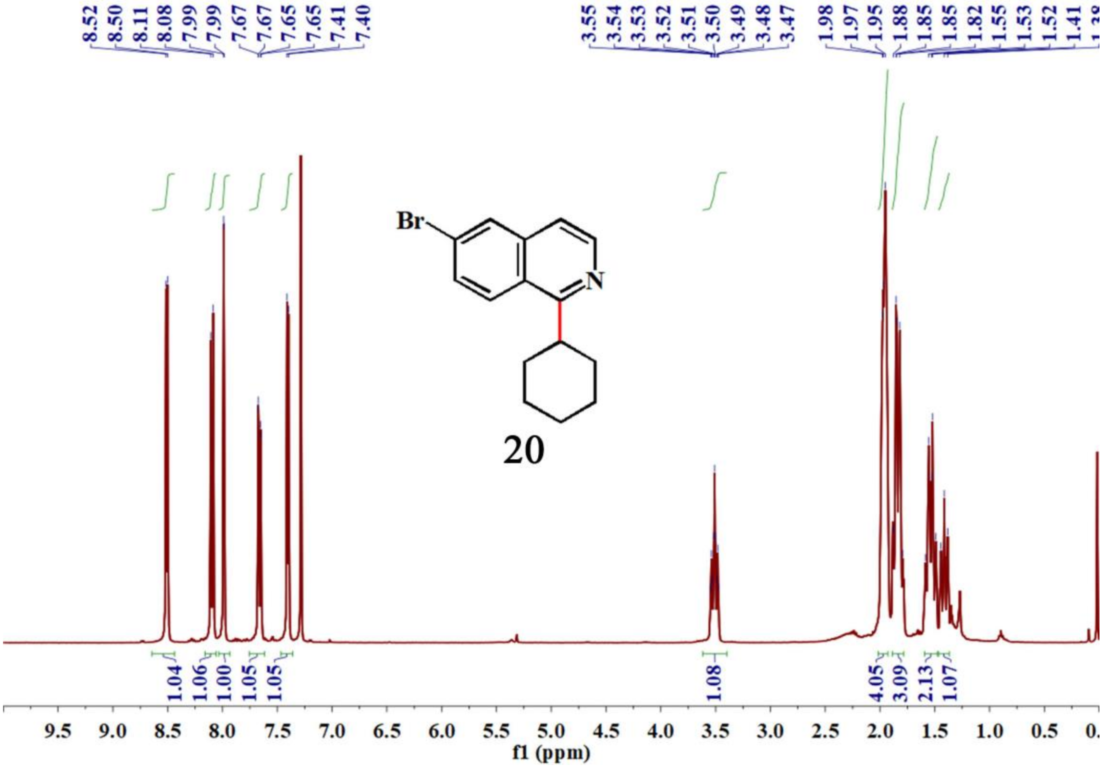

^1H NMR (400 MHz, $CDCl_3$) δ 8.51 (d, *J* = 5.5 Hz, 1H), 8.26 (d, *J* = 8.3 Hz, 1H), 7.85 (d, *J* = 7.8 Hz, 1H), 7.70 (dd, *J* = 13.6, 7.1 Hz, 1H), 7.65 – 7.58 (m, 1H), 7.53 (d, *J* = 6.7 Hz, 1H), 3.59 (t, *J* = 11.2 Hz, 1H), 1.99 (d, *J* = 12.7 Hz, 4H), 1.92 – 1.78 (m, 3H), 1.57 (dd, *J* = 21.6, 8.3 Hz, 2H), 1.42 (d, *J* = 14.6 Hz, 1H).

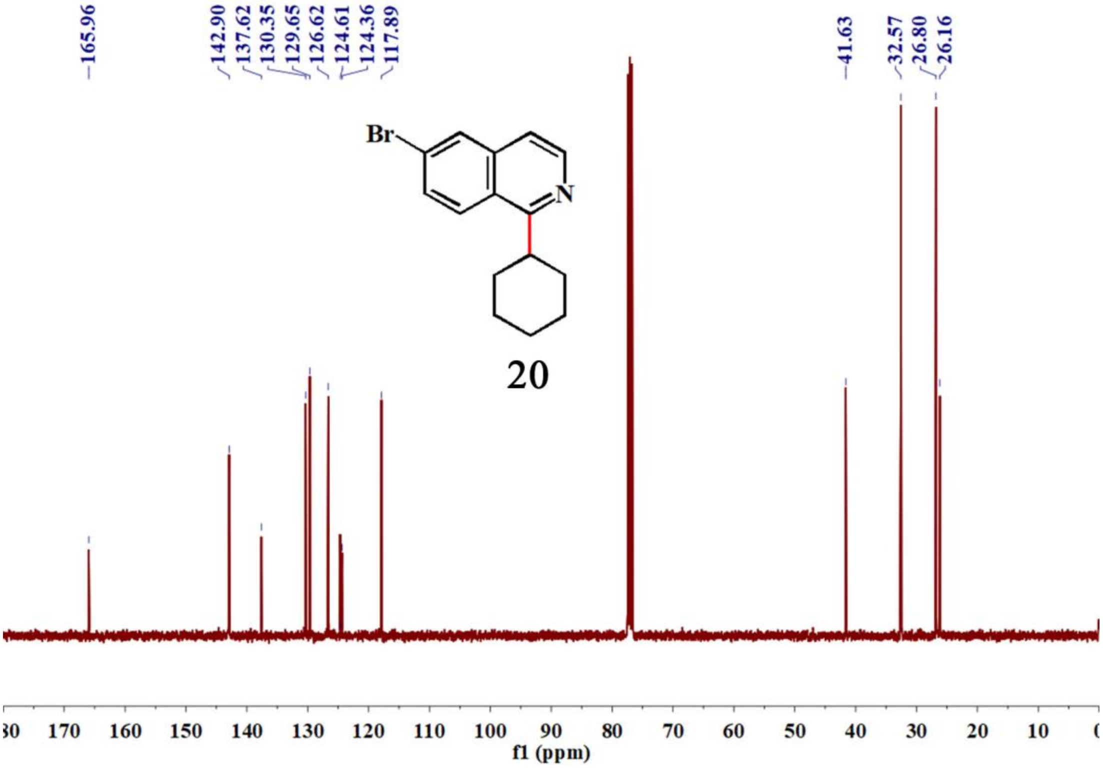

^{13}C NMR (101 MHz, $CDCl_3$) δ 165.96, 142.90, 137.62, 130.35, 129.65, 126.62, 124.61, 124.36, 117.89, 41.63, 32.57, 26.80, 26.16.

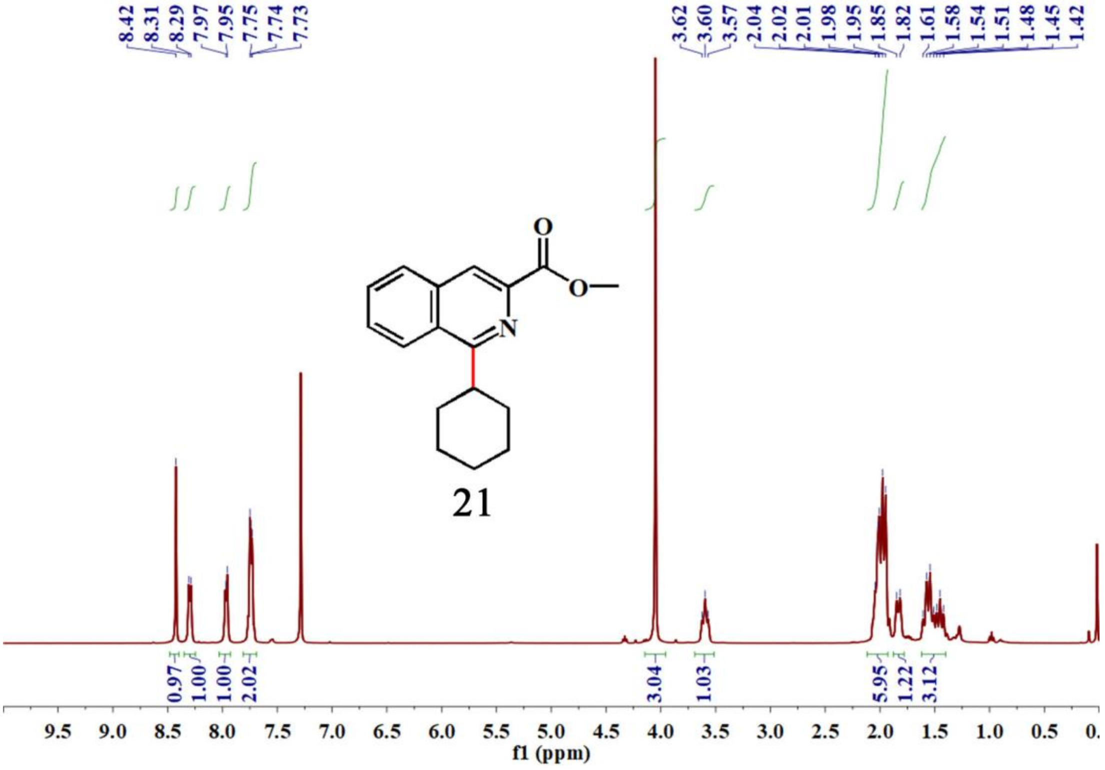

^{1}H NMR (400 MHz, $CDCl_3$) δ 8.42 (s, 1H), 8.30 (d, *J* = 8.5 Hz, 1H), 7.96 (d, *J* = 5.8 Hz, 1H), 7.81 – 7.69 (m, 2H), 4.05 (s, 3H), 3.60 (t, *J* = 11.0 Hz, 1H), 2.12 – 1.93 (m, 6H), 1.83 (d, *J* = 12.1 Hz, 1H), 1.62 – 1.40 (m, 3H).

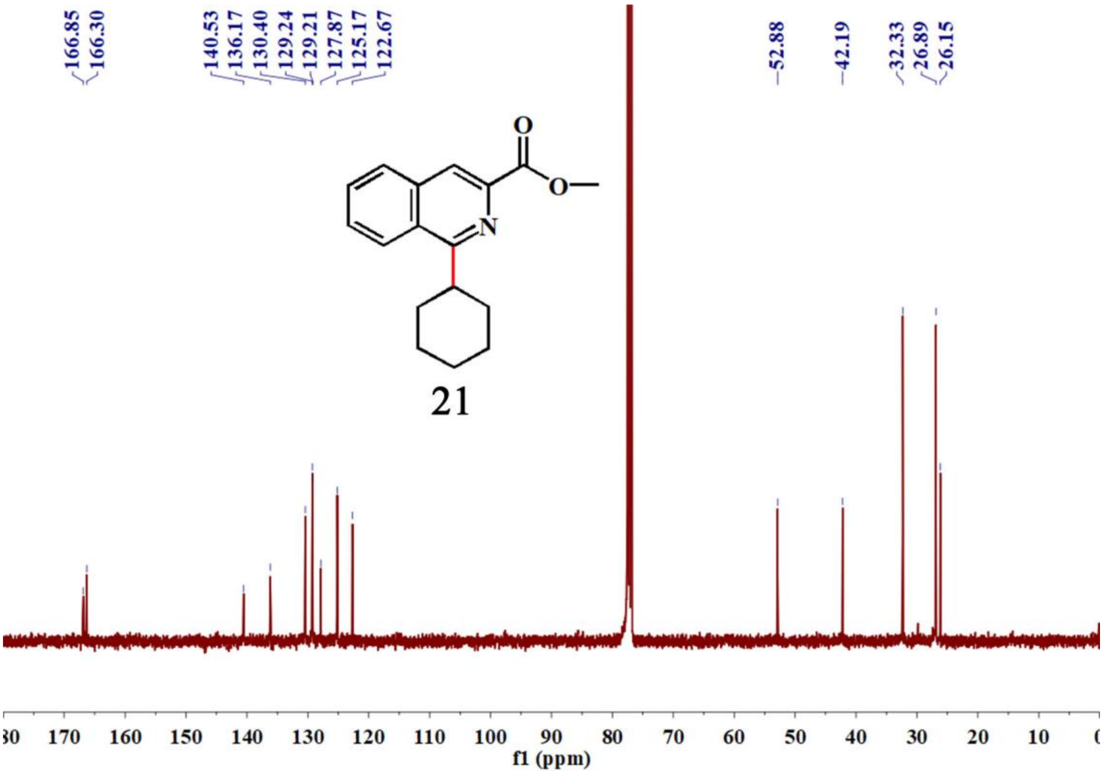

^{13}C NMR (101 MHz, $CDCl_3$) δ 166.85, 166.30, 140.53, 136.17, 130.40, 129.24, 129.21, 127.87, 125.17, 122.67, 52.88, 42.19, 32.33, 26.89, 26.15.

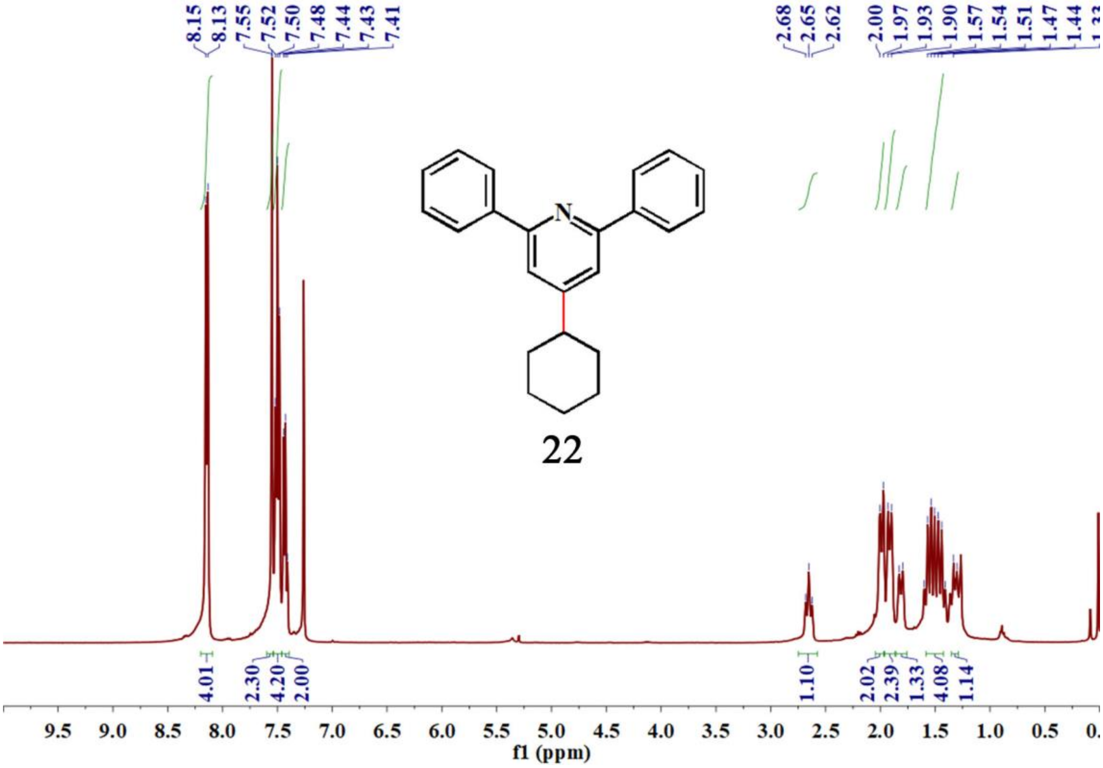

^{1}H NMR (400 MHz, $CDCl_3$) δ 8.14 (d, *J* = 7.8 Hz, 4H), 7.55 (s, 2H), 7.50 (t, *J* = 7.5 Hz, 4H), 7.43 (t, *J* = 7.0 Hz, 2H), 2.65 (t, *J* = 11.5 Hz, 1H), 1.99 (d, *J* = 12.5 Hz, 2H), 1.91 (d, *J* = 12.5 Hz, 2H), 1.81 (d, *J* = 12.8 Hz, 1H), 1.58 – 1.42 (m, 4H), 1.32 (d, *J* = 12.2 Hz, 1H).

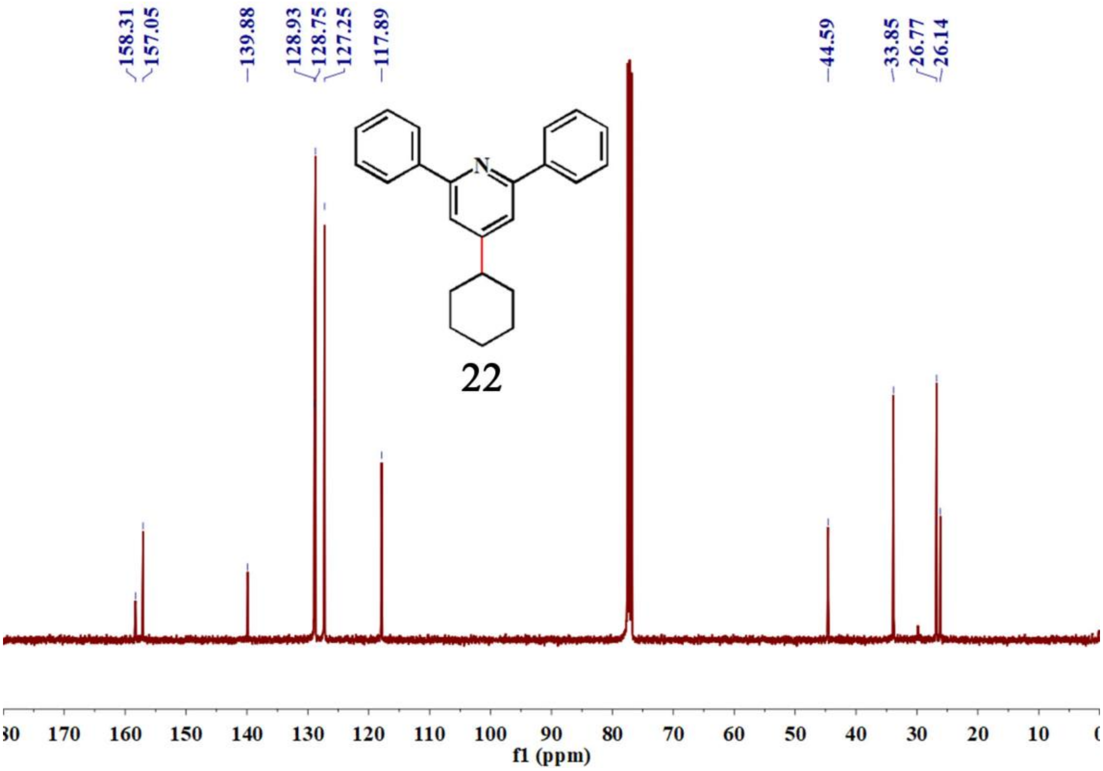

^{13}C NMR (101 MHz, $CDCl_3$) δ 158.31, 157.05, 139.88, 128.93, 128.75, 127.25, 117.89, 44.59, 33.85, 26.77, 26.14.

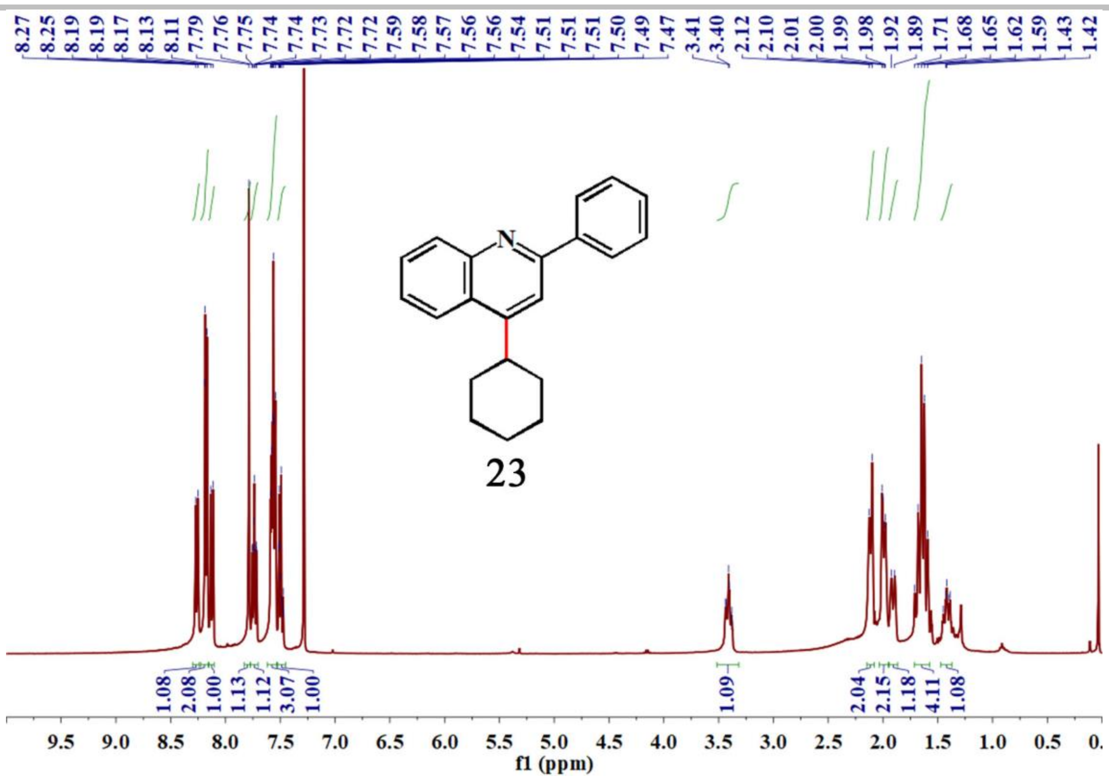

^{1}H NMR (400 MHz, $CDCl_3$) δ 8.26 (d, *J* = 8.3 Hz, 1H), 8.23 – 8.16 (m, 2H), 8.12 (d, *J* = 8.4 Hz, 1H), 7.79 (s, 1H), 7.74 (ddd, *J* = 8.3, 6.9, 1.3 Hz, 1H), 7.57 (td, *J* = 7.4, 4.7 Hz, 3H), 7.52 – 7.45 (m, 1H), 3.40 (ddd, *J* = 11.3, 8.5, 2.8 Hz, 1H), 2.11 (d, *J* = 10.5 Hz, 2H), 1.99 (dd, *J* = 9.7, 2.8 Hz, 2H), 1.91 (d, *J* = 11.5 Hz, 1H), 1.71 – 1.58 (m, 4H), 1.47 – 1.37 (m, 1H).

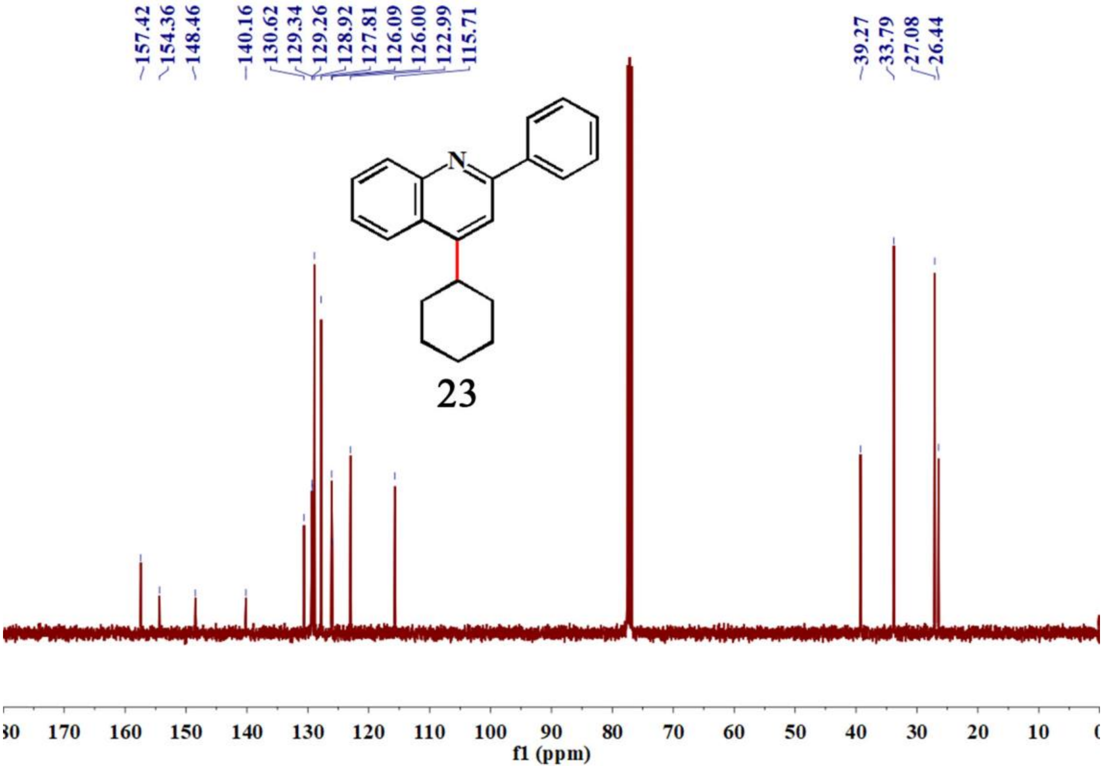

^{13}C NMR (101 MHz, $CDCl_3$) δ 157.42, 154.36, 148.46, 140.16, 130.62, 129.34, 129.26, 128.92, 127.81, 126.09, 126.00, 122.99, 115.71, 39.27, 33.79, 27.08, 26.44.

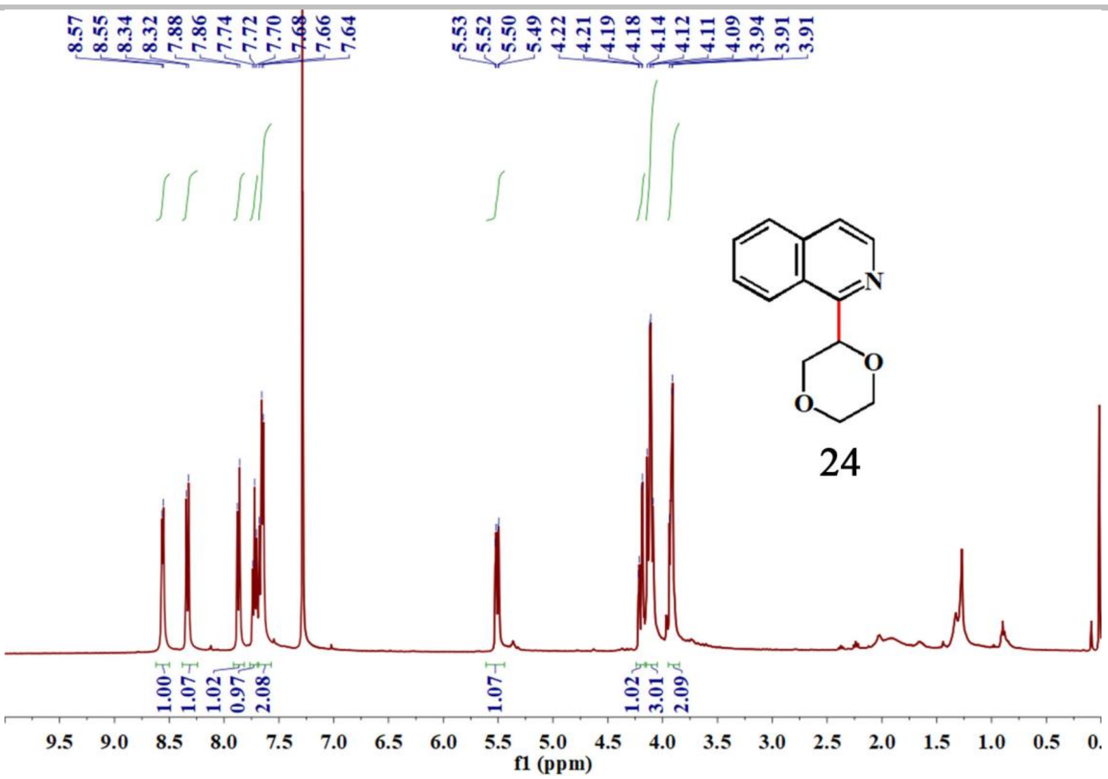

^{1}H NMR (400 MHz, $CDCl_3$) δ 8.56 (d, *J* = 5.5 Hz, 1H), 8.33 (d, *J* = 8.3 Hz, 1H), 7.87 (d, *J* = 8.1 Hz, 1H), 7.72 (t, *J* = 7.0 Hz, 1H), 7.68 – 7.57 (m, 2H), 5.51 (dd, *J* = 9.7, 2.6 Hz, 1H), 4.20 (dd, *J* = 11.8, 2.7 Hz, 1H), 4.11 (dd, *J* = 12.5, 9.2 Hz, 3H), 3.95 – 3.85 (m, 2H).

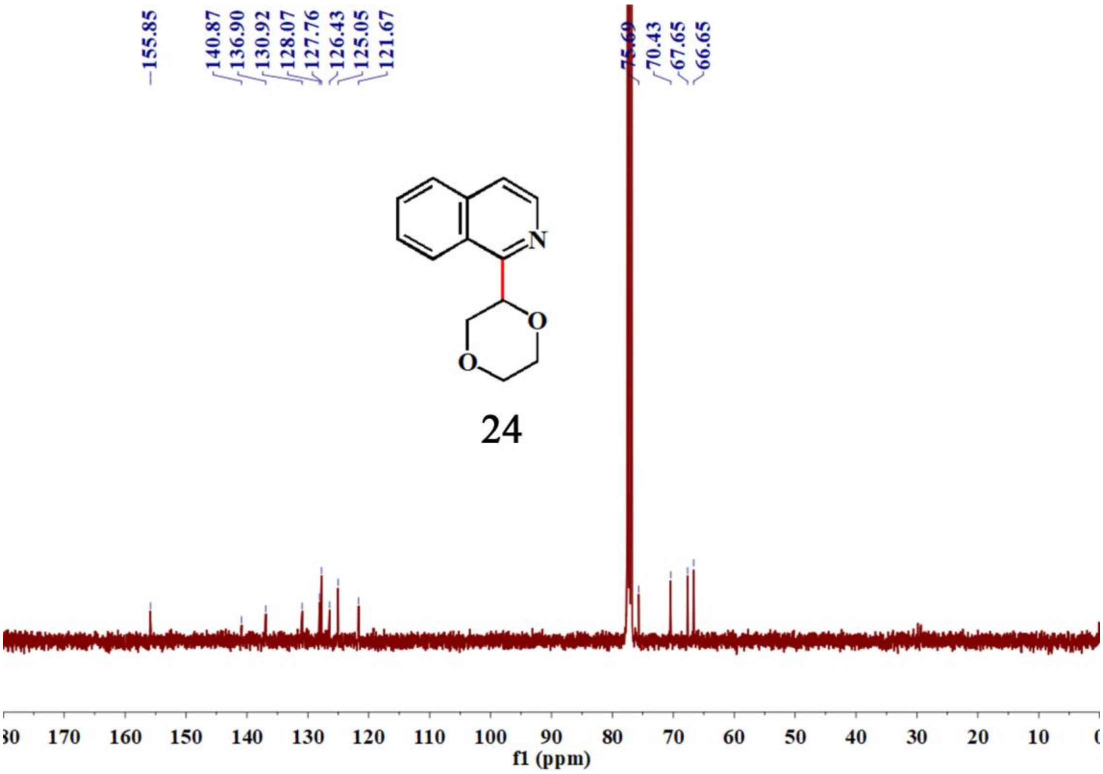

^{13}C NMR (101 MHz, $CDCl_3$) δ 155.85, 140.87, 136.90, 130.92, 128.07, 127.76, 126.43, 125.05, 121.67, 75.69, 70.43, 67.65, 66.65.

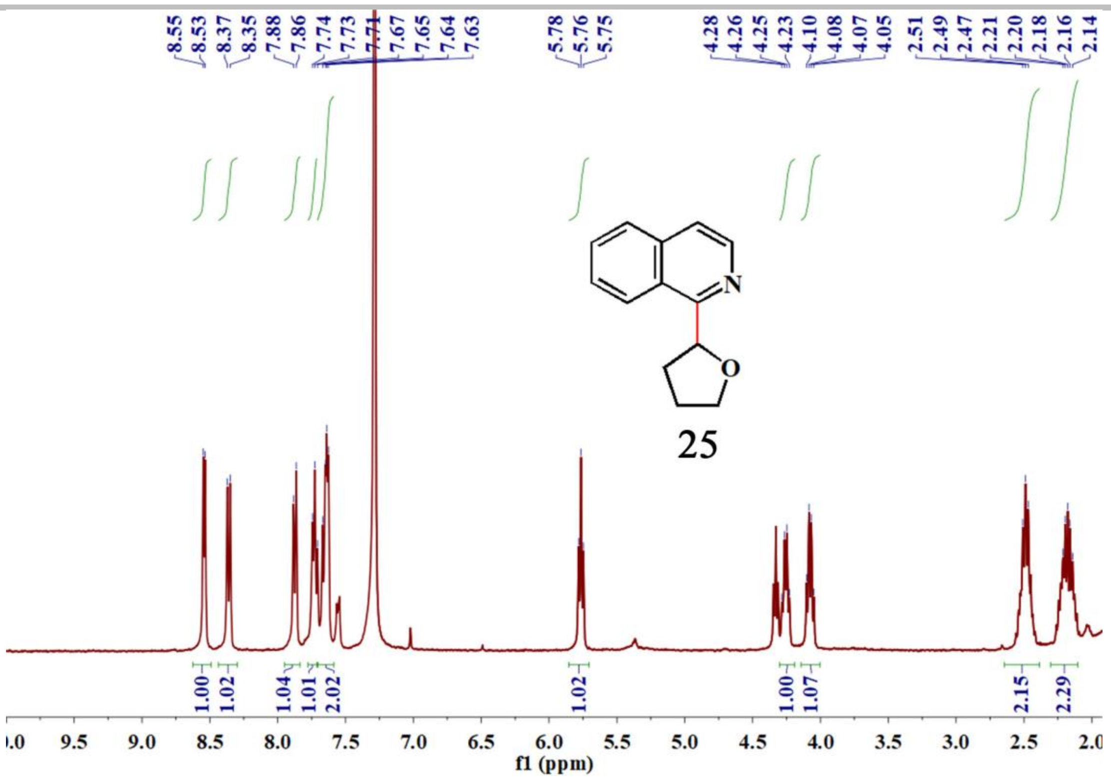

^{1}H NMR (400 MHz, $CDCl_3$) δ 8.54 (d, *J* = 5.7 Hz, 1H), 8.36 (d, *J* = 8.4 Hz, 1H), 7.87 (d, *J* = 8.2 Hz, 1H), 7.73 (d, *J* = 6.9 Hz, 1H), 7.64 (dd, *J* = 10.2, 7.0 Hz, 2H), 5.76 (t, *J* = 7.1 Hz, 1H), 4.26 (dd, *J* = 14.5, 7.4 Hz, 1H), 4.08 (dd, *J* = 14.2, 7.7 Hz, 1H), 2.64 – 2.39 (m, 2H), 2.30 – 2.10 (m, 2H).

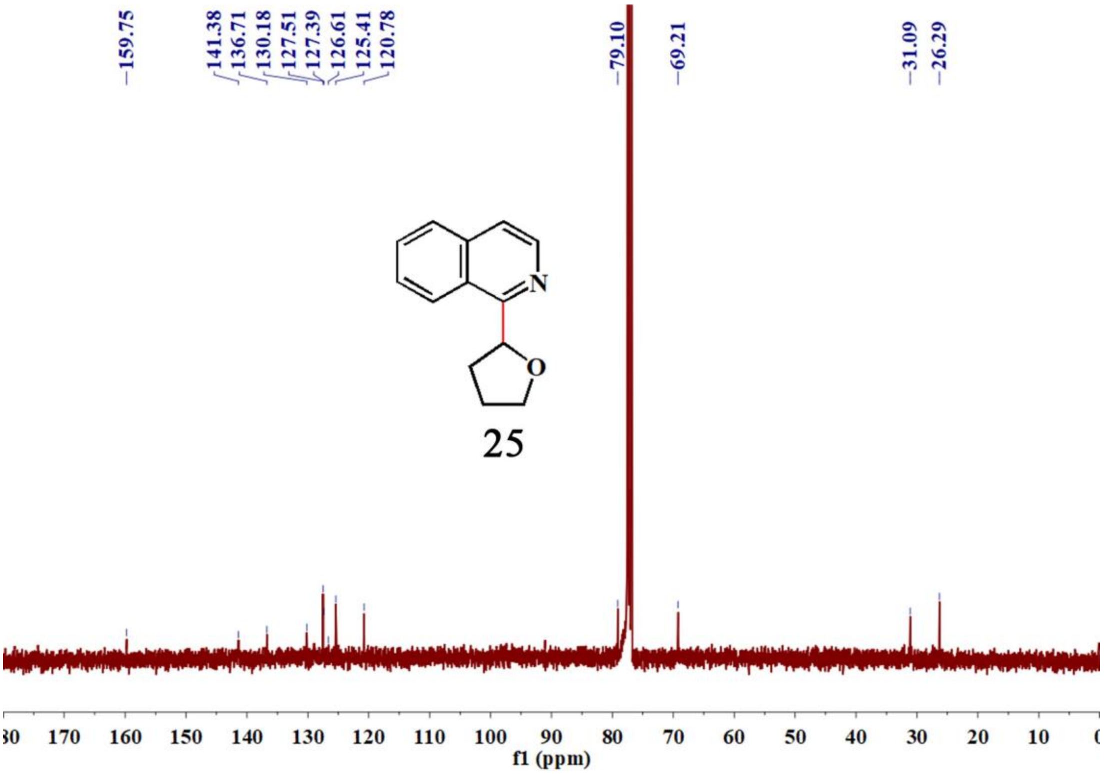

^{13}C NMR (101 MHz, $CDCl_3$) δ 159.75, 141.38, 136.71, 130.18, 127.51, 127.39, 126.61, 125.41, 120.78, 79.10, 69.21, 31.09, 26.29.

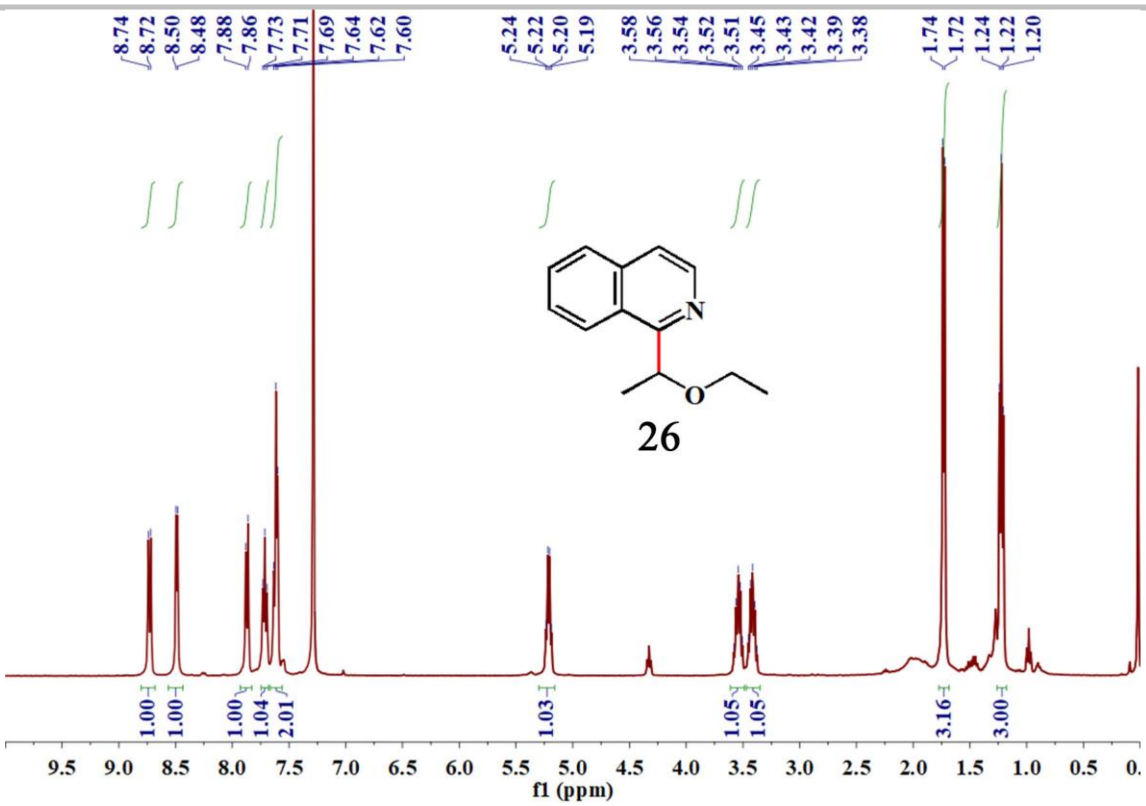

^{1}H NMR (400 MHz, $CDCl_3$) δ 8.73 (d, *J* = 8.5 Hz, 1H), 8.49 (d, *J* = 5.6 Hz, 1H), 7.87 (d, *J* = 8.1 Hz, 1H), 7.71 (t, *J* = 7.4 Hz, 1H), 7.62 (t, *J* = 7.0 Hz, 2H), 5.21 (q, *J* = 6.7 Hz, 1H), 3.61 – 3.49 (m, 1H), 3.47 – 3.35 (m, 1H), 1.73 (d, *J* = 6.7 Hz, 3H), 1.22 (t, *J* = 7.0 Hz, 3H).

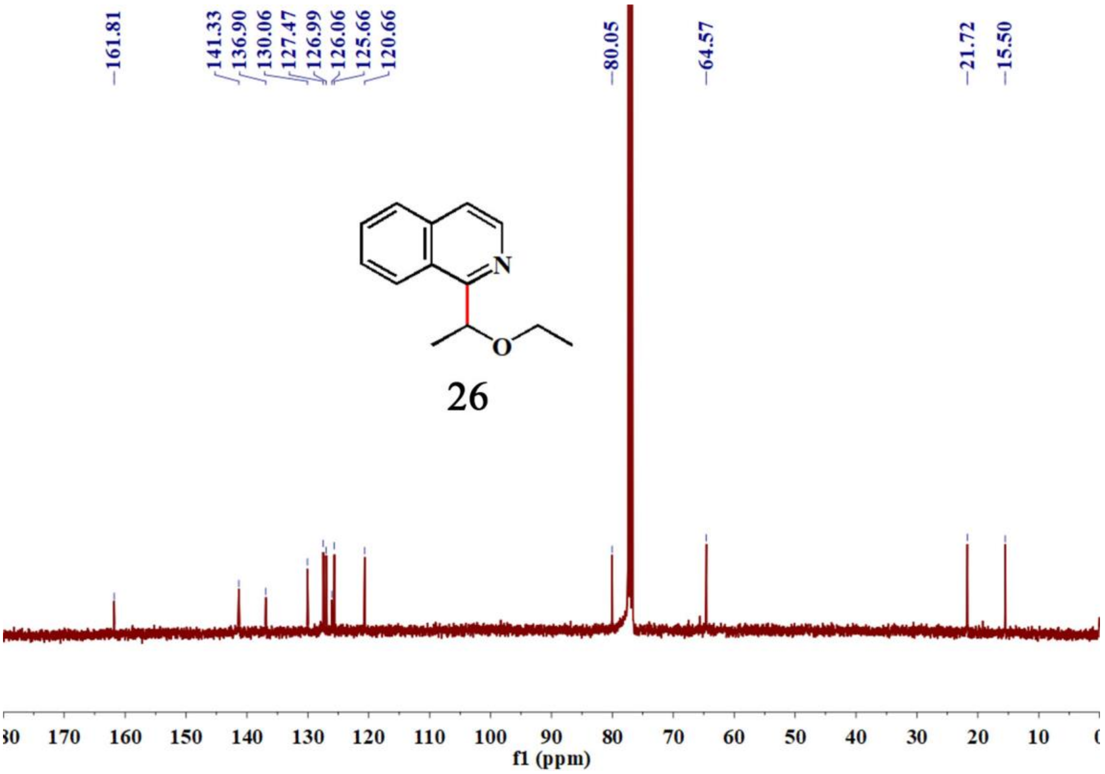

^{13}C NMR (101 MHz, $CDCl_3$) δ 161.81, 141.33, 136.90, 130.06, 127.47, 126.99, 126.06, 125.66, 120.66, 80.05, 64.57, 21.72, 15.50.

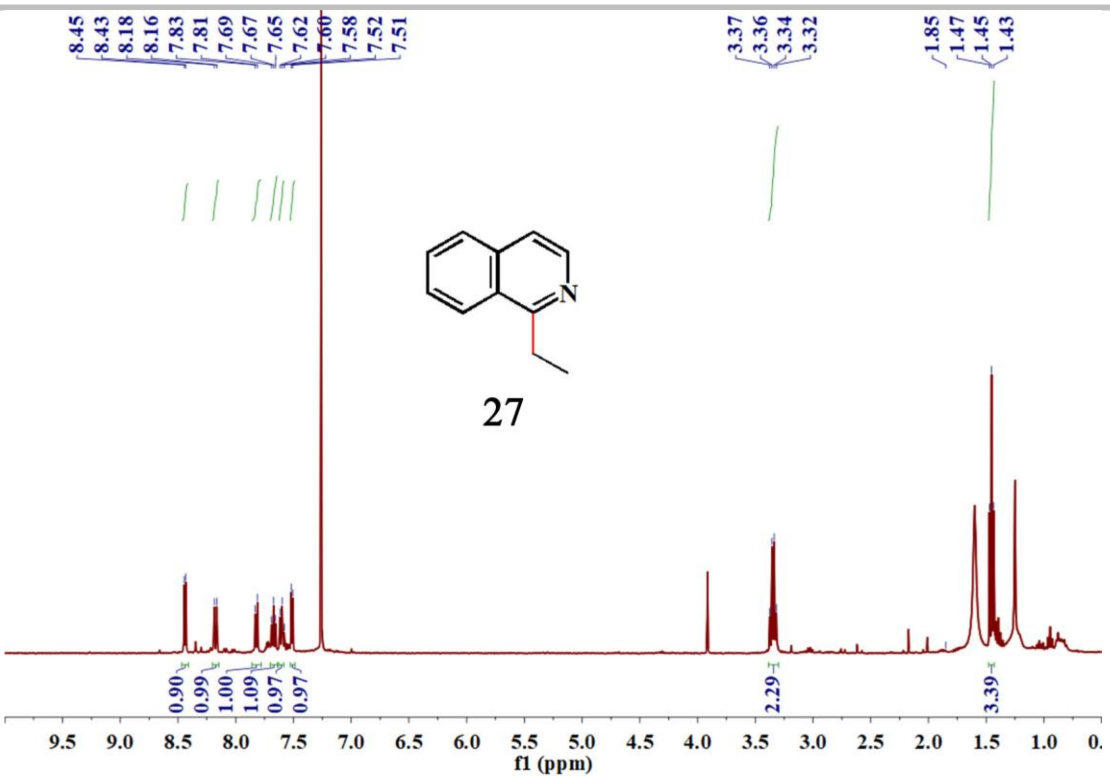

^{1}H NMR (400 MHz, $CDCl_3$) δ 8.44 (d, *J* = 5.8 Hz, 1H), 8.17 (d, *J* = 8.3 Hz, 1H), 7.82 (d, *J* = 8.0 Hz, 1H), 7.67 (t, *J* = 7.0 Hz, 1H), 7.61 (d, *J* = 7.1 Hz, 1H), 7.51 (d, *J* = 5.8 Hz, 1H), 3.35 (q, *J* = 7.6 Hz, 2H), 1.45 (t, *J* = 7.6 Hz, 3H).

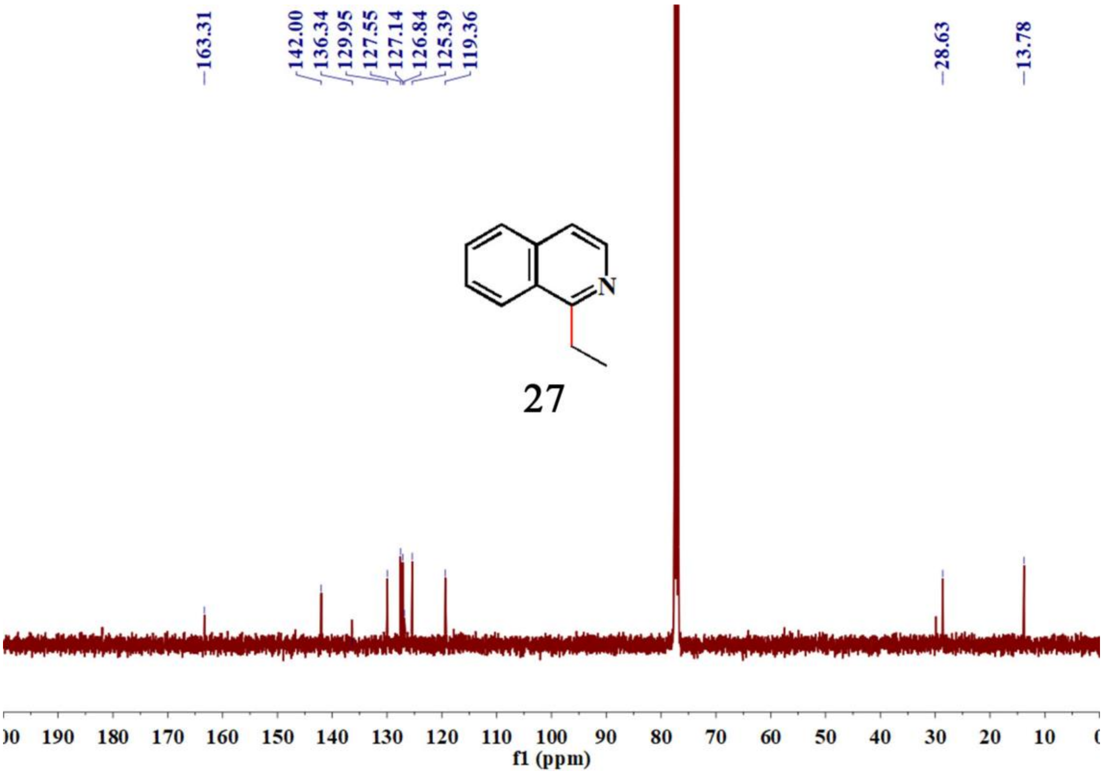

^{13}C NMR (101 MHz, $CDCl_3$) δ 163.31, 142.00, 136.34, 129.95, 127.55, 127.14, 126.84, 125.39, 119.36, 28.63, 13.78.

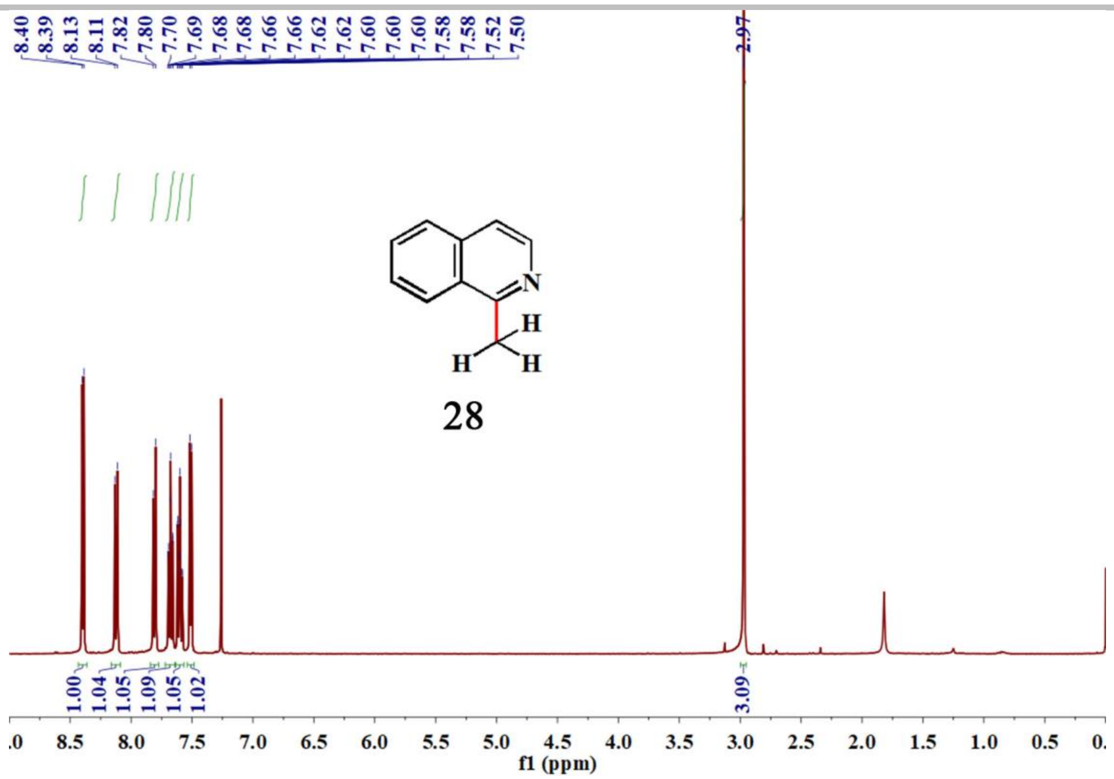

^{1}H NMR (400 MHz, $CDCl_3$) δ 8.40 (d, *J* = 5.8 Hz, 1H), 8.12 (d, *J* = 8.3 Hz, 1H), 7.81 (d, *J* = 8.1 Hz, 1H), 7.72 – 7.64 (m, 1H), 7.60 (ddd, *J* = 8.2, 7.0, 1.2 Hz, 1H), 7.51 (d, *J* = 5.8 Hz, 1H), 2.97 (s, 3H).

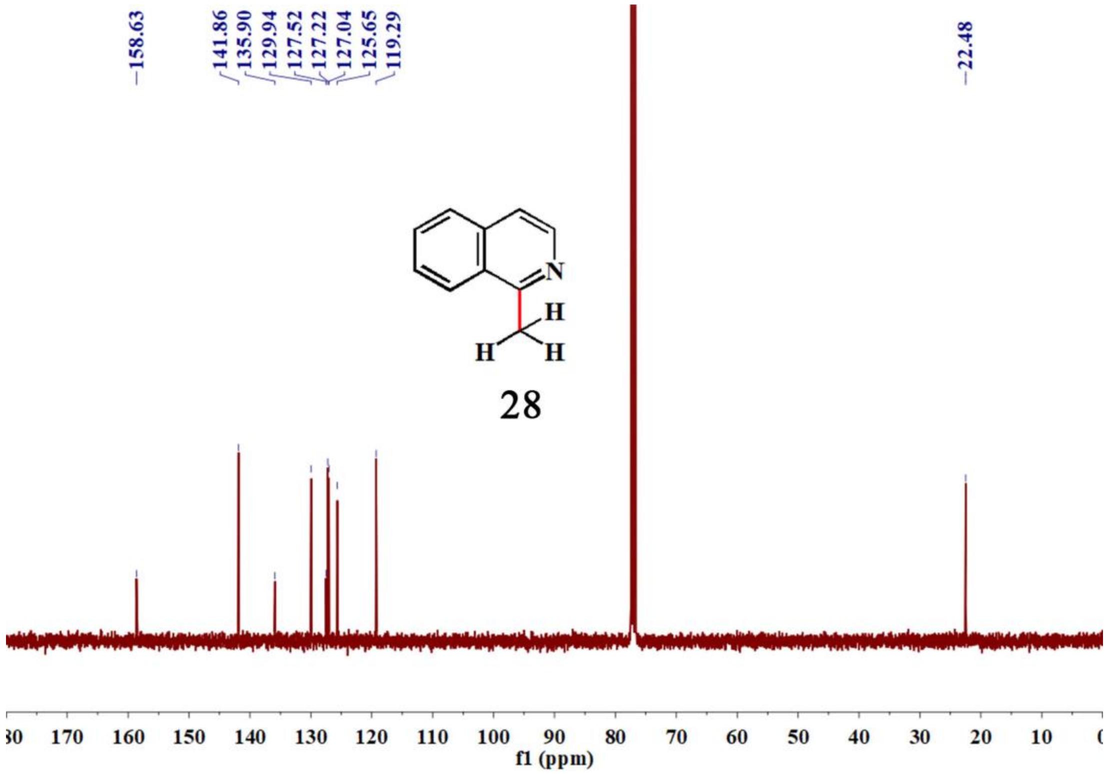

^{13}C NMR (101 MHz, $CDCl_3$) δ 158.63, 141.86, 135.90, 129.94, 127.52, 127.22, 127.04, 125.65, 119.29, 22.48.

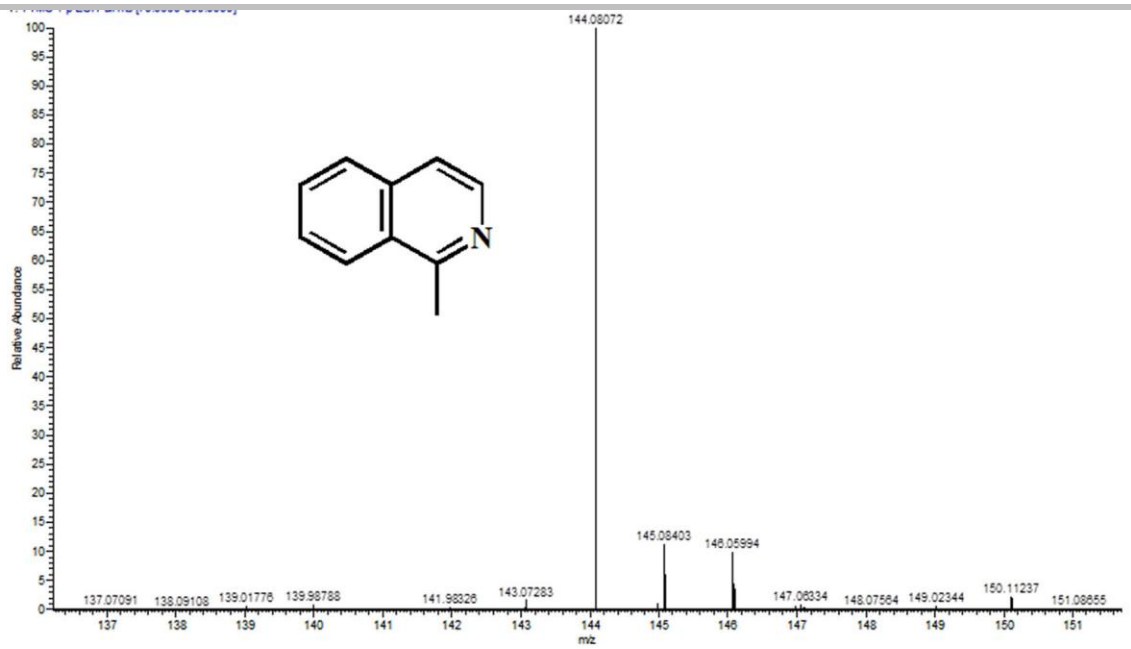

HRMS (ESI+): calcd for $C10H10N^+$ (M+H) 144.0808, found 144.0807.

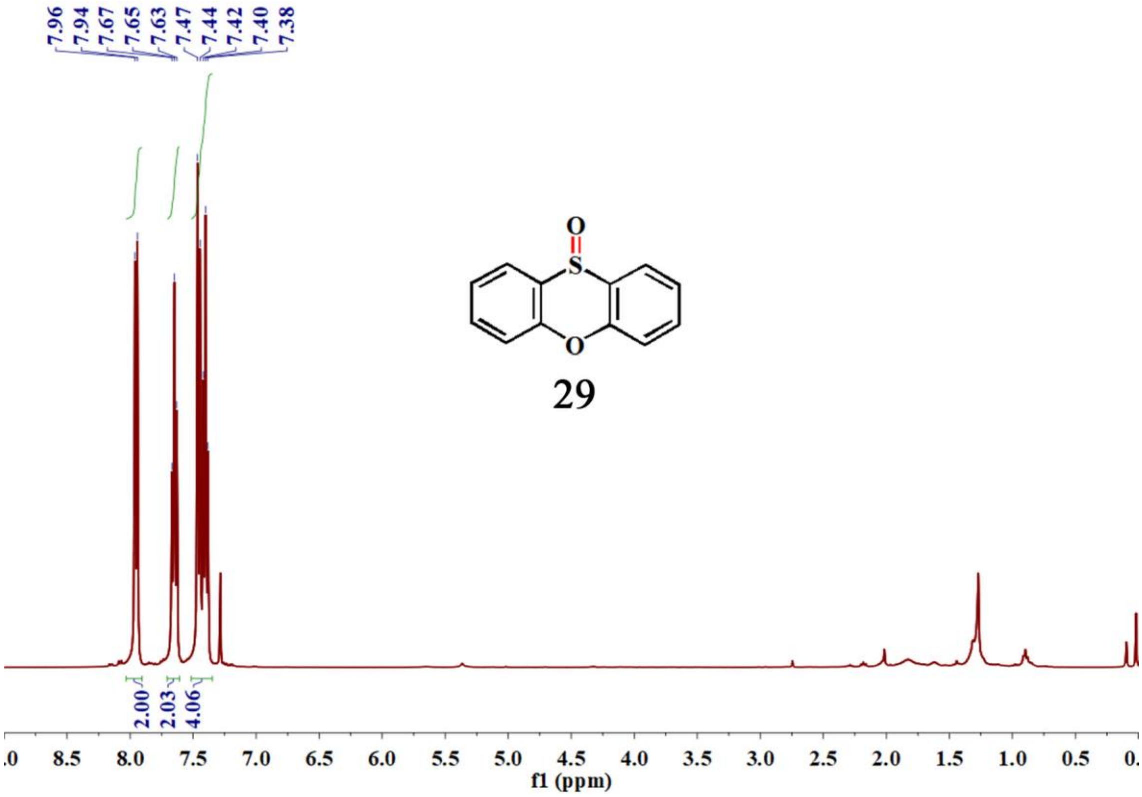

^1H NMR (400 MHz, $CDCl_3$) δ 7.95 (d, *J* = 7.8 Hz, 2H), 7.65 (t, *J* = 7.8 Hz, 2H), 7.51 – 7.35 (m, 4H).

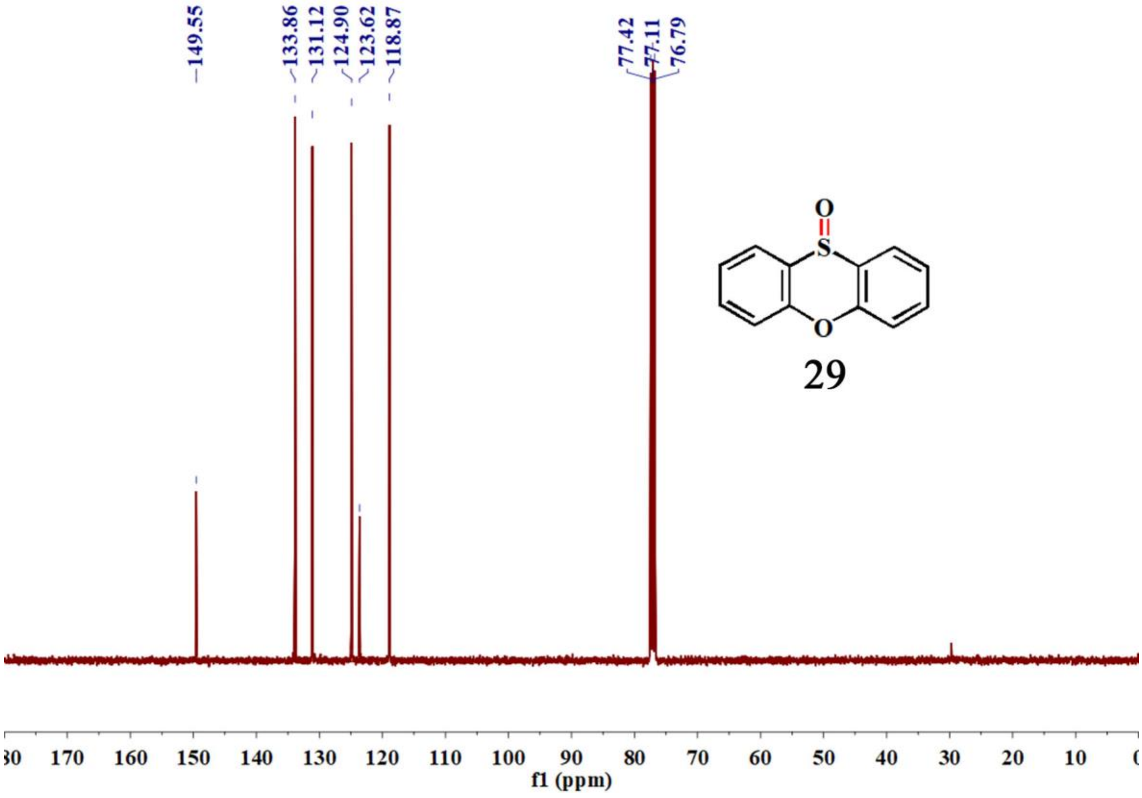

^{13}C NMR (101 MHz, $CDCl_3$) δ 149.55, 133.86, 131.12, 124.90, 123.62, 118.87.

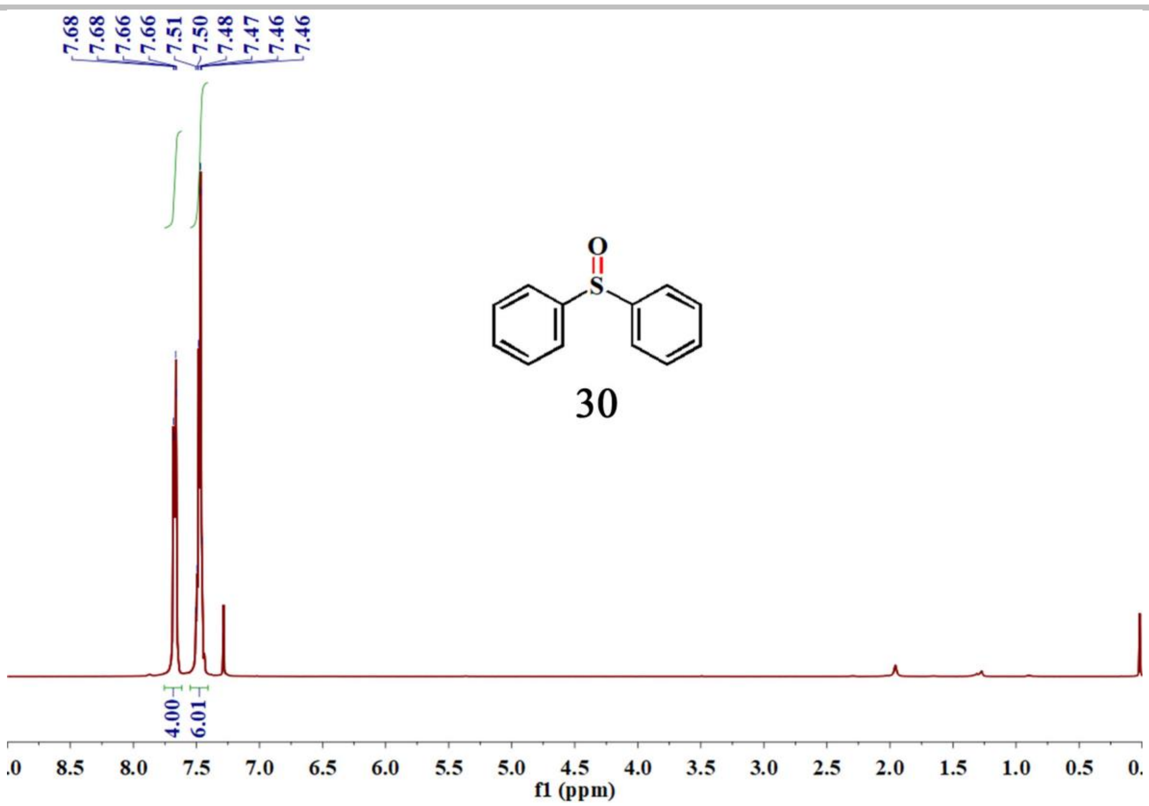

^{1}H NMR (400 MHz, $CDCl_3$) δ 7.67 (dd, J = 7.7, 1.8 Hz, 4H), 7.55 – 7.41 (m, 6H).

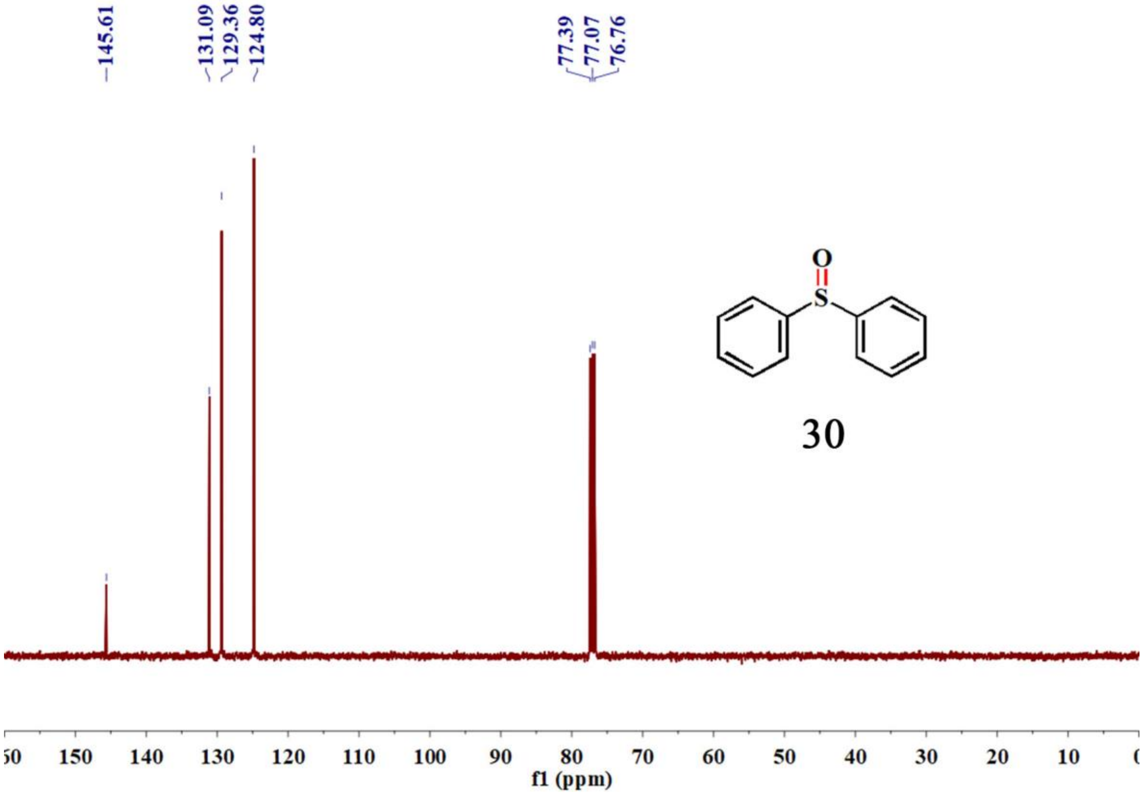

^{13}C NMR (101 MHz, $CDCl_3$) δ 145.61, 131.09, 129.36, 124.80.

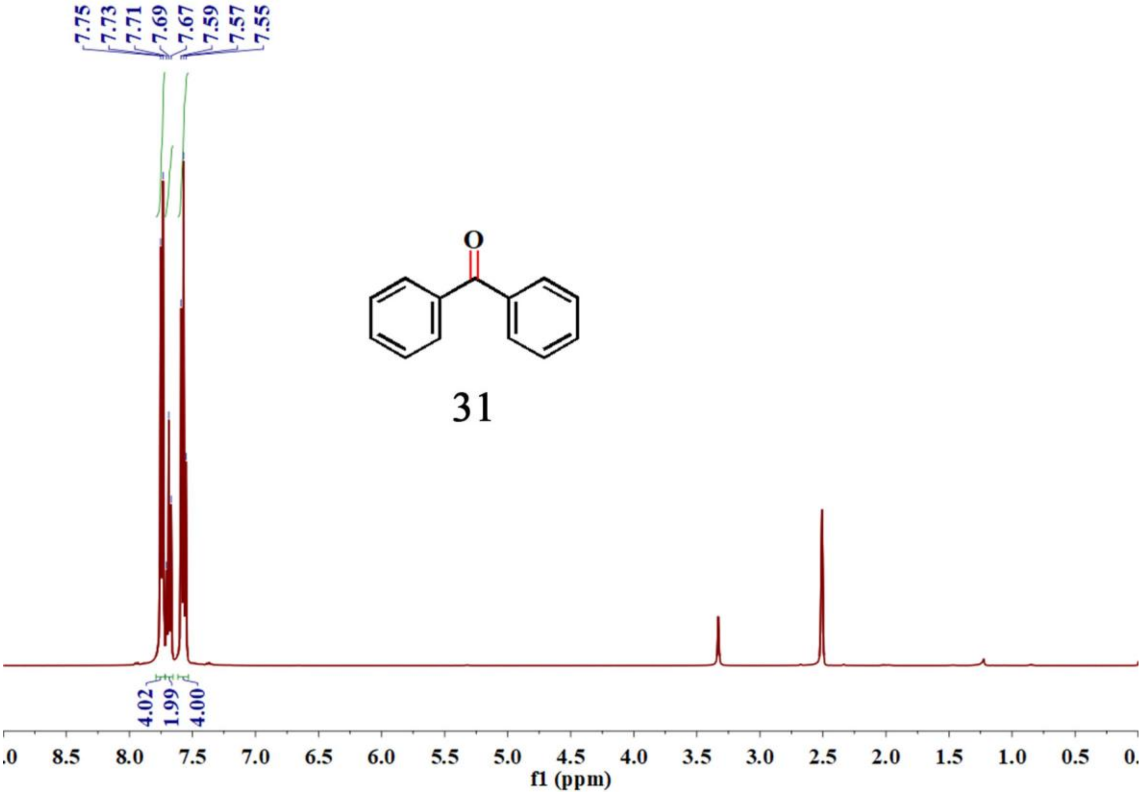

^{1}H NMR (400 MHz, DMSO_D$_6$) δ 7.74 (d, *J* = 7.7 Hz, 4H), 7.69 (t, *J* = 7.4 Hz, 2H), 7.57 (t, *J* = 7.6 Hz, 4H).

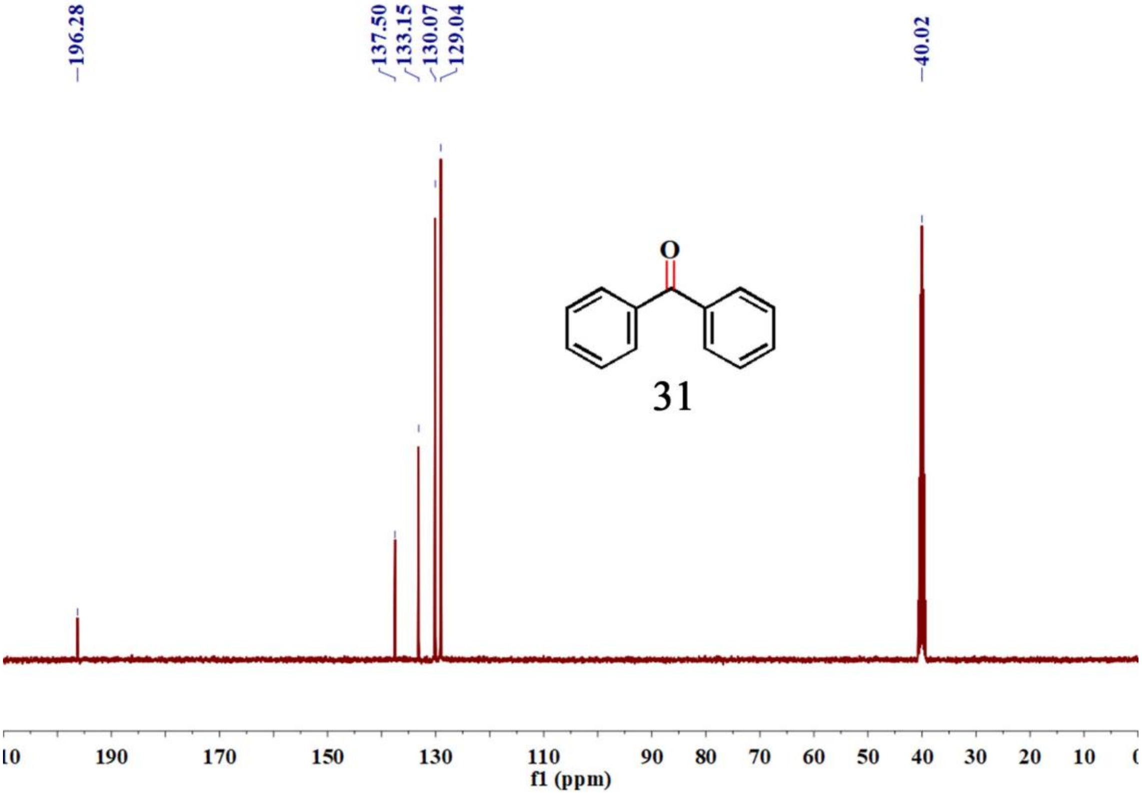

^{13}C NMR (101 MHz, DMSO_D$_6$) δ 196.28, 137.50, 133.15, 130.07, 129.04.

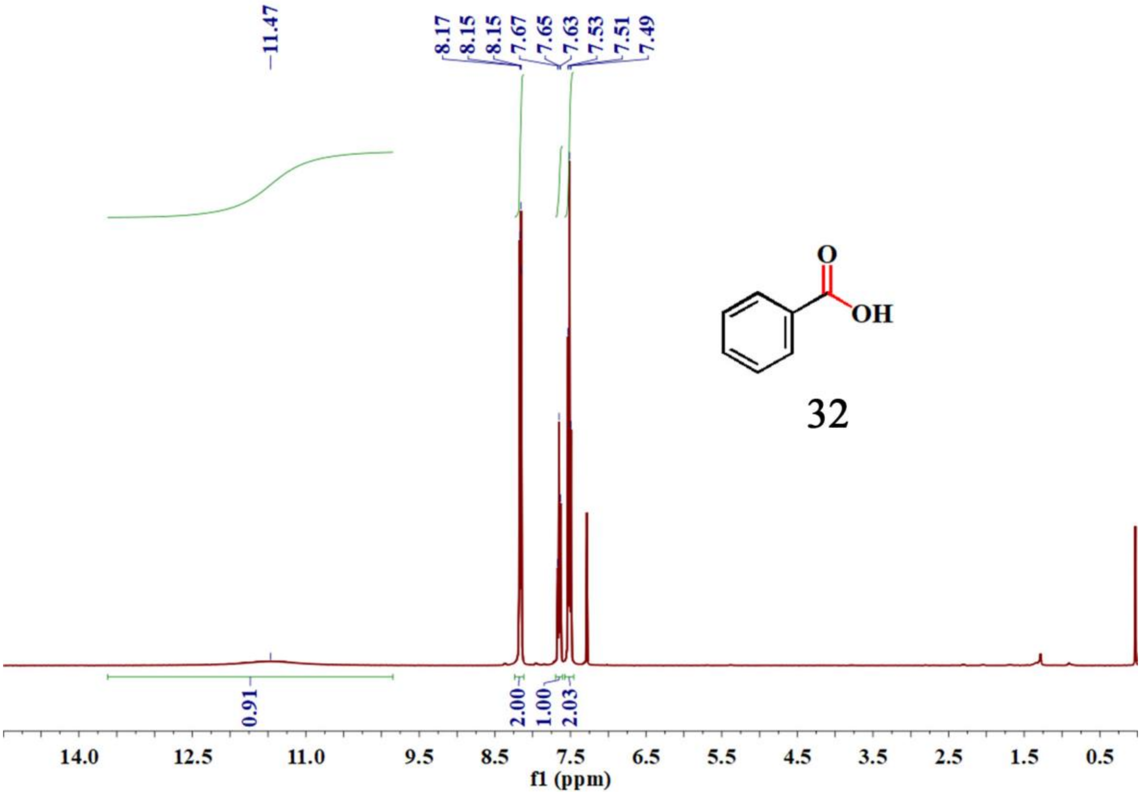

^{1}H NMR (400 MHz, $CDCl_3$) δ 11.47 (s, 1H), 8.24 – 8.12 (m, 2H), 7.65 (t, *J* = 7.4 Hz, 1H), 7.51 (t, *J* = 7.7 Hz, 2H).

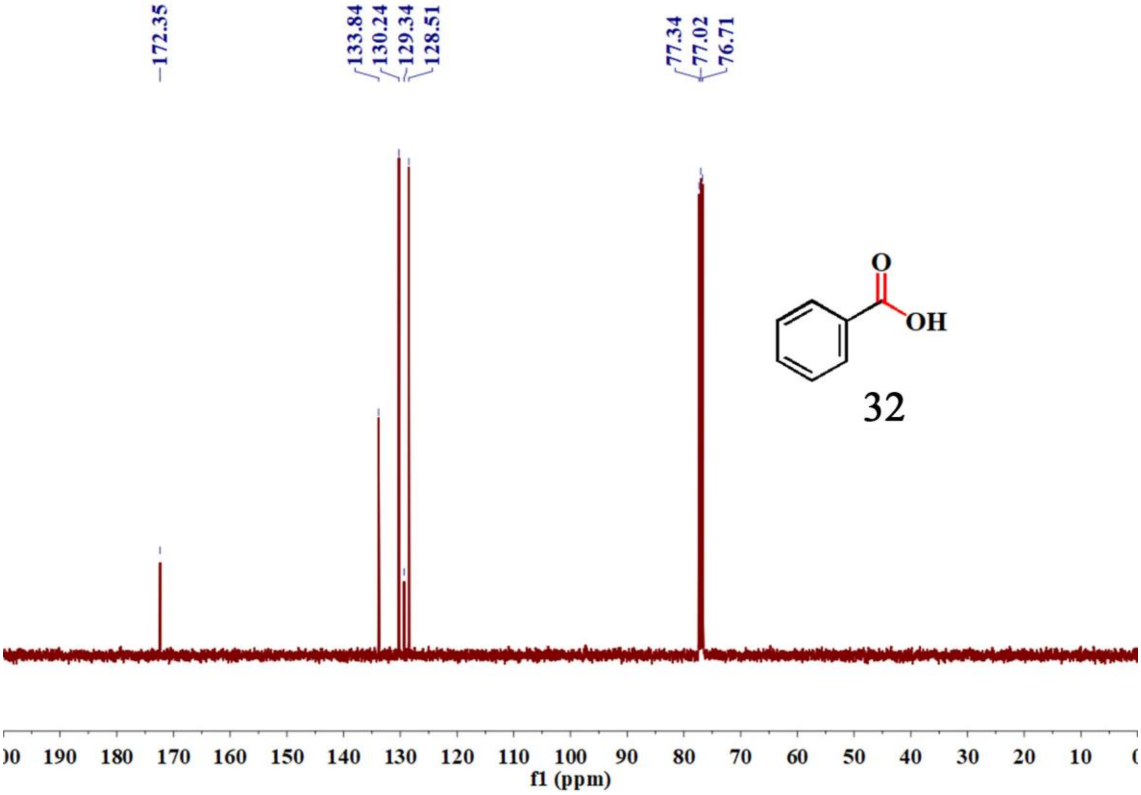

^{13}C NMR (101 MHz, $CDCl_3$) δ 172.35, 133.84, 130.24, 129.34, 128.51.

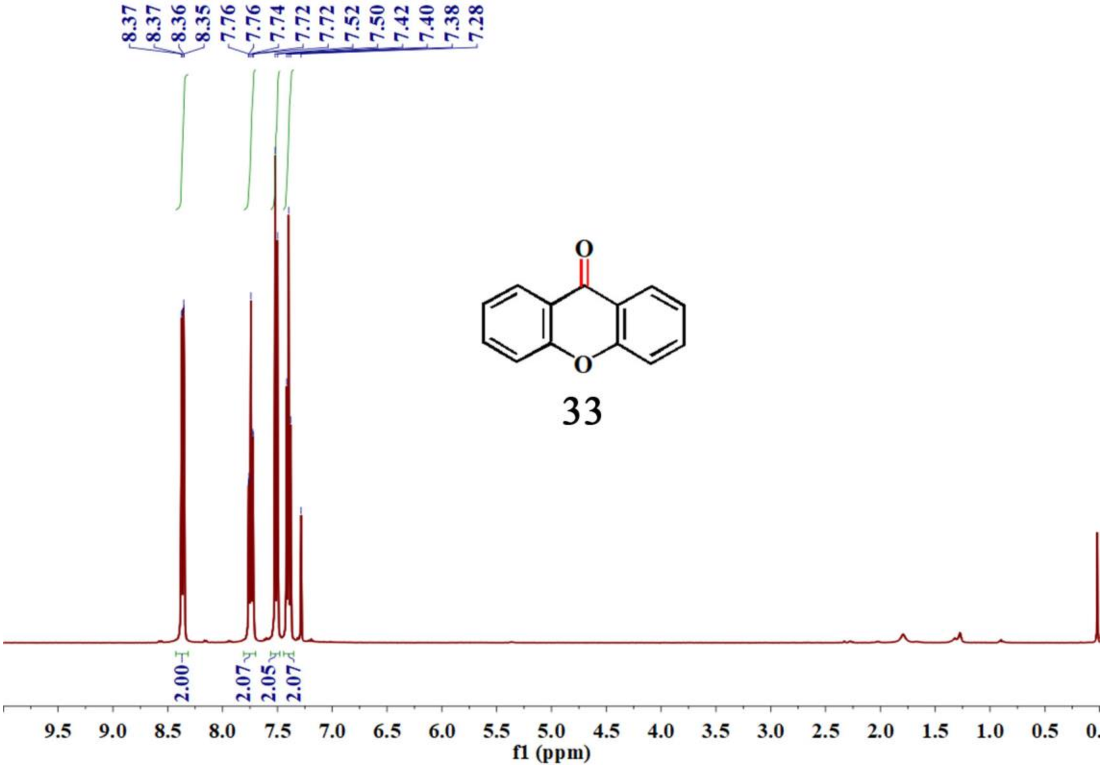

^{1}H NMR (400 MHz, $CDCl_3$) δ 8.36 (dd, *J* = 7.9, 1.4 Hz, 2H), 7.81 – 7.70 (m, 2H), 7.51 (d, *J* = 8.4 Hz, 2H), 7.40 (t, *J* = 7.5 Hz, 2H).

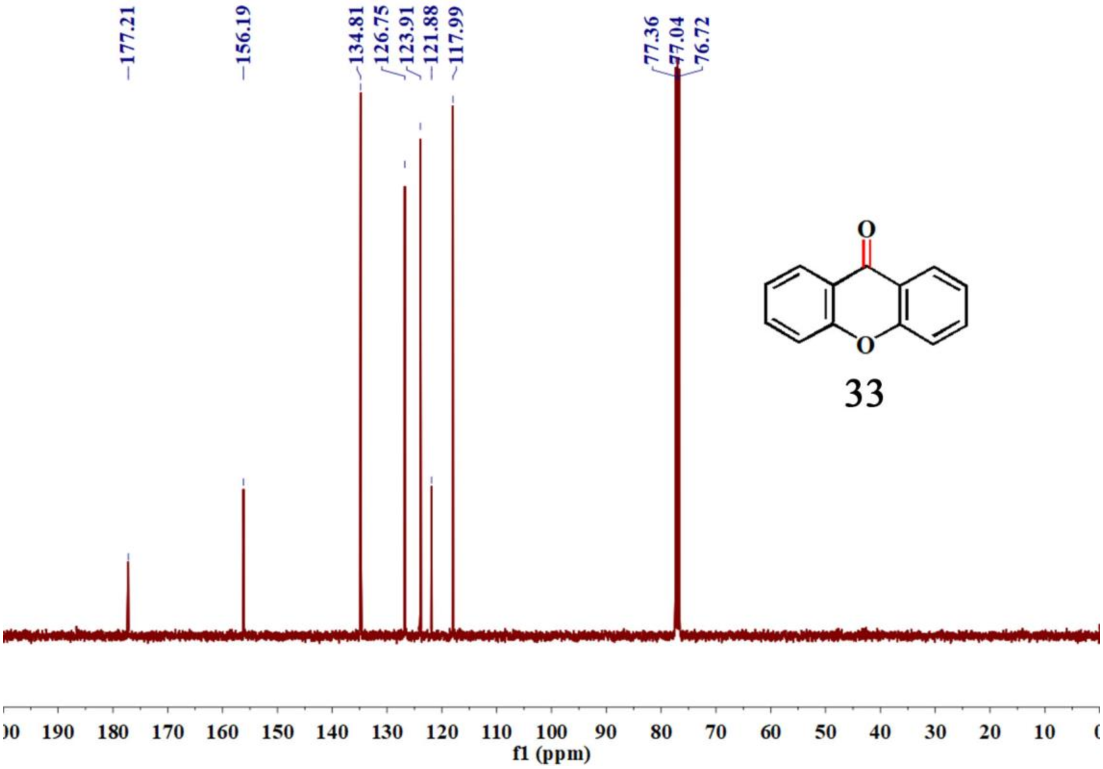

^{13}C NMR (101 MHz, $CDCl_3$) δ 177.21, 156.19, 134.81, 126.75, 123.91, 121.88, 117.99.

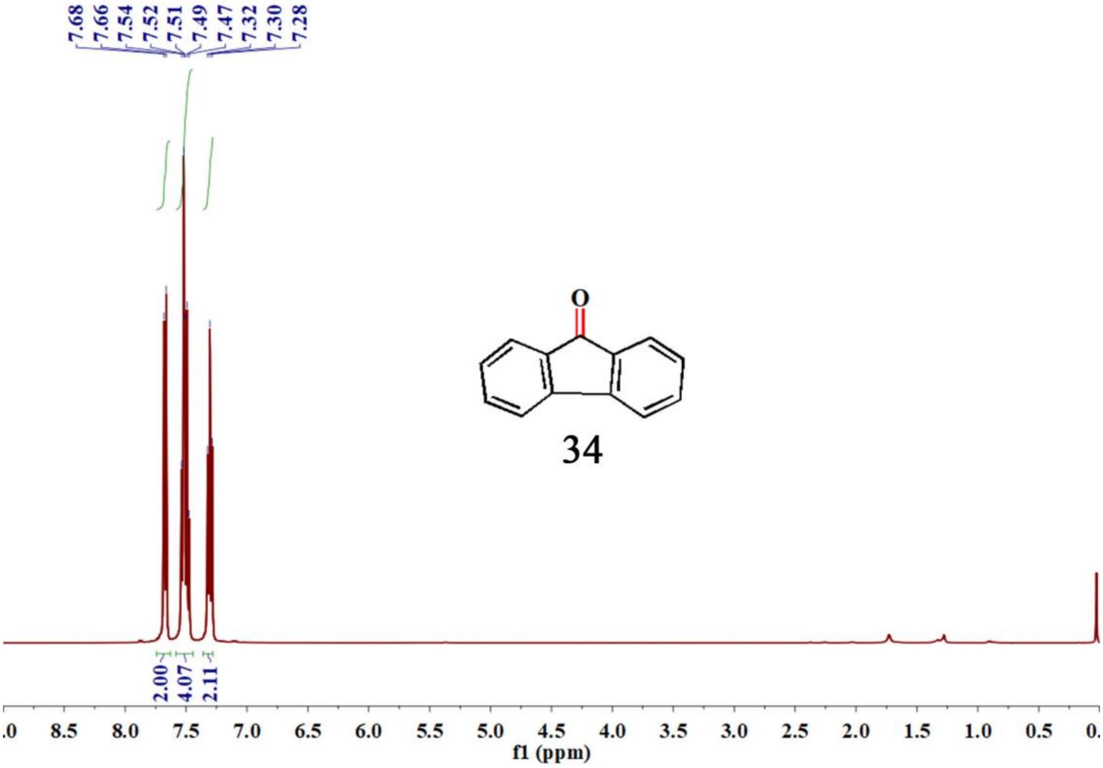

^1H NMR (400 MHz, $CDCl_3$) δ 7.67 (d, *J* = 7.3 Hz, 2H), 7.51 (dt, *J* = 14.6, 7.2 Hz, 4H), 7.30 (t, *J* = 7.7 Hz, 2H).

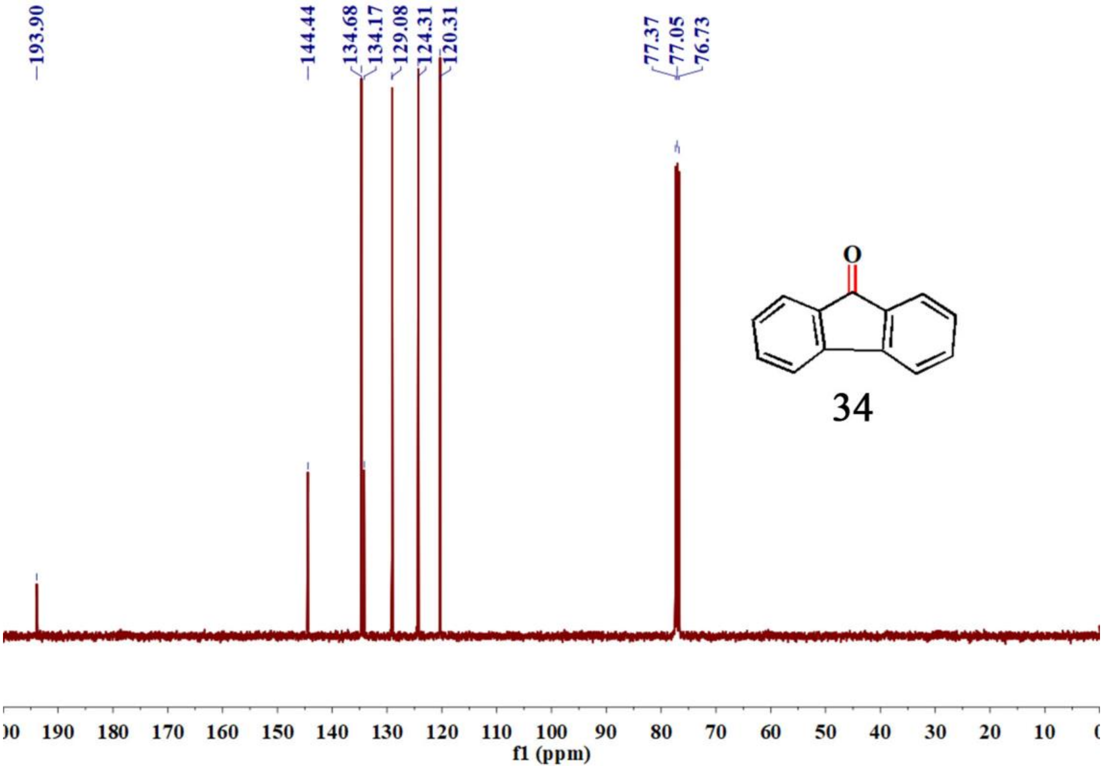

^{13}C NMR (101 MHz, $CDCl_3$) δ 193.90, 144.44, 134.68, 134.17, 129.08, 124.31, 120.31.

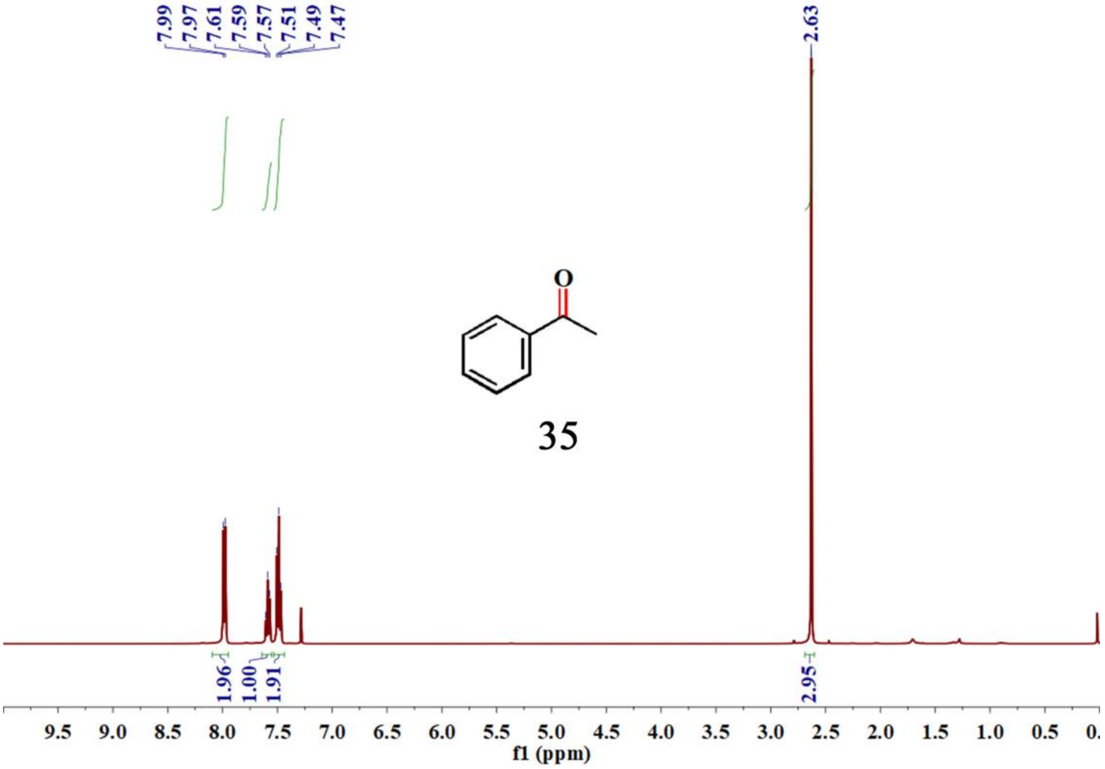

^{1}H NMR (400 MHz, $CDCl_3$) δ 7.98 (d, *J* = 7.3 Hz, 2H), 7.59 (t, *J* = 7.4 Hz, 1H), 7.49 (t, *J* = 7.6 Hz, 2H), 2.63 (s, 3H).

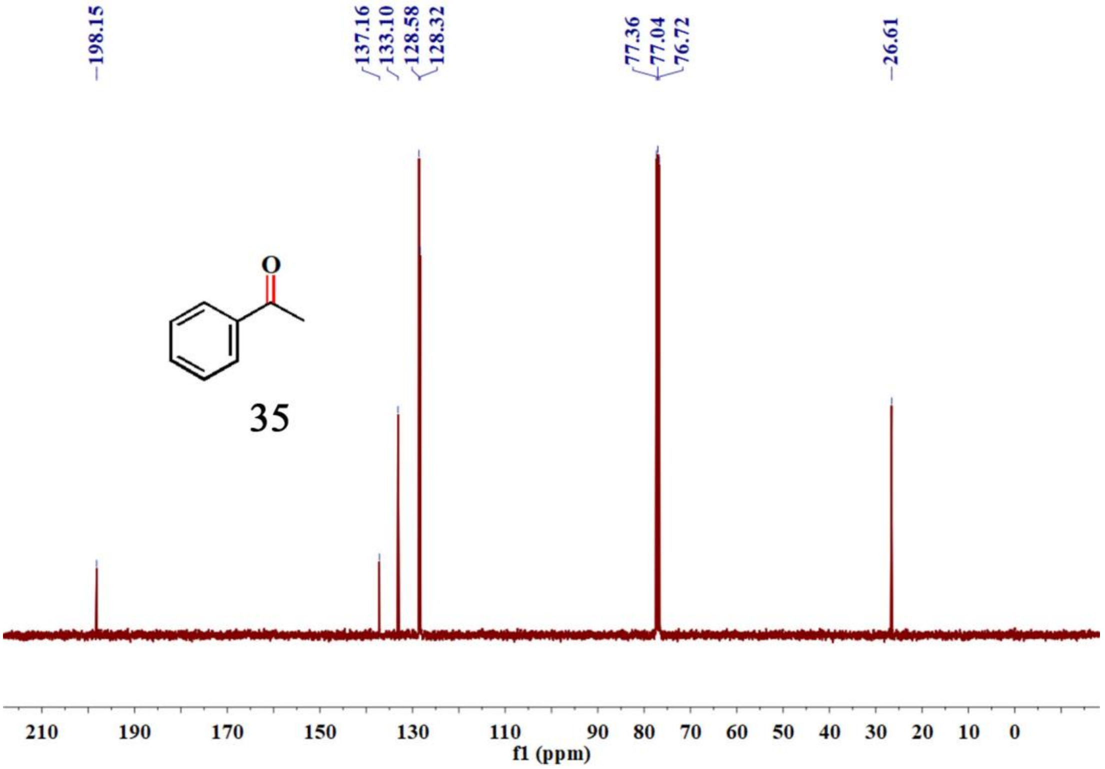

^{13}C NMR (101 MHz, $CDCl_3$) δ 198.15, 137.16, 133.10, 128.58, 128.32, 26.61.

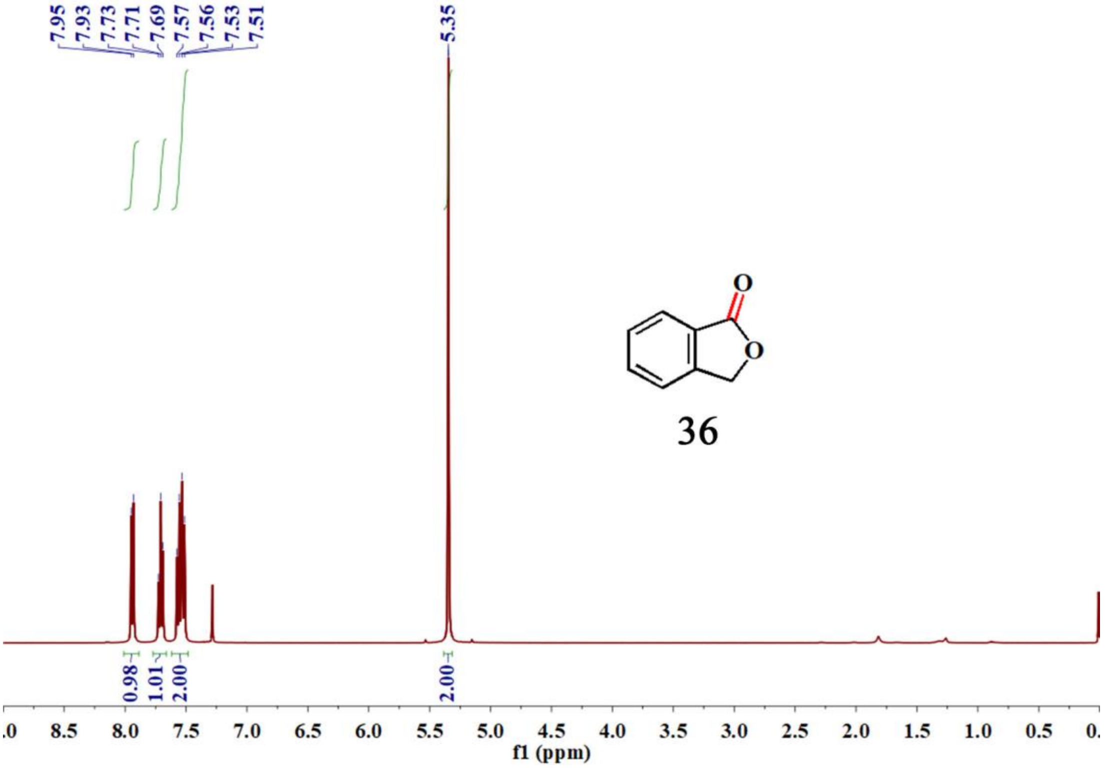

^{1}H NMR (400 MHz, $CDCl_3$) δ 7.94 (d, *J* = 7.7 Hz, 1H), 7.71 (t, *J* = 7.5 Hz, 1H), 7.54 (dd, *J* = 16.9, 7.7 Hz, 2H), 5.35 (s, 2H).

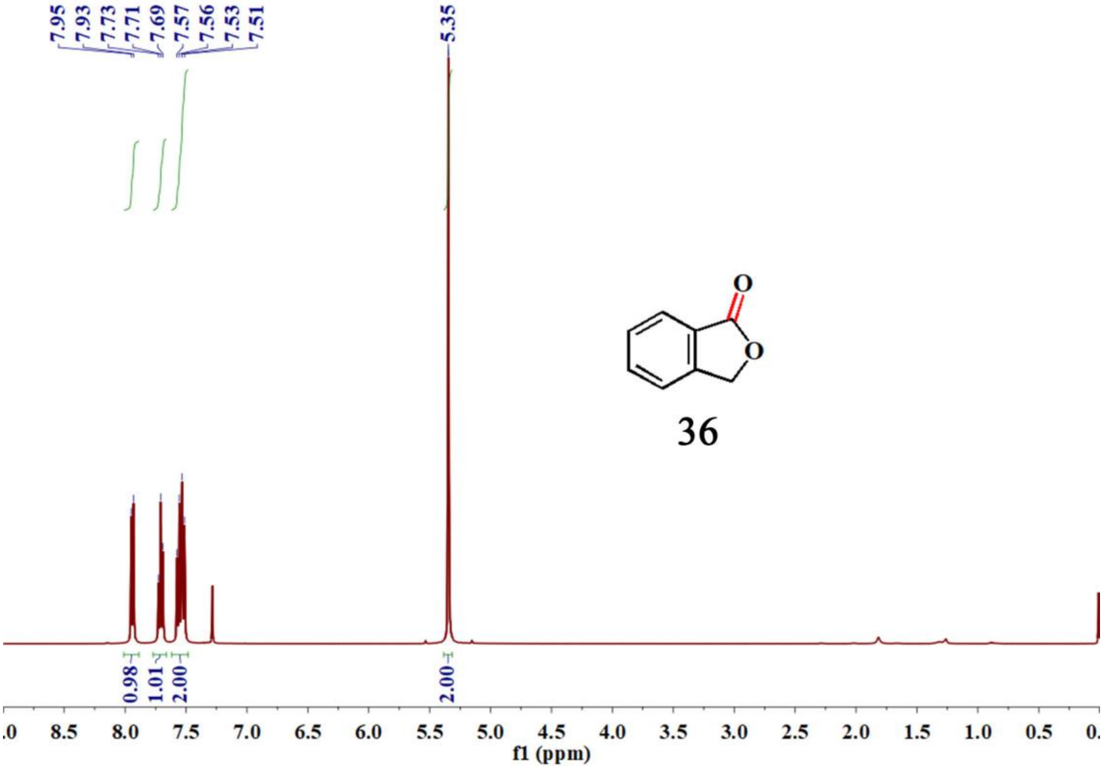

^{13}C NMR (101 MHz, $CDCl_3$) δ 171.14, 146.55, 134.05, 129.06, 125.78, 122.14, 69.69.

13 References

[1] P.-Y. Wu, Y. Li, J.-J. Zheng, N. Hosono, K.-i. Otake, J. Wang, Y.-H. Liu, L.-L. Xia, M. Jiang, S. Sakaki, S. Kitagawa, *Nat. Commun.* **2019**, *10*, 4362.

[2] D.-W. Feng, K.-C. Wang, Z.-W. Wei, Y.-P. Chen, C. M. Simon, R. K. Arvapally, R. L. Martin, M. Bosch, T.-F. Liu, S. Fordham, D.-Q. Yuan, M. A. Omary, M. Haranczyk, B. Smit, H.-C. Zhou, *Nat. Commun.* **2014**, *5*.

[3] Y.-H. Jin, Q.-Q. Zhang, L.-F. Wang, X.-Y. Wang, C.-G. Meng, C.-Y. Duan, *Green Chem.* **2021**, *23*, 6984-6989.
